# Discovery and Biosynthesis of Farnesyl Pyrophosphate-Derived Noncanonical C_17_ Terpenes from *Pseudomonas* Species

**DOI:** 10.1021/jacs.5c21930

**Published:** 2026-03-16

**Authors:** Qing-Yin Pu, Xu-Hua Mo, Tilo Lübken, Manuel Einsiedler, Tobias A. M. Gulder

**Affiliations:** 1 Chair of Technical Biochemistry, Technische Universität Dresden, Bergstraße 66, Dresden 01069, Germany; 2 Shandong Provincial Key Laboratory of Microbial Resource Exploration and Innovative Utilization, School of Life Sciences, 98431 Qingdao Agricultural University, Qingdao 266109, China; 3 Chair of Organic Chemistry I, Technische Universität Dresden, Bergstraße 66, Dresden 01069, Germany; 4 Department of Natural Product Biotechnology, Helmholtz Institute for Pharmaceutical Research Saarland (HIPS), Helmholtz Centre for Infection Research (HZI) and Department of Pharmacy at Saarland University, PharmaScienceHub (PSH), Campus E8.1, Saarbrücken 66123, Germany

## Abstract

Terpenes make up a structurally and functionally highly diverse class of natural products found across all living organisms. *Pseudomonas* species possess significant potential for the biosynthetic assembly of farnesyl pyrophosphate (FPP)-derived terpenes with unusual carbon skeletons. However, their structural and biosynthetic diversity has been largely unexplored. Here, we report the discovery of grimophan, a C_17_ terpene featuring a rare deltacyclane skeleton. The compound was accessed by heterologous expression of the *pgr* biosynthetic gene cluster from *Pseudomonas grimontii* DSM 17515 in *E. coli*. The roles of the enzymes involved in grimophan biosynthesis were elucidated through *in vitro* reconstitution of the entire biosynthetic pathway. In-depth functional studies on the involved SAM-dependent methyltransferases led to the discovery of methyltransferase-like enzymes that do not perform functional group transfer, but rather significantly enhance the production titer of noncanonical terpenes. These enzymes thus constitute valuable tools for enhancing biotechnological production levels of noncanonical C_16_ and C_17_ terpenes. The function of the cognate terpene synthase PgrE was evaluated by targeted point mutations within the observed Asp-rich motif D_92_DMPLG_97_ to map the effects of active-site residues on product formation. These investigations facilitated redirecting product selectivity, leading to alternative products. Overall, our work sheds light on the general biosynthetic logic leading to noncanonical C_17_ terpenes and provides a basis for their targeted discovery by genome mining and heterologous expression and for engineering C_17_ terpene structural frameworks.

## Graphical abstract

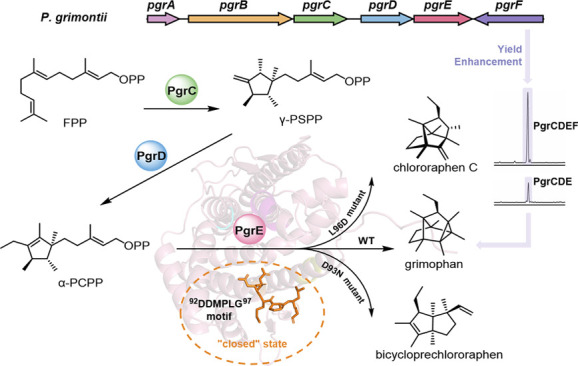

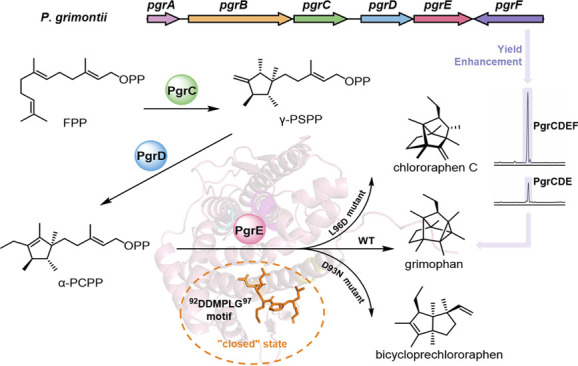

## Introduction

Terpenoids, small organic molecules characterized by highly diverse structural frameworks and widespread occurrence in nature,^1–4^ have attracted significant attention due to their biological activities and potential applications across pharmaceutical, agricultural, and industrial sectors.^5,6^ Generally, the main building blocks of terpenes are the C_5_ units isopentenyl pyrophosphate (IPP) and dimethylallyl pyrophosphate (DMAPP), which are typically provided by the 2-*C*-methyl-d-erythritol-4-phosphate (MEP) or the mevalonate (MVA) pathways.^7–9^ IPP and DMAPP are condensed by isopentenyl transferases to form extended terpenoid precursors, such as geranyl pyrophosphate (GPP, C_10_), farnesyl pyrophosphate (**1a**, FPP, C_15_), and geranylgeranyl pyrophosphate (GGPP, C_20_). These building blocks are subsequently further processed to give typical terpene structural frameworks, including monoterpenes,^10,11^ sesquiterpenes,^6,12,13^ diterpenes,^14,15^ and triterpenes.^16,17^ The intricate terpene structures are thereby assembled by cyclization and rearrangement reaction sequences guided by pathway-specific terpene synthases (TSs) (Figure 1).

**Figure 1:**
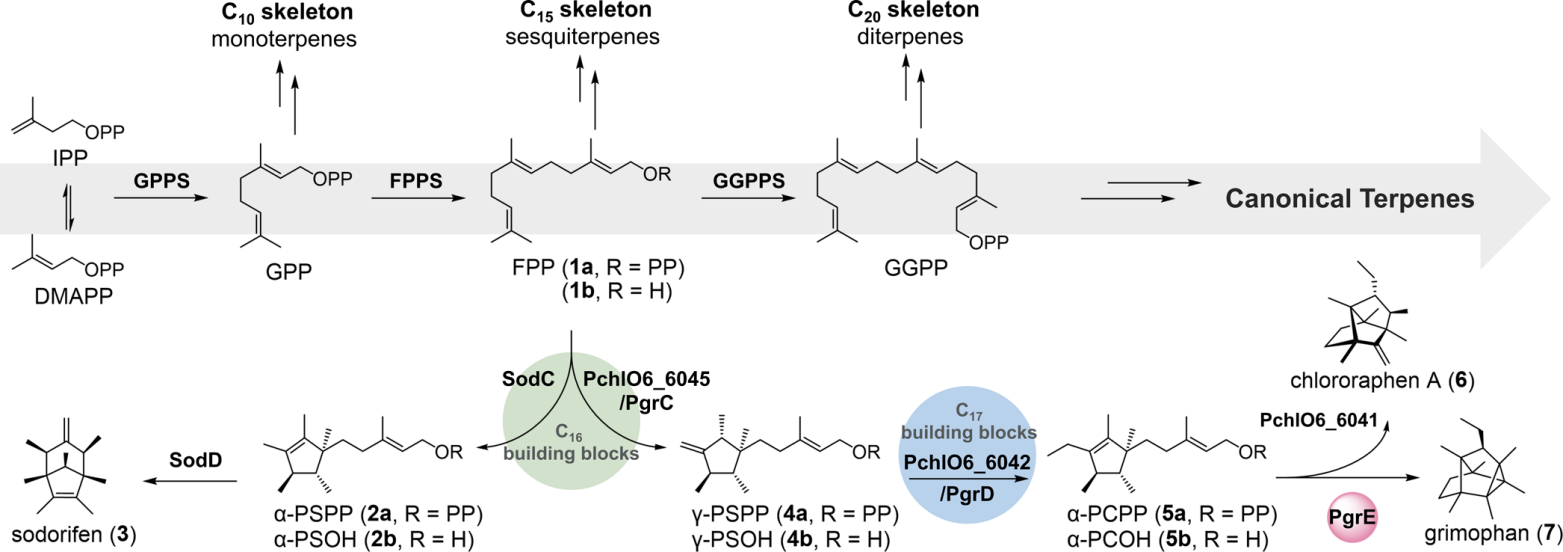
Schematic representation of terpenoid biosynthesis from precursors IPP and DMAPP. The upper section illustrates canonical terpene biosynthesis (gray arrow). Isoprenoid precursors IPP and DMAPP undergo condensation catalyzed by isopentenyl transferases to form extended linear precursors (GPP, FPP, GGPP, etc.), which are converted to monoterpenes, sesquiterpenes, and diterpenes, respectively. The bottom section depicts noncanonical terpene biosynthesis from modified FPP. FPP is initially methylated and cyclized to produce intermediates α-PSPP (**2a**, C_16_ terpenes) and α-PCPP (**5a**, C_17_ terpenes), which are further converted by TSs to generate sodorifen (**3**), chlororaphen A (**6**), and grimophan (**7**) identified in this study.

To date, only a very small number of atypical terpenoids (i.e., not based on multiples of C_5_ building blocks) derived from nonstandard carbon skeletons have been discovered in nature.^18–21^ While many terpenoid natural products exist that do not contain a carbon skeleton composed of *n* times C_5_, most of these can be traced back to the classical C_5_-derived precursors outlined above. Changes in the number of carbon atoms are rather a result of biosynthetic excision of parts of the IPP/DMAPP-derived carbon framework or late-stage addition of carbon building blocks during terpene biosynthesis. Examples include geosmin (C_12_), (*E*,*E*)-4,8,12-trimethyltrideca-1,3,7,11-tetraene (C_16_), and (*E*)-4,8-dimethyl-1,3,7-nonatriene (C_12_).^22–24^ Alternatively, two main strategies for the biosynthesis of atypical terpenoids by alterations to early precursor molecules exist. On the one hand, a modified MVA pathway can be utilized to directly generate C_6_ biosynthetic precursors, homo-IPP and homo-DMAPP, which are subsequently condensed to form atypical terpenoid skeletons. This mechanism is exemplified by the biosynthesis of juvenile hormones, sesquiterpenoid analogues found in *Lepidoptera* species.^25,26^ On the other hand, methyltransferases (MTs) that either methylate IPP/DMAPP or extended precursors such as GPP or FPP are known. Examples of terpenoids resulting from this biosynthetic logic include 2-methylisoborneol (2-MIB) (C_11_), sodorifen (**3**) (C_16_), and the unique chlororaphen A (**6**) (C_17_).^21,27–29^

In the biosynthesis of sodorifen-type noncanonical C_16_ terpenes, the MT SodC and its homologues possess both MT and cyclase activities. These enzymes are thus capable of methylating FPP to subsequently generate the common intermediate α-presodorifen pyrophosphate (α-PSPP, **2a**) by cyclization.^21,30,31^ This common intermediate **2a** can be further processed by TS SodD and its homologues to afford C_16_ terpenes with intriguing skeletons.^32,33^ Bioinformatic analyses revealed that *sodCD*-containing terpene BGCs are widely distributed in bacteria, with some BGCs containing more than one SodC-type MT/cyclase. Magnus et al. characterized the first product of such a pathway, found in *Pseudomonas chlororaphis* O6 and *Variovorax boronicumulans* PHE5–4, identifying the first terpene with a precursor-derived C_17_ skeleton, chlororaphen A (**6**).^28^ In the biosynthesis of **6**, FPP is initially monomethylated and cyclized by MT/cyclase PchlO6_6045 to form γ-presodorifen pyrophosphate (γ-PSPP, **4a**), a double-bond isomer of **2a**. The MT PchlO6_6042 subsequently introduces an additional methyl group into **4a** to yield α-prechlororaphen pyrophosphate (α-PCPP, **5a**). The latter is cyclized by TS PchlO6_6041 to produce the C_17_ terpene **6** (Figure 1).^28,33^ Bioinformatic screening of bacterial genomes revealed that over 70 BGCs putatively encoding such noncanonical terpenoids exist, suggesting considerable structural diversity of these terpenes awaiting discovery.^28^

Within this work, we discovered the novel C_17_ terpenoid grimophan (**7**)^34^ featuring a rare deltacyclane^35^ skeleton from *Pseudomonas grimontii* DSM 17515. Utilizing a heterologous expression strategy, we further (i) delineate its biosynthetic pathway through *in vitro* reconstitution, revealing TSs with high sequence identity exhibiting a differing catalytic mechanism; (ii) characterize a novel, biotechnologically valuable SAM-dependent MT class enhancing C_16_/C_17_ terpene production titers; and (iii) establish that residues within the D_92_DMPLG_97_ motif prototypical to the studied clade of C_17_ TSs from *Pseudomonas* sp. (a variation of the DDXX­(X)­D motif found in classical type I TSs) direct structural diversity by reshaping the enzyme active site cavity. Overall, our results provide strategies for the targeted discovery and expression of novel C_17_ terpenes and foster our understanding of the biosynthesis of FPP-derived atypical terpenoids. They also provide the first insights into approaches for further structural engineering by reshaping TS active-site composition.

## Results

### Genome Mining of FPP-Derived Noncanonical Terpene BGCs from *Pseudomonas* Strains

Previous studies have demonstrated that sodorifen-type noncanonical terpenes are widely distributed among various bacterial species, particularly within *Pseudomonas* sp.,^21,31^ suggesting that *Pseudomonas* sp. possess significant potential for the exploration of uncharacterized noncanonical terpenes. In *Pseudomonas*, several C_16_ terpenes, including anaximandrene, aristotelene, thaleene, and pseudorifen, were identified, along with the C_17_ terpene, chlororaphen A (**6**).^28,33^ However, a large number of BGCs potentially encoding novel noncanonical terpenes are awaiting exploration. Toward this goal, a search of the *Pseudomonas* sp. genome database (https://www.pseudomonas.com/) was performed, revealing a total of 121 annotated TSs and cyclase genes encoded across *Pseudomonas* sp. (Table S1). It is noteworthy that BGCs encoding FPP-derived nonclassical C_17_ terpenes likely require at least two MTs for extended intermediate formation. Apart from the only characterized pathway producing a C_17_ terpene, chlororaphene A (**6**),^28^ 11 additional BGCs fulfilling this precondition can bioinformatically be identified in *Pseudomonas* sp. (Figure 2A).

**Figure 2:**
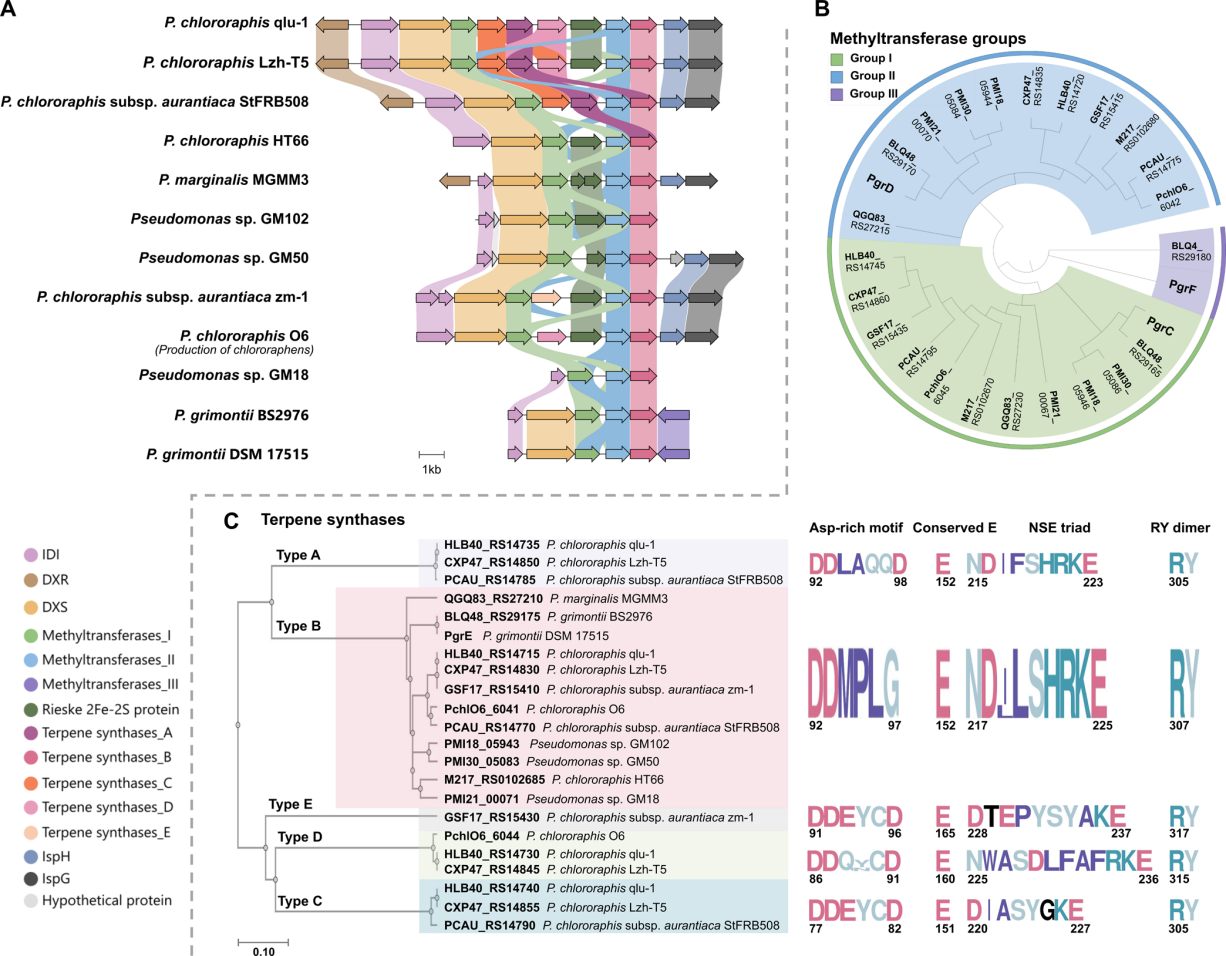
Analysis of BGCs putatively encoding FPP-derived C_17_ terpenes originating from *Pseudomonas* species. (A) Comparison of the 12 terpenoid BGCs containing at least two MTs. The diagram was created using the CAGECAT online analysis toolbox (Clinker).^36^ IDI: isopentenyl diphosphate delta isomerase; DXR: 1-deoxy-d-xylulose 5-phosphate reductoisomerase; DXS: 1-deoxy-d-xylulose 5-phosphate synthase; IspH: 4-hydroxy-3-methylbut-2-enyl diphosphate reductase; IspG: 4-hydroxy-3-methylbut-2-en-1-yl diphosphate synthase. (B) Phylogenetic tree of MTs located within the 12 BGCs grouped into three (I–III) based on homology, visualized using iTOL^37^ (https://itol.embl.de/). Protein sequence alignment and phylogenetic reconstruction were conducted using ETE3 (3.1.3)^38^ as implemented on the GenomeNet. (C) Phylogenetic tree of TSs from the 12 BGCs grouped into five (A–E), including catalytically crucial conserved motifs (right) derived from sequence alignment. Sequence logos created using WebLogo.^39^

Based on protein sequence alignment and phylogenetic analysis, the MTs within these 12 BGCs can be divided into three groups (Figure 2B, Table S2), while the TSs can be classified into five groups (Figure 2C, Table S3). All BGCs potentially producing C_17_ terpenes contain at least one group I MT, one group II MT, and one group B TS, with some BGCs featuring multiple TSs. TSs within the same group exhibit high sequence identity (Figures S1–S5), while those from different groups show low identity. A homologue of the TS PchlO6_6041 (enzymes named PchlO6_604X are all involved in production of chlororaphen) belonging to group B is encoded in all 12 BGCs. The corresponding enzymes share over 86% amino acid sequence identity with each other while displaying only 20–22% identity compared to the TS SodD from sodorifen biosynthesis (Figure S2). Notably, in contrast to groups A, C, D, and E, which contain the conserved Asp-rich motif DDXX­(X)­D of typical type I TSs, a hallmark motif involved in divalent metal ion binding for substrate activation, group B TSs all possess a unique Asp-rich motif ^92^DDMPLG^97^(Figure 2C). For group I MTs, PchlO6_6045 and its homologous proteins exhibit over 80% sequence identity with each other at the amino acid level and 57–61% sequence identity with SodC, which catalyzes the formation of α-PSPP (**2a**) from FPP (Figure 1, Figure S6). In the case of group II MTs, PchlO6_6042 shares up to 90% identity with its homologues, while sequence identity with SodC is approximately 45% (Figure S7). It is important to note that group III MTs can solely be identified in *P. grimontii* DSM 17515 and *P. grimontii* BS2976 and show low identity with both group I and II MTs and to SodC (Figure S8), suggesting a potentially distinct methylation activity. Given the presence of three different MTs in *P. grimontii*, we hypothesized that this BGC might produce a novel noncanonical terpene, potentially with a C_18_ backbone. To elucidate how methylation diversity contributes to precursor diversity and subsequently to terpene structural diversity, the *pgr* BGC from *P. grimontii* DSM entry 17515 was thus selected for further study (Table S4).

### BGC *pgr* from *Pseudomonas grimontii* Encodes a Novel Noncanonical C_17_ Terpene, Grimophan

Our previous studies demonstrated that *E. coli* serves as an ideal host for the heterologous expression of terpene BGCs originating from *Pseudomonas* sp.^40–42^ Consequently, we aimed at the recombinant expression of the *pgr* BGC from *P. grimontii* DSM 17515 in *E. coli* BAP1 (Figure 3A). Within *pgr*, the genes *pgrABCDE* are located on a single operon. They encode proteins putatively involved in IPP precursor biosynthesis (PgrA: IDI = isopentenyl diphosphate delta isomerase; PgrB: DXS = 1-deoxy-*D*-xylulose 5-phosphate synthase), a group I (PgrC) and a group II (PgrD) MT, and a type B TS (PgrE) (Table S4). In addition, group III MT PgrF is encoded downstream of *pgrABCDE*. Initially, the entire *pgrABCDE* operon was cloned into the pET28-ptetO-gfpv2 vector using the DiPaC strategy,^40,43–46^ followed by heterologous expression in *E. coli* BAP1. The expression strain *E. coli* BAP1::*pgrABCDE* was cultivated on a scale of 3 L in TB medium at 20 °C. Volatile organic compounds (VOCs) were intercepted on a charcoal filter at the gas outlet of the applied fermenter, followed by elution of bound VOCs using pentane. The resulting solution was analyzed by GC-MS. Compared to *E. coli* BAP1 carrying the empty vector (Figure 3B, i), *E. coli* harboring the *pgrABCDE* operon produced compound **7** with an *m*/*z* of 232 [M]^•+^ and a retention time of 14.2 min as the major component (ii). Removal of the genes *pgrAB* likely involved in precursor biosynthesis and heterologous expression of *pgrCDE* alone proved sufficient to produce **7** at similar production titers (iii, Figure S9). Alternative expression of either *pgrCE* or *pgrDE* alone, however, failed to yield **7** (Figure S9), attesting to the necessity of both MTs being present in the expression construct. We next added the group III MT-encoding gene *pgrF* to the expression construct, resulting in plasmid pET28-ptetO-*pgrCDEF*, to investigate whether this MT further modifies **7**. Expression in *E. coli* BAP1 revealed that the addition of *pgrF* significantly enhanced the titer of **7**, but no additional product was formed (iv). To test whether **7** is also produced by the native host, *P. grimontii* DSM 17515, we also analyzed the VOCs produced by this organism. GC-MS analysis showed the presence of **7**, albeit in low abundance (v, Figure S9), evidencing that recombinant and native expression of the *pgr* BGC led to the identical product.

**Figure 3:**
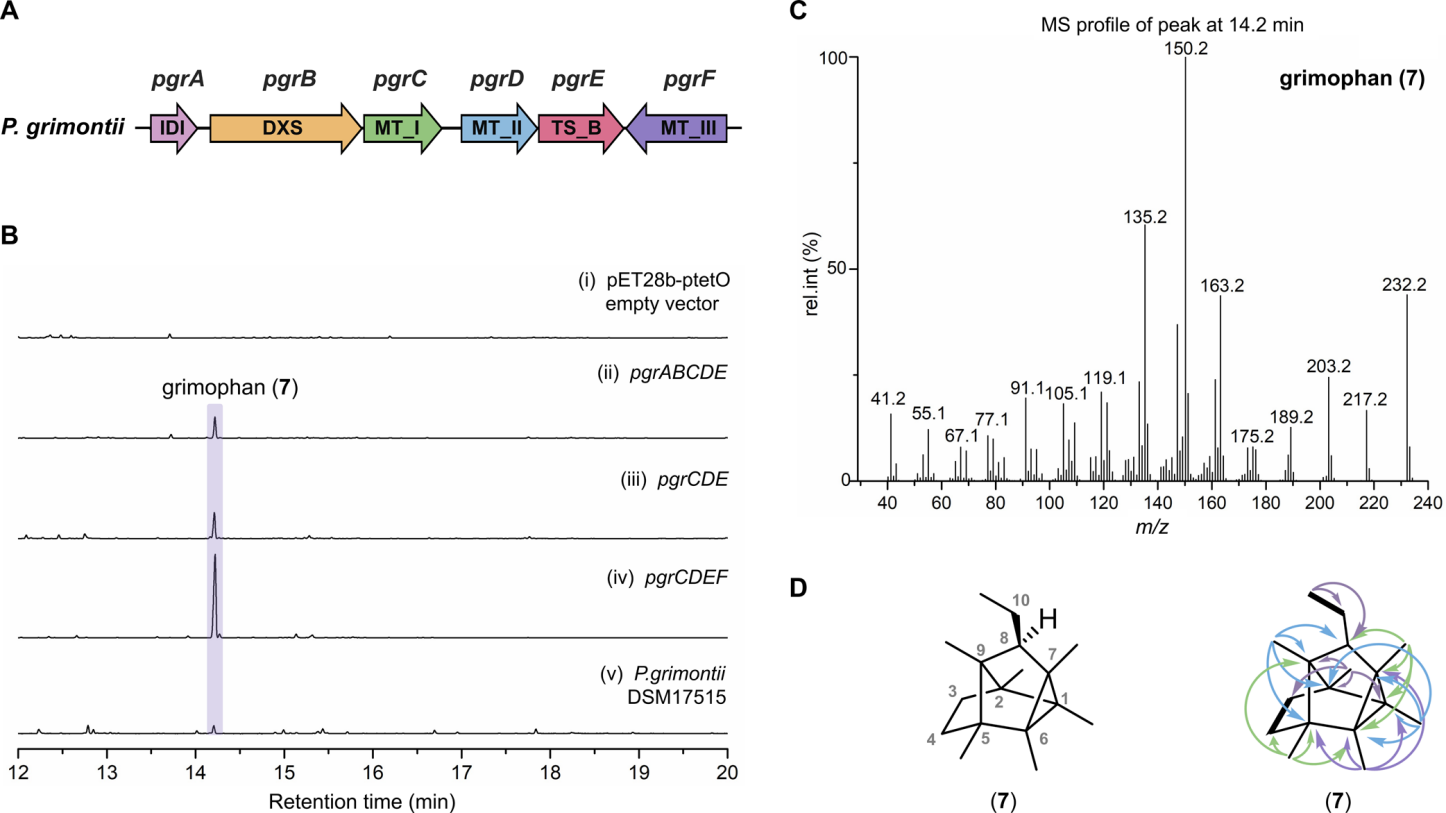
Identification of grimophan (**7**) through the heterologous expression of the *pgr* cluster in *E. coli* BAP1. (A) Genetic organization of the *pgr* cluster from *Pseudomonas grimontii* DSM 17515. (B) GC-EI-MS analysis of VOCs produced by *E. coli* BAP1 strains harboring (i) empty vector pET28b-ptetO-GFPv2 or vector containing (ii) *pgrABCDE*, (iii) *pgrCDE*, (iv) *pgrCDEF*, and (v) *P. grimontii* DSM 17515. The identity of **7** across all expression experiments was unambiguously validated through the comparison of GC-MS fragmentation patterns (Figure S9). (C) GC-EI-MS spectrum of grimophan (**7**). (D) Structure of grimophan (**7**) (left) along with selected COSY and HMBC correlations used for structure elucidation (right; cf. Figure S27–S41 for details). Bold bonds: ^1^H,^1^H–COSY interactions; single-headed arrows: key HMBC correlations.

Although the proteins encoded in the *pgr* BGC possess highly conserved sequences compared to their homologues involved in the biosynthesis of chlororaphen (**6**), the observed product **7** has a distinct structure based on its GC-MS fingerprint (Figure 3C). Detailed analysis using LC-APCI-HRMS revealed **7** to have an *m*/*z* of 232.2177 [M]^•+^, leading to a putative molecular formula of C_17_H_28_ (calcd. *m*/*z* 232.2186 [M]^•+^). Dominant fragment ions from GC-EI-MS (70 eV) at *m*/*z* 150, 135, and 163 (Figure 3C) distinguished the MS data from those of chlororaphen A (**6**) and B.^28,33^ To enable full structure elucidation, **7** was heterologously produced from *E. coli* BAP1::*pgrCDE*F, purified by column chromatography, and submitted to NMR spectroscopy, with full data sets recorded in CDCl_3_ and C_6_D_6_ (Figures S28–S41 and Table S8). ^1^H NMR data of **7** showed the presence of six singlets assigned to methyl groups (δ_H_ = 0.68 [Me-9], 0.73 [Me-2], 0.78 [Me-1], 0.80 [Me-6], 0.84 [Me-5], and 0.99 [Me-7] ppm in C_6_D_6_), two CH_2_ groups directly connected to each other (δ_H_ = 1.17, 1.45 [CH_2_–3], and 1.18, 1.42 [CH_2_–4]), and a CH group (δ_H_ = 1.22 [CH-8]) connected to an ethyl group (δ_H_ = 1.32, 1.40 [CH_2_–10], 0.98 [Me-10]), but no signals for proton-bearing double bonds (Figure 3D). ^13^C NMR spectra were in agreement with this assignment and revealed the additional presence of six quaternary carbons (cf. Table S8). The connectivity of all carbons in **7** was readily accessible by the prototypical ^2^ *J*_CH_ and ^3^ *J*_CH_ HMBC correlations of all methyl groups (Figure 3D, Figure S27). Overall, **7** possesses a rigid tetracyclic skeleton with a stereogenic center at the ethyl-substituted C-8 position. The relative configuration at C-8 was deduced as depicted in Figure 3D by nuclear Overhauser enhancement spectroscopy (NOESY), which clearly showed a correlation between H-8 and Me-2, but not between H-8 and Me-5. The overall structure of **7** represents a unique deltacyclane skeleton, containing a 9-membered ring as the largest cyclic structure.

### Functional Characterization of the Roles of All Enzymes Involved in Grimophan (**7**) Biosynthesis

The three genes encoding MTs (*pgrC*, *pg*r*D*, and *pgrF*) along with the TS-encoding gene *pgrE* from the *pgr* BGC were individually cloned into the pHis8-TEV vector using sequence- and ligation-independent cloning (SLIC).^47^ The resulting expression vectors were transferred to *E. coli* BL21­(DE3). Protein expression was carried out in LB medium on a 1 L scale, with induction of production at an OD_600_ of 0.6–0.8 by the addition of IPTG at a final concentration of 0.1 mM. Cells were harvested by centrifugation and lysed by sonication. The soluble proteins in the resulting supernatant were further purified by chromatography on Ni-NTA beads (Figure S10).

Systematic enzyme assays with FPP (**1a**) and *S*-adenosylmethionine (SAM) as substrates were conducted to investigate the roles of all enzymes involved in the biosynthesis of **7** (Figure 4A). All detected compounds were unambiguously identified by analysis of their GC-MS fingerprints (Figure 4B) and comparison to reference data (Figures S12, S42–S50, S62).^28,48,49^ *In vitro* assays with the full set of enzymes (PgrC-F) shown to be required for formation of **7** *in vivo* led to the expected efficient formation of **7** (Figure 4A, i). Omitting the titer-enhancing PgrF resulted in significantly reduced product amounts (ii). To test the order of catalytic action of the individual enzymes, enzyme combinations of MTs/TS that appeared meaningful and informative were tested. To enable detection of pyrophosphate-bearing intermediates by GC-MS analysis, all assays were treated with alkaline phosphatase (AP) at the end of the incubation time to hydrolyze pyrophosphates, followed by extraction with hexane. When the assay solution was solely treated with AP, the expected exclusive formation of (2*E*,6*E*)-farnesol (**1b**, retention time (RT) 17.9 min, *m*/*z* 222 [M]^•+^) was observed, which is the direct product of pyrophosphate cleavage from substrate **1a** (iii) (Figures S12 and S62). The addition of PgrD alone did not alter the product spectrum (iv), indicating that this enzyme does not act on **1a**. When assays were performed with PgrC alone (v), the C_16_ terpene γ-presodorifenol (**4b**, RT 18.4 min; C_16_H_28_O, calcd. *m*/*z* 236.2135 [M]^•+^, obsd. *m*/*z* 236.2139 [M]^•+^) was additionally produced, attesting to the dual function of this enzyme as an MT/TS. Additional inclusion of PgrD (vi) revealed the formation of the C_17_ precursor α-prechlororaphenol (**5b**, RT 18.6 min; C_17_H_30_O, calcd. *m*/*z* 250.2291 [M]^•+^, obsd. *m*/*z* 250.2295 [M]^•+^). This reveals the MT activity of PgrD to install the second required methyl group for the biosynthesis of **7**.

**Figure 4:**
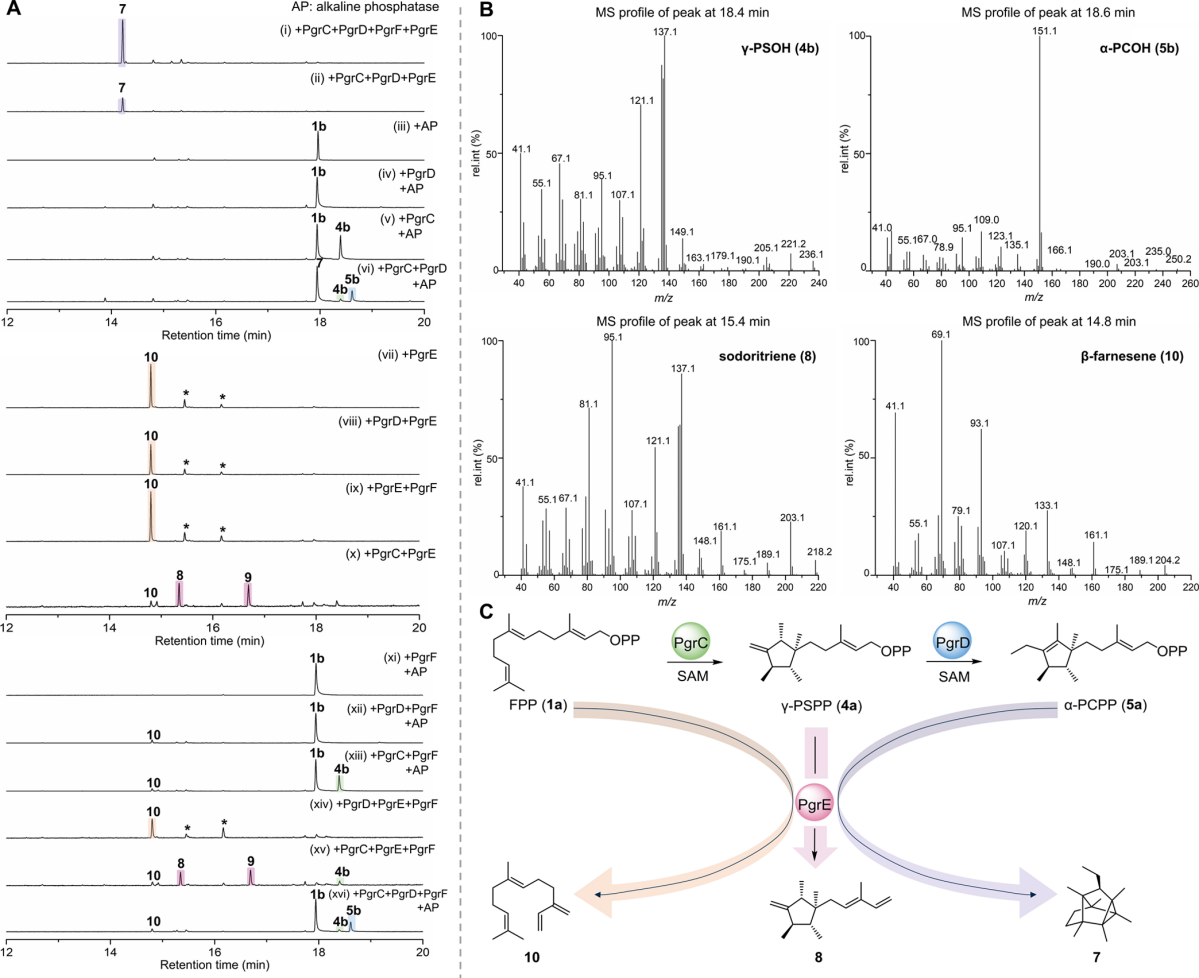
Elucidation of the biosynthetic pathway of grimophan (**7**). (A) GC-EI-MS analysis of VOCs produced during *in vitro* assays. All assays were performed using FPP (**1a**) and SAM as substrates. The signal intensity for **7** in the assay (i) was set to 100%, to which all other chromatograms were normalized. Peaks labeled with an asterisk indicate uncharacterized VOCs. (B) GC-EI-MS profiles of γ-PSOH (**4b**), α-PCOH (**5b**), sodoritriene (**8**), and β-farnesene (**10**). The identity of compounds **4b**, **5b**, **7**, **8**, **9**, and **10** across all experiments was unambiguously proven by comparison of GC-MS fragmentation patterns and retention times. (C) The biosynthetic pathway to **7** and to further enzymatic products **8** and **10**.

Assays that were conducted with the TS PgrE alone led to the formation of compound **10** (RT = 14.8 min; C_15_H_24_, calcd. *m*/*z* 204.1873 [M]^•+^, obsd. *m*/*z* 204.1880 [M]^•+^) (Figure 4A, vii). This was also the case when assays with PgrE were individually supplemented with MTs PgrD (viii) or PgrF (ix). Isolation and NMR analyses determined this product to be β-farnesene (**10**)^49^ (Figure 4B, Figures S48–S50). In the absence of its cognate substrate, PgrE thus catalyzes the elimination of the pyrophosphate group to generate a terminal conjugated double bond system, similar to a promiscuous TS Bcl-TS identified from *Bacillus clausii* ^50–52^ and TS PcchC involved in aristotelene biosynthesis.^21^ A similar reactivity of PgrE was observed in assays together with PgrC (x). This led to products **8** (RT = 15.4 min; C_16_H_26_, calcd. *m*/*z* 218.2029 [M]^•+^, obsd. *m*/*z* = 218.2034 [M]^•+^) and **9** (RT = 16.7 min, *m*/*z* = 236 [M]^•+^) (Figure S11). Isolation and NMR structure elucidation showed **8** to be the new compound sodoritriene, resulting from the initial formation of **4a** by PgrC and the subsequent elimination of the pyrophosphate group to likewise give a terminal double bond (Figure 4B,C and Figures S42–S47, Table S9).

Heterologous expression of *pgrE* or *pgrCE* introduced into pET28-ptetO-GFPv2-based plasmids yielded results consistent with those of the corresponding *in vitro* enzymatic assays (Figure S9). These findings raised questions regarding the timing of FPP modification catalyzed by PgrC and PgrE in grimophan (**7**) biosynthesis. To rule out the possibility that PgrE initially catalyzes the conversion of **1a** to β-farnesene (**10**) with subsequent methylation and cyclization catalyzed by PgrC to afford **8**, we conducted additional assays using **10** as the substrate. Compound **10** was isolated from assays with PgrE alone (Figure 4A, vii) and directly subjected to an individual enzyme assay with PgrC in the presence of SAM. GC-EI-MS results clearly showed that **10** does not serve as a substrate to PgrC and hence is not involved as an intermediate in the biosynthesis of **7** (Figure S13). Based on these combined results, the biosynthesis of **7** can be depicted as follows: PgrC initiates the methylation and cyclization reactions of **1a** (C_15_) to produce intermediate γ-PSPP (**4a**, C_16_), which can then be further methylated by PgrD to form α-PCPP (**5a**, C_17_), with final cyclization of **5a** by PgrE to generate grimophan (**7**) (Figure 4C).

Given that PgrC, PgrD, and PgrE are sufficient for the biosynthesis of grimophan (**7**), we hypothesized that PgrF may functionally substitute for one of the MTs, e.g., analogous to auxiliary MTs compensating for other *O*-MTs in xantholipin biosynthesis.^53^ Assays with **1a**, SAM, and PgrF alone with treatment of AP did result only in formation of the AP-hydrolyzed product **1b** (Figure 4A, xi). Supplementation of assays containing either PgrD (xii) or PgrC (xiii) with PgrF gave identical results as the corresponding assays without PgrF (iv and v, respectively). Furthermore, the product spectrum of assays with PgrDE, PgrCE, or PgrCD in the presence of PgrF (xiv, xv, xvi) did also not change when compared to those devoid of PgrF (viii, x, vi). Based on these results, PgrF seems to indeed exclusively impact the final production yields of **7**, raising questions about the underlying molecular mechanism.

### The Functional Role of PgrF in the Biosynthesis of Grimophan (**7**)

MTs typically adopt homodimeric states in their catalytically active forms, with only a limited subset of MTs shown to be capable of forming heterodimers to modulate catalytic efficiency.^54–56^ To investigate potential heterodimerization in the case of PgrC/D and PgrF, computational analyses using the GalaxyHeteromer computation tool^57,58^ were conducted to predict potential intermolecular interactions between PgrC and PgrF, as well as PgrD and PgrF through template-based homology modeling and *ab initio* docking. The results revealed extensive hydrogen bond interactions at the protein–protein interface of both, PgrC and D, with PgrF. This involves a series of amino acid residues such as aspartic acid, arginine, lysine, and glutamic acid (Figure 5A,C). Furthermore, GST pull-down assays were employed to experimentally validate potential protein–protein interactions. PgrF was expressed as a GST-tagged protein, while PgrC and PgrD were expressed as His_8_-tagged proteins. Co-purification experiments were performed using GST affinity chromatography, and the eluted protein fractions were analyzed by sodium dodecyl sulfate polyacrylamide gel electrophoresis (SDS-PAGE). Compared to the control experiments, the GST pull-down assays clearly indicated copurification of GST-PgrF with either PgrC (Figure 5B) or PgrD (Figure 5D), indicating significant protein–protein interactions between these respective protein pairs. These results nicely corroborate the expectations from the GalaxyHeteromer predictions. Although protein–protein interactions were observed between both, PgrC/PgrF and PgrD/PgrF, the results of the *in vitro* enzymatic assays (Figure 4A-(xii) to (xvi)) show that the addition of PgrF to assays with PgrC or PgrCD only results in a modest increase (approximately 1.5- to 1.6-fold) of the production titers of intermediates **4** and **5**, respectively. However, addition of PgrF to assays containing PgrCDE leads to a substantially increased yield (approximately 4.1-fold) of the final, pathway-specific product **7** (Figure 5E). Taken together, these results strongly suggest that PgrCDEF function through coordinated interactions within a higher-order protein complex, which in concert leads to optimized interactions and likely improved precursor funneling that contributes to the overall strongly improved catalytic efficiency in grimophan (**7**) biosynthesis, rather than PgrF only improving the methylation efficiency of PgrCD in the production of precursor **4** and **5**.

**Figure 5:**
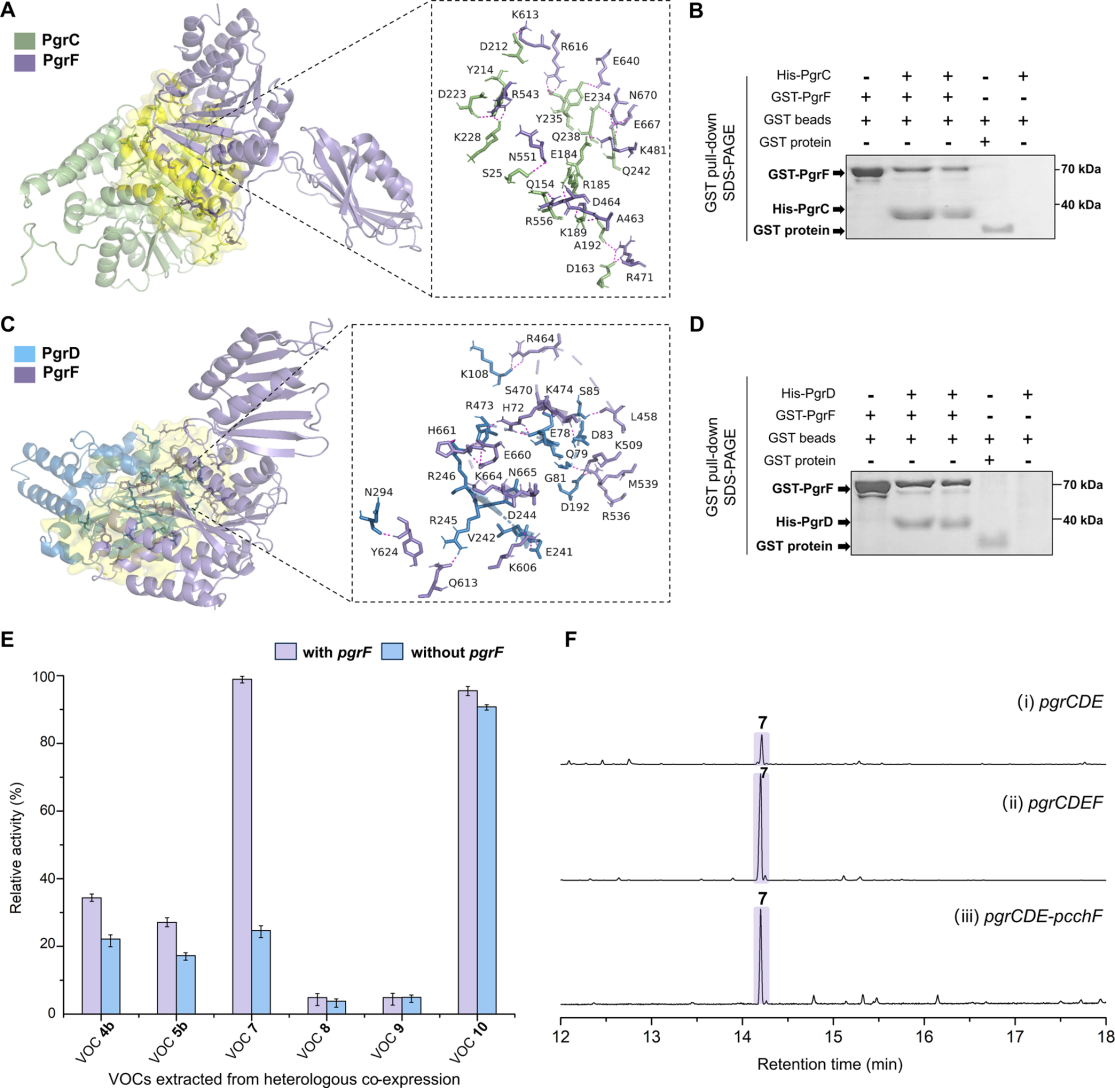
Functional characterization of MT PgrF. (A, C) Protein–protein interaction interfaces of PgrC (A; chain A; residues 1-313) with PgrF (A; chain B; residues 314-718), and PgrD (C; chain A; residues 1-306) with PgrF (C; chain B; residues 307-711) obtained by GalaxyHeteromer-based protein sequence modeling and docking.^57,58^ The interaction interface is highlighted in yellow. The magnified section shows specific intermolecular interactions of the amino acid residues (shown in the stick model) at the protein–protein interface. The visualization was created by PyMOL.^59^ (B, D) GST pull down assays and SDS-PAGE analysis of the interaction of PgrC/D (His tag) with PgrF (GST tag). (+) indicates the presence and (−) the absence of a component in each lane. Expected protein sizes: His8-PgrC (36 kDa), His8-PgrD (35 kDa), GST-PgrF (71 kDa), and GST protein (28 kDa). The complete SDS-PAGE analysis is depicted in Figure S14. (E) Relative yields of VOCs obtained by heterologous coexpression of genes encoded within *pgr* with/without *pgrF* in *E. coli*. VOC **4b** was obtained from *pgrC*(*F*), VOC **5b** was obtained from *pgrCD*(*F*), VOC **7** was obtained from *pgrCDE*(*F*), VOCs **8** and **9** were obtained from *pgrCE*(*F*), and VOC **10** was obtained from *pgrE*(*F*). The relative yield of compound **7** produced by the recombinant expression of *pgrCDEF* was set to 100%. The relative yields were compared using average values (±SD, *n* = 3). (F) Comparison of GC-EI-MS total ion chromatograms with/without the addition of *pgrF*-type MTs during the heterologous expression of compound **7**. PcchF from *P. chlororaphis* subsp. *chlororaphis* DSM 50083.

Targeted bioinformatic screening for further PgrF-type group III MTs (Figure 2B) using PgrF as a probe showed that homologous enzymes with high sequence identity are widely distributed among *Pseudomonas* sp. (for selected examples, see Table S5, Figures S15–S17). Interestingly, we also identified highly homologous proteins in *Pseudomonas chlororaphis* subsp. *chlororaphis* DSM50083 and in *Pseudomonas chlororaphis* subsp. *aureofaciens* DSM6698, both known producers of C_16_ noncanonical terpenes.^21^ Therefore, it was of high interest to test if yield-enhancing effects can generally be achieved by employing such enzymes. As an example, we selected the homologous MT C4K27_RS06760 (87% identity to PgrF) from *P. chlororaphis* subsp. *chlororaphis* DSM 50083 (Table S5, Figure S15), the producer of the C_16_ terpene aristotelene.^21^ The encoding gene (herein termed *pcchF*) was assembled into pET28-ptetO-gfpv2-based expression vectors pET28-ptetO-gfpv2::*pgrCDE-pcchF* and pET28-ptetO-gfpv2::*pcchBCF*, downstream of *pgrCDE* (for production of **7**) or *pcchBC* (for production of aristotolene). Heterologous expression in *E. coli* BAP1 and *in vitro* reconstitution indeed proved that both, the yield of **7** or of aristotelene, significantly increased compared to the expression constructs devoid of PcchF (Figure 5F and Figure S18–S20). These findings show that PgrF and PcchF enhance the efficiency of noncanonical terpene biosynthesis in *Pseudomonas* sp. even across pathways.

To understand the genomic context of PgrF-like enzymes in *Pseudomonas* sp., we analyzed the genetic neighborhood of group III MT homologues using global EFI-GNT analysis (Figure S17). Notably, with the exception of *P. grimontii* DSM 17515, *pgrF* homologues reside in conserved genomic regions and are flanked by genes unrelated to terpenoid biosynthesis, such as genes encoding membrane proteins and transporters. The genomic architecture in *P. grimontii* thus represents an exception: the core *pgrABCDE* BGC appears to have been inserted immediately upstream of a *pgrF* gene into the conserved genomic context found in the other strains (Figure S17, Table S5). This strongly suggests a horizontal gene transfer (HGT) event that inserted the terpenoid biosynthetic machinery into the locus already containing a *pgrF* homologue.

### Production of FPP-Derived C_17_ Terpenes by Engineering PgrE

In general, type I TSs employ a DDXX­(X)­D motif coordinating Mg^2+^ and an NSE triad to ionize substrates by pyrophosphate abstraction, generating allylic carbocations for cyclization.^60^ However, PgrE and its homologous proteins in TS group B contain an atypical Asp-rich motif with a conserved DDMPLG site. Although PgrE shares 88.5% sequence identity with its homologous protein PchlO6_6041 in the group, their enzymatic products differ significantly, with PgrE catalyzing the production of **7** and Pch106_6041 catalyzing the production of **6** from the identical precursor **5a**. This functional divergence raises questions regarding the molecular mechanisms and crucial active-site residues of these two enzymes. To obtain first mechanistic insights, the protein structures of PgrE, PchlO6_6041, and ten other TS homologues were predicted using AlphaFold3,^61^ followed by structural alignment (Figure S24). Notably, the amino acid residues of the conserved atypical D^92^DMPLG^97^ motif in PgrE have a distinct spatial arrangement compared to the other 11 TSs in group B, despite their high overall sequence identity and identical motifs. As illustrated in Figure 6A, the spatial arrangement of the amino acid residues located in the conserved motif indicates that PchlO6_6041 adopts an “open” state, whereas PgrE is in a “closed” state. This variation is primarily attributed to the positioning of the amino acid residues P^95^LG^97^. Given the crucial role of this motif in substrate recognition/binding and metal ion coordination, we further explored whether the observed product differences can be linked to these differences in spatial arrangement within the active site.

**Figure 6:**
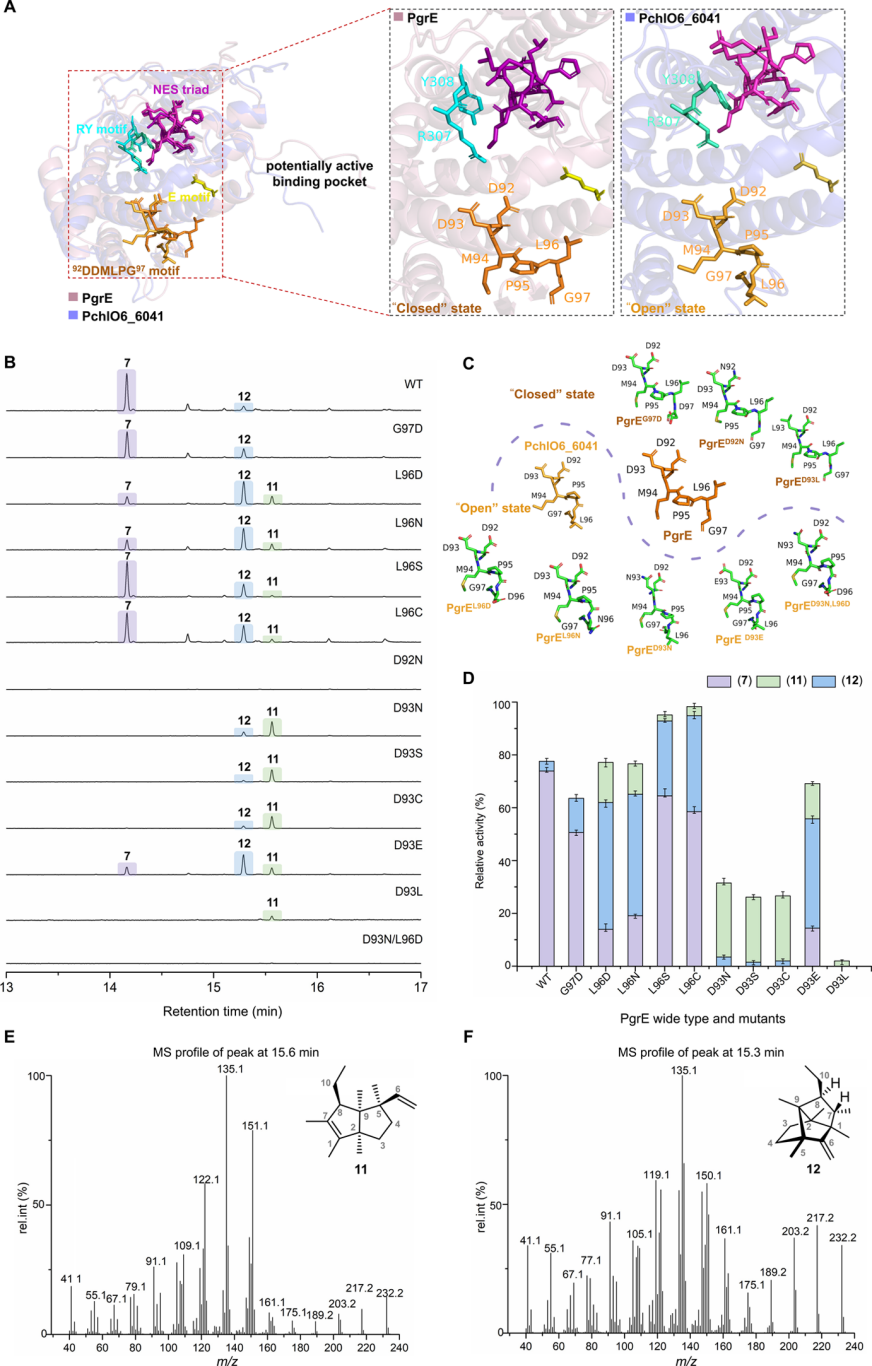
Analysis of the structure and catalytic activity of PgrE and its variants. (A) Alignment of the structure of PgrE and its homologue PchlO6_6041 predicted by AlphaFold3 ^61^ (Figures S21–S22). The protein structure is shown in a cartoon model, while the amino acid residues of the conserved sequence are shown in a stick model. The potential active binding pocket is partially magnified to highlight the spatial conformation within the amino acid residues of the conserved motif, especially the Asp-rich motif. (B) Comparative GC-EI-MS analysis of VOCs produced using a combination of PgrC, PgrD, and PgrE or PgrE mutants with FPP and SAM as substrates. The relative yield of compound **7** produced by wild-type PgrE was set to 100%. (C) The spatial configuration of the amino acid residues of the conserved D^92^DMLPG^97^ motif following alignment of the protein structures of wild-type PgrE (brown) and its eight selected mutants. The predicted protein structures of all PgrE mutants are shown in Figure S24. (D) The relative catalytic activities of wild-type and PgrE mutants in converting precursor FPP to compounds **7**, **11**, and **12** in *in vitro* enzyme assays. The relative yields were compared using average values (±SD, *n* = 3). (E, F) The structures and GC-EI-MS fingerprints of compounds **11** (E) and **12** (F).

Reinspection of the product profile of enzyme assays conducted with WT PgrE (Figure 4A-i) showed high-level production of **7** along with very low production levels of an additional product **12** (RT 15.3 min; C_17_H_28_, obsd. *m*/*z* 232.2193 [M]^•+^; Figure S11; for structure elucidation of **12**, see below). When the conserved motif D^92^D^93^MP**L** ^**96**^ **G** ^**97**^ of PgrE was altered to D^92^D^93^MPL^96^ **D** ^**97**^ or D^92^D^93^MP**D** ^**96**^G^97^, resembling the typical conserved motif DDXX­(X)­D found in type I TSs, we observed in the predicted protein structures that the mutated protein PgrE^G97D^ retained a conformation akin to PgrE, while PgrE^L96D^ exhibited a conformation similar to PchlO6_6041 (Figure 6C). Enzymatic assays demonstrated that PgrE^G97D^ produced a metabolite profile comparable to that of wild type PgrE (Figure 6B). However, the product profile generated by PgrE^L96D^ changed significantly, with a dramatic decrease in **7**, accompanied by a significant increase in **12** and formation of the new compound **11** (RT = 15.6 min; C_17_H_28_, obsd. *m*/*z* 232.2193 [M]^•+^) at equal amounts compared to that of **7** (Figure 6B). This finding strongly indicated that in the non-native “open” state of the PgrE mutant enzyme, the ability to tightly bind and hence efficiently process the substrate is reduced, thereby promoting the generation of diverse C_17_ terpenoid side products. Next, we investigated whether substituting L^96^ with other hydrophobic amino acids, such as N^96^, S^96^, and C^96^, affects the product spectrum. The mutated proteins PgrE^L96N^, PgrE^L96S^, and PgrE^L96C^ were predicted by AlphaFold3 to exhibit open-state structures similar to that of PgrE^L96D^ (Figure 6C, Figure S25), although their product distribution varied. PgrE^L96N^ displayed a product profile identical to that of PgrE^L96D^, while those of PgrE^L96S^ and PgrE^L96C^ were akin to wild type PgrE, with the generation of **7** remaining largely unaffected, along with a slight increase in production of **12** and trace amounts of **11** (Figure 6B). These findings suggest that amino acids at this position may influence the product distribution through further interactions with substrate binding in the hydrogen bond network. Taken together, we hypothesize that both the conformation of the conserved motif D^92^D^93^MPL^96^G^97^ and the type of amino acid present at position 96 are critical for the selectivity (or lack thereof) of product formation.

As the aspartate residues within the Asp-rich motif of bacterial type I TSs are essential for metal ion coordination, which initiates substrate binding and subsequent catalysis,^60^ we next investigated the roles of both Asp residues within motif D^92^D^93^MPL^96^G^97^ by additional site-directed mutagenesis. The PgrE^D92N^ mutant exhibited a complete loss of catalytic activity (Figure 6B). Given that the first Asp residue of the conserved motif typically coordinates Mg^2+^ ions,^60^ mutation at this position would therefore be expected to disrupt this critical metal ion coordination, leading to the observed loss of activity. Notably, based on protein structures predicted by AlphaFold3, the substitution of D^92^ with N^92^ did not result in a significant alteration of the “closed” conformation of the conserved sequence PgrE^D92N^ (Figure 6C). However, mutation of D^93^ to N^93^ resulted in a significant change in the AlphaFold3-predicted spatial arrangement of the Asp-rich motif (Figure 6C), transitioning from a “closed” state to an “open” state. Enzymatic assays demonstrated that the PgrE^D93N^ mutant completely lost the ability to produce **7**, with formation of **11** as the main product and **12** as the minor product. Considering these findings, we further investigated whether the product profile would change upon mutation of D^93^ to amino acids with other hydrophilic side chains. D^93^ was consequently mutated to S, C, and E, and the activities of these mutant proteins were assessed. Despite PgrE^D93S^, PgrE^D93C^, and PgrE^D93E^ exhibiting a similar “open” state to PgrE^D93N^ (Figure 6C, Figure S25), they displayed distinct product profiles. PgrE^D93S^ and PgrE^D93C^ demonstrated similar product profiles to PgrE^D93N^, whereas PgrE^D93E^ exhibited a similar product profile to PgrE^L96D^ and PgrE^L96N^ with the formation of small amounts of **7** (Figure 6B). Additionally, we established the PgrE^D93L^ mutation to mimic the steric hindrance of PgrE and lock the conformation in a “closed” state (Figure 6C). Enzyme assays (Figure 6B) revealed a drastic reduction in catalytic activity with only trace amounts of **11** formed. This loss of function is presumably due to the hydrophobic methyl group of L^93^, which disrupts the critical electrostatic interaction mediated by D^93^ within the binding pocket. Overall, these findings suggest that the flexibility of the amino acid residues at position 93 is crucial for the efficient assembly of C_17_ terpenoids. Based on the results of the single-site mutagenesis, we proceeded to create a double mutant, PgrE^D93N,L96D^, incorporating mutations at the active sites D^93^ and L^96^. The corresponding double mutant completely lost its catalytic activity (Figure 6B). Although the predicted protein structure indicated that the PgrE^D93NL96D^ protein displayed steric hindrance similar to that of D^93^N or L^96^D, the conformation of the RY dimer changed significantly, positioning outside the anticipated active site pocket (Figure 6C, Figure S25), explaining the loss of activity.

To enable the production of sufficient amounts of **11** and **12** for isolation and structure elucidation, the D93N mutation was introduced into *pgrF* present in the plasmid pET28-ptetO-gfpv2::*pgrCDEF*, which was subsequently heterologously expressed in *E. coli* BAP1. The production was conducted in 3 L of TB medium for 5 days. Compounds **11** and **12** were then isolated by column chromatography after absorption on charcoal, as described for compound **7**. While separation of the two terpenoids was not possible due to their similar chromatographic properties and volatility, their structures were discerned by NMR analysis of the mixture. Compound **12** (Figure S56–S61, Table S11) was identified as a diastereomer of chlororaphen A (**6**) and hence termed chlororaphen C (**12**). Key distinctions in stereochemistry were established by NOESY correlations between CH-8, Me-1, Me-2, and Me-7, and of CH-7 with Me-5 and Me-9, which also explained the differing chemical shifts of the alkene carbons when compared to those in chlororaphen A (**6**) (δ_C_ (C6) = 171.95 ppm in **12** versus 163.16 ppm in **6**; δ_C_ (6 = CH_2_) = 95.26 in **12** versus 100.44 ppm in **6**, spectra measured in C_6_D_6_).^33^ Compound **11** (Figures S51–S55, Table S10) was confirmed to be bicycloprechlororaphen (**11**), the first neutral intermediate produced by cyclization of α-PCPP (**5a**) by PchlO6_6041 in the biosynthesis of **6** as recently shown by the Dickschat laboratory.^33^

## Discussion

Canonical terpene biosynthesis involves assembly of five-carbon isoprene units into C_5*n*_ scaffolds.^62^ In contrast, noncanonical terpenoids resulting from cyclization of early biosynthetic precursors that do not adhere to this biosynthetic logic remain exceptionally rare natural products.^63^ *Pseudomonas* species exhibit underexplored potential for generating such compounds, with current knowledge limited to a few reports of FPP-derived C_16_ terpenes and a single C_17_ homologue.^21,28,31^ In this study, we utilized an *E. coli* heterologous expression platform to successfully characterize a FPP-derived C_17_ terpene, grimophan (**7**), originating from *Pseudomonas grimontii* DSM 17515. Grimophan (**7**) represents the second FPP-derived C_17_ terpene structural framework with a unique deltacyclane skeleton.

Our work established a uniform paradigm for the biosynthesis of FPP-derived C_17_ terpenes, such as **6** and **7**. Initially, the common intermediate α-PCPP (**5a**) is produced by two different groups of MTs. Similar to SodC in the biosynthesis of C_16_ terpenes,^27,40^ the MT group I comprising PgrC or PchlO6_6045 is bifunctional, catalyzing methylation and cyclization reactions to yield γ-PSPP (**4a**). Subsequently, group II of highly homologous MTs, including PgrD and PchlO6_6042, catalyzes additional methylation of **4a** to produce the common intermediate **5a**. The final C_17_ terpenoid structures are diversified by TSs, here, leading to **6** and **7** catalyzed by PchlO6_6041 and PgrE, respectively. The occurrence of a common intermediate **5a** generated by the MTs PgrC and PgrD or their homologues now offers a streamlined strategy for targeted mining of C_17_ terpenes by simple insertion of the TS-encoding genes to be studied into a *pgrCD*-containing expression plasmid.

Despite the presence of three MTs within the *pgr* BGC, only two methylation events occur in grimophan (**7**) biosynthesis. The seemingly superfluous third MT PgrF belongs to highly conserved group III of MTs widely distributed among *Pseudomonas* species. Within this work, we uncovered that PgrF and its homologues significantly enhance the production titers of noncanonical terpenes. While PgrF exhibits strong protein–protein interactions with both functional MTs, allowing for copurification of PgrF with PgrC or PgrD, we have shown that this enzyme does not merely increase catalytic efficiency of the methylation steps in the biosynthesis of **7**. The significant increase of production titers (>4-fold) is only achieved in the presence of all essential biosynthetic enzymes, including the TS. This suggests PgrF to act as a scaffolding cofactor that facilitates formation of a highly efficient biosynthetic enzyme complex, likely increasing the entire substrate processing cascade and hence overall production titer. While the exact molecular mechanism triggered by PgrF-type enzymes remains to be elucidated, they can now serve as tools for the efficient biotechnological production of unusual terpenoids.

Notably, PgrE and PchlO6_6041, sharing over 88% identity at the amino acid sequence level, catalyze the conversion of **5a** to produce two different products, grimophan (**7**) and chlororaphen A (**6**), respectively. Based on AlphaFold3 protein structure models, we conducted strategic changes to PgrE by single-point mutations to shed first light on sequence-product relationships. Concentrating on changes to the conserved D^92^DMPLG^97^ signature, our results show that dramatic changes in product profiles can be achieved by single-point mutations that directly affect the spatial arrangement and electronic properties of the PgrE active site. Such targeted mutations provide tremendous potential for enzyme engineering, targeting access to novel C_17_ terpene scaffolds with diversified architectures. This is exemplified by the formation of bicyclic compound **11**, a known shunt product of chlororaphen biosynthesis,^33^ as well as the new chlororaphen analogue **12**, both observed in this study. Notably, the stereochemical configuration of **11** is identical to that reported for **11** in chlororaphen biosynthesis,^33^ whereas **7**—aside from the additional cyclopropane ring—exhibits an inverted configuration at C8, and **12** corresponds to 7,8-epi-chlororaphen A. These findings raise fundamental questions regarding the stereochemical course of product formation.

Guided by the excellent comprehensive mechanistic analysis of chlororaphen biosynthesis by the Dickschat laboratory,^33^ we propose a unifying biosynthetic mechanism accounting for the observed stereochemical outcomes (Scheme S64). Starting from precursor **5a**, loss of the pyrophosphate unit generates carbocation **I1**, which undergoes *C*,*C*-bond formation between C9 and C5 to give **I2**. From this intermediate, the pathways leading to **11** versus **7**/**12** diverge and this branching point is highly sensitive to mutations at position D93. Whereas the most nonconservative mutation D93L results in near-complete loss of PgrE activity, the conservative mutation D93E retains catalytic competence, producing small amounts of **7**, comparable levels of **11**, and approximately 3-fold higher levels of **12** (Figure 6D). In contrast, mutations D93N, D93S, and D93C display reduced overall activity but exhibit near-exclusive selectivity for the formation of **11**. We attribute this pronounced selectivity shift to alternative hydride-shift reactions from **I2**. In wild-type PgrE, a 1,3-hydride shift leads to **I4** and ultimately to **7**/**12** (see below). In contrast, D93 variants enable a 1,2-hydride shift to **I3**, closely paralleling the reactivity of PchlO6_6041 in chlororaphen biosynthesis.^33^ This process occurs with retention of the hydrogen spatial orientation, accounting for the observed configuration at C8. Subsequent deprotonation at C1 yields a C1,C7 double bond, furnishing shunt product **11**.

In wild-type PgrE and all other tested mutants, the dominant reaction pathway from **I2** involves a 1,3-hydride shift from C1 to C8, again with retention of the hydrogen orientation, resulting in inversion of the stereocenter at C8 when compared to **11** and chlororaphen A (**6**). Proton abstraction at C7 then generates a C1,C7 bond, affording 8-epi-**11**. Protonation of the terminal double bond yields **I5**, featuring charge delocalization over C6 and the C1,C7 double bond, analogous to a central intermediate in chlororaphen biosynthesis.^33^ Completion of grimpophan (**7**) biosynthesis proceeds by cyclopropanation initiated by deprotonation at C6. This transformation is tightly controlled in wild-type PgrE and remains predominant in the G97D variant, both of which are predicted to adopt a closed protein conformation.

Consistent with this model, mutations at L96, predicted to induce a conformational shift from a closed (wild-type) to a more open state (resembling PchlO6_6041), markedly alter the product distribution. While the relatively conservative mutations L96S and L96C maintain production of **7** at near wild-type levels but exhibit substantial additional formation of **12**, the mutations L96D and L96N lead to a pronounced reduction in **7**, with **12** emerging as the dominant product and **11** formed in amounts comparable to **7**. Mechanistically, this outcome can be rationalized by suppression of cyclopropanation in favor of the *C*,*C*-bond formation between C1 and C6, generating **I6**. A subsequent 1,3-hydride shift from C6 to C7 yields **I7**, accounting for the observed configuration at C7 in **12**, which is ultimately formed from **I7** by deprotonation/double-bond formation.

## Conclusions

In conclusion, our work provides detailed functional insights into the biosynthesis of the FPP-derived C_17_ terpene grimophan (**7**) from *Pseudomonas* sp., using heterologous expression in *E. coli* and in-depth *in vitro* characterization of all pathway enzymes PgrCDEF. Two methyltransferases, PgrC and PgrD, convert FPP (**1a**) into the common intermediate **5a**, which is subsequently cyclized by the terpene synthase PgrE to yield **7**. We also identified a methyltransferase-like enzyme, PgrF, that significantly enhances production titers of **7** in the presence of the full set of biosynthetic enzymes, PgrCDEF, suggesting a scaffolding role that improves the overall pathway efficiency. Furthermore, strategic single-point mutations in the D_92_DMPLG_97_ motif of PgrE revealed how subtle active-site alterations redirect product selectivity, enabling the formation of structurally altered terpenoids. Together, these findings expand the structural diversity of noncanonical terpenoids, advance our understanding of their biosynthetic assembly, and provide a foundation for their targeted discovery and engineering. While the effects of PgrE mutagenesis on product profiles are carefully experimentally validated, the structural impact on PgrE is solely inferred from AlphaFold predictions. Future experimental protein structural studies are needed to corroborate these predictions. In addition, detailed biochemical investigations will be required to confirm and mechanistically map the scaffolding role of PgrF within the pathway. This work is currently underway in our laboratory.

Since the discovery of sodorifen (**3**) in 2010,^27^ the body of work on related to noncanonical C_16_, and more recently C_17_ terpenes, has significantly expanded. Biosynthetic work on **3** ^20,64–67^ has set the stage for the development of heterologous production platforms in *E. coli*,^40^ the targeted discovery of biosynthetically related novel C_16_ ^19,21,31,68^ and C_17_ terpenoid^28^ scaffolds also beyond bacterial producers,^69^ and the elucidation of highly sophisticated and unusual assembly mechanisms.^32,33,70^ The emerging picture of the widespread occurrence and chemical diversity of such terpenoids raises questions on their potential ecological functions. Work by Piechulla and co-workers has shown that carbon catabolite repression regulates production of **3**,^71^ that this compound from rhizosphere-derived bacteria has no impact on growth of *Arabidopsis thaliana*,^72^ and that cocultivation of *Serratia plymuthica* with *Bacillus subtilis* leads to its enhanced production.^73^ In addition, volatile natural products from fungi (*Fusarium culmorum*) were shown to induce production of **3**.^74^ Combined, these reports strongly suggest that sodorifen (**3**) might play important roles in selective intra- and/or interspecies communication, given the volatile nature of the compound putatively over longer distances. Further studies are needed to elucidate the functional diversity of known and to-be-discovered C_16_ and C_17_ terpenoids and their roles in communication.

## Supplementary Material



## Data Availability

All data that support this study are publicly available as of the date of publication. This paper does not report original code. The NCBI RefSeq Genome Database (https://www.ncbi.nlm.n-ih.gov/refseq/) and Pseudomonas Genome DB (https://www.pseudomonas.com/) were used for BGC analysis, and the identified IDs are indicated in the Supporting Information file. Any additional information required to reanalyze the data reported in this paper is available from the lead contact upon request.

## Notes

Full experimental details, sequence alignments of MTs and TSs, GC-MS analyses, spectrometric and spectroscopic characterization data, bioinformatic data, and protein structure predictions generated using AlphaFold (PDF)

## References

[1] Li Y. (2023). Analytical
Methods for the Analysis of Volatile Natural Products. Nat. Prod. Rep..

[2] Tholl, D. Biosynthesis and Biological Functions of Terpenoids in Plants. In Biotechnology of Isoprenoids; Schrader, J. , Bohlmann, J. , Eds.; Springer International Publishing: Cham, 2015; pp 63–106. 10.1007/10_2014_295. 25583224

[3] Wei G., Eberl F., Chen X., Zhang C., Unsicker S. B., Köllner T. G., Gershenzon J., Chen F. (2020). Evolution of Isoprenyl
Diphosphate Synthase-like Terpene Synthases in Fungi. Sci. Rep..

[4] González-Hernández R. A., Valdez-Cruz N. A., Macías-Rubalcava M. L., Trujillo-Roldán M. A. (2023). Overview
of Fungal Terpene Synthases and Their Regulation. World J. Microbiol. Biotechnol..

[5] Silvestre, A. J. D. ; Gandini, A. Chapter 2 - Terpenes: Major Sources, Properties and Applications. In Monomers, Polymers and Composites from Renewable Resources; Belgacem, M. N. , Gandini, A. , Eds.; Elsevier: Amsterdam, 2008; pp 17–38. 10.1016/B978-0-08-045316-3.00002-8.

[6] Abu-Izneid T., Rauf A., Shariati M. A., Khalil A. A., Imran M., Rebezov M., Uddin Md. S., Mahomoodally M. F., Rengasamy K. R. R. (2020). Sesquiterpenes and Their Derivatives-Natural
Anticancer
Compounds: An Update. Pharmacol. Res..

[7] Daviet L., Schalk M. (2010). Biotechnology in Plant
Essential Oil Production: Progress
and Perspective in Metabolic Engineering of the Terpene Pathway. Flavour Fragr. J..

[8] Frank A., Groll M. (2017). The Methylerythritol
Phosphate Pathway to Isoprenoids. Chem. Rev..

[9] Zhao Y., Yang J., Qin B., Li Y., Sun Y., Su S., Xian M. (2011). Biosynthesis
of Isoprene in Escherichia Coli via Methylerythritol
Phosphate (MEP) Pathway. Appl. Microbiol. Biotechnol..

[10] Geron C., Rasmussen R., R. Arnts R., Guenther A. (2000). A Review and Synthesis
of Monoterpene Speciation from Forests in the United States. Atmos. Environ..

[11] Ruan J.-X., Li J.-X., Fang X., Wang L.-J., Hu W.-L., Chen X.-Y., Yang C.-Q. (2016). Isolation and Characterization
of
Three New Monoterpene Synthases from *Artemisia Annua*. Front. Plant Sci..

[12] Merfort I. (2002). Review of
the Analytical Techniques for Sesquiterpenes and Sesquiterpene Lactones. J. Chromatogr. A.

[13] Cordell G. A. (1976). Biosynthesis
of Sesquiterpenes. Chem. Rev..

[14] Fan Y.-Y., Xu J.-B., Liu H.-C., Gan L.-S., Ding J., Yue J.-M. (2017). Cephanolides A–J,
Cephalotane-Type Diterpenoids
from *Cephalotaxus Sinensis*. J. Nat. Prod..

[15] De
Sousa I. P., Sousa Teixeira M. V., Jacometti Cardoso Furtado N. A. (2018). An Overview
of Biotransformation and Toxicity of Diterpenes. Molecules.

[16] Noushahi H. A., Khan A. H., Noushahi U. F., Hussain M., Javed T., Zafar M., Batool M., Ahmed U., Liu K., Harrison M. T., Saud S., Fahad S., Shu S. (2022). Biosynthetic
Pathways of Triterpenoids and Strategies to Improve Their Biosynthetic
Efficiency. Plant Growth Regul..

[17] Basyuni M., Oku H., Tsujimoto E., Kinjo K., Baba S., Takara K. (2007). Triterpene
Synthases from the Okinawan Mangrove Tribe, Rhizophoraceae. FEBS J..

[18] Sommer S., Lang L. M., Drummond L., Buchhaupt M., Fraatz M. A., Zorn H. (2022). Odor Characteristics
of Novel Non-Canonical
Terpenes. Molecules.

[19] Ignea C., Raadam M. H., Koutsaviti A., Zhao Y., Duan Y.-T., Harizani M., Miettinen K., Georgantea P., Rosenfeldt M., Viejo-Ledesma S. E., Petersen M. A., Bredie W. L. P., Staerk D., Roussis V., Ioannou E., Kampranis S. C. (2022). Expanding
the Terpene Biosynthetic Code with Non-Canonical 16 Carbon Atom Building
Blocks. Nat. Commun..

[20] Piechulla B., Zhang C., Eisenschmidt-Bönn D., Chen F., Magnus N. (2021). Non-Canonical Substrates for Terpene
Synthases in Bacteria
Are Synthesized by a New Family of Methyltransferases. FEMS Microbiol. Rev..

[21] Mo X. H., Pu Q. Y., Lübken T., Yu G. H., Malay M., D’Agostino P. M., Gulder T. A. M. (2024). Discovery and Biosynthesis of Non-Canonical
C16-Terpenoids from Pseudomonas. Cell Chem.
Biol..

[22] Jiang J., He X., Cane D. E. (2006). Geosmin Biosynthesis. *Streptomyces Coelicolor* Germacradienol/Germacrene D Synthase Converts Farnesyl Diphosphate
to Geosmin. J. Am. Chem. Soc..

[23] Herde M., Gärtner K., Köllner T. G., Fode B., Boland W., Gershenzon J., Gatz C., Tholl D. (2008). Identification and
Regulation of TPS04/GES, an Arabidopsis Geranyllinalool Synthase Catalyzing
the First Step in the Formation of the Insect-Induced Volatile C16-Homoterpene
TMTT. Plant Cell.

[24] Degenhardt J., Gershenzon J. (2000). Demonstration
and Characterization of (*E*)-Nerolidol Synthase from
Maize: A Herbivore-Inducible Terpene Synthase
Participating in (3*E*)-4,8-Dimethyl-1,3,7-Nonatriene
Biosynthesis. Planta.

[25] Judy K. J., Schooley D. A., Dunham L. L., Hall M. S., Bergot B. J., Siddall J. B. (1973). Isolation, Structure,
and Absolute Configuration of
a New Natural Insect Juvenile Hormone from *Manduca Sexta*. Proc. Natl. Acad. Sci. U. S. A..

[26] Qu Z., Bendena W. G., Tobe S. S., Hui J. H. L. (2018). Juvenile Hormone
and Sesquiterpenoids in Arthropods: Biosynthesis, Signaling, and Role
of MicroRNA. J. Steroid Biochem. Mol. Biol..

[27] von
Reuß S. H., Kai M., Piechulla B., Francke W. (2010). Octamethylbicyclo­[3.2.1]­Octadienes from the Rhizobacterium *Serratia Odorifera*. Angew. Chem.,
Int. Ed..

[28] Magnus N., Von Reuss S. H., Braack F., Zhang C., Baer K., Koch A., Hampe P. L., Sutour S., Chen F., Piechulla B. (2023). Non-canonical Biosynthesis of the Brexane-Type Bishomosesquiterpene
Chlororaphen through Two Consecutive Methylation Steps in *Pseudomonas Chlororaphis* O6 and *Variovorax Boronicumulans* PHE5–4. Angew. Chem., Int. Ed..

[29] Schulz S., Dickschat J. (2007). Bacterial Volatiles: The Smell of Small Organisms. Nat. Prod. Rep..

[30] Xu H., Dickschat J. S. (2022). Mechanistic
Investigations on Microbial Type I Terpene
Synthases through Site-Directed Mutagenesis. Synthesis.

[31] Duan Y.-T., Koutsaviti A., Harizani M., Ignea C., Roussis V., Zhao Y., Ioannou E., Kampranis S. C. (2023). Widespread
Biosynthesis of 16-Carbon Terpenoids in Bacteria. Nat. Chem. Biol..

[32] Xu H., Lauterbach L., Goldfuss B., Schnakenburg G., Dickschat J. S. (2023). Fragmentation
and [4 + 3] Cycloaddition in Sodorifen
Biosynthesis. Nat. Chem..

[33] Xu H., Li H., Goldfuss B., Schnakenburg G., Dickschat J. S. (2024). Biosynthesis
of the Non-Canonical C17 Sesquiterpenoids Chlororaphen A and B from *Pseudomonas Chlororaphis*. Angew. Chem.,
Int. Ed..

[34] Duan Y. T., Zhang C., Koutsaviti A., Tammam M. A., Harizani M., Roussis V., Zhao Y., Ioannou E., Kampranis S. C. (2025). Biosynthesis of 17-Carbon Terpenoids
in Bacteria. J. Am. Chem. Soc..

[35] Freeman P. K., Stevenson B. K. (1973). Carbonium Ion Rearrangements in the Deltacyclane Ring
System. III. Solvolytic Reactions of C-5 Substituted Exo- and Endo-8-Deltacyclyl
Brosylates. J. Am. Chem. Soc..

[36] Gilchrist C. L. M., Chooi Y.-H. (2021). Clinker & Clustermap.Js: Automatic Generation of
Gene Cluster Comparison Figures. Bioinformatics.

[37] Letunic I., Bork P. (2024). Interactive Tree of Life (iTOL) v6: Recent Updates to the Phylogenetic
Tree Display and Annotation Tool. Nucleic Acids
Res..

[38] Huerta-Cepas J., Serra F., Bork P. (2016). ETE 3: Reconstruction, Analysis,
and Visualization of Phylogenomic Data. Mol.
Biol. Evol..

[39] Crooks G. E., Hon G., Chandonia J.-M., Brenner S. E. (2004). WebLogo: A Sequence Logo Generator. Genome Res..

[40] Duell E. R., D’Agostino P. M., Shapiro N., Woyke T., Fuchs T. M., Gulder T. A. M. (2019). Direct
Pathway Cloning of the Sodorifen Biosynthetic
Gene Cluster and Recombinant Generation of Its Product in E. Coli. Microb. Cell Factories.

[41] Yuan W., Lv S., Chen L., Zhao Y., Deng Z., Hong K. (2019). Production
of Sesterterpene Ophiobolin by a Bifunctional Terpene Synthase in
Escherichia Coli. Appl. Microbiol. Biotechnol..

[42] Reiling K. K., Yoshikuni Y., Martin V. J. J., Newman J., Bohlmann J., Keasling J. D. (2004). Mono and Diterpene Production in Escherichia Coli. Biotechnol. Bioeng..

[43] Greunke C., Duell E. R., D’Agostino P.
M., Glöckle A., Lamm K., Gulder T. A. M. (2018). Direct Pathway Cloning (DiPaC) to
Unlock Natural Product Biosynthetic Potential. Metab. Eng..

[44] Qian Z., Bruhn T., D’Agostino P.
M., Herrmann A., Haslbeck M., Antal N., Fiedler H.-P., Brack-Werner R., Gulder T. A. M. (2020). Discovery of the Streptoketides by Direct Cloning and
Rapid Heterologous Expression of a Cryptic PKS II Gene Cluster from *Streptomyces* Sp. Tü 6314. J.
Org. Chem..

[45] D’Agostino P. M., Gulder T. A. M. (2018). Direct Pathway Cloning Combined with Sequence- and
Ligation-Independent Cloning for Fast Biosynthetic Gene Cluster Refactoring
and Heterologous Expression. ACS Synth. Biol..

[46] Ouyang X., D'Agostino P. M., Wahlsten M., Delbaje E., Jokela J., Permi P., Gaiani G., Poso A., Bartos P., Gulder T. A. M., Koistinen H., Fewer D. P. (2023). Direct Pathway Cloning
and Expression of the Radiosumin Biosynthetic Gene Cluster. Org. Biomol. Chem..

[47] Li, M. Z. ; Elledge, S. J. SLIC: A Method for Sequence- and Ligation-Independent Cloning. In Gene Synthesis: Methods and Protocols; Peccoud, J. , Ed.; Humana Press: Totowa, NJ, 2012; pp 51–59. 10.1007/978-1-61779-564-0_5. 22328425

[48] Yu J. S., Kleckley T. S., Wiemer D. F. (2005). Synthesis of Farnesol
Isomers via
a Modified Wittig Procedure. Org. Lett..

[49] Wang G.-P., Yu X.-D., Fan J., Wang C.-S., Xia L.-Q. (2015). Expressing
an (E)-β-Farnesene Synthase in the Chloroplast of Tobacco Affects
the Preference of Green Peach Aphid and Its Parasitoid. J. Integr. Plant Biol..

[50] Ueda D., Yamaga H., Murakami M., Totsuka Y., Shinada T., Sato T. (2015). Biosynthesis of Sesterterpenes,
Head-to-Tail Triterpenes, and Sesquarterpenes
in *Bacillus Clausii*: Identification of Multifunctional
Enzymes and Analysis of Isoprenoid Metabolites. ChemBioChem..

[51] Hou A., Dickschat J. S. (2020). The Biosynthetic Gene Cluster for Sestermobaraenes—Discovery
of a Geranylfarnesyl Diphosphate Synthase and a Multiproduct Sesterterpene
Synthase from *Streptomyces Mobaraensis*. Angew. Chem., Int. Ed..

[52] Sato T., Yamaga H., Kashima S., Murata Y., Shinada T., Nakano C., Hoshino T. (2013). Identification
of Novel Sesterterpene/Triterpene
Synthase from *Bacillus Clausii*. Chembiochem.

[53] Kong L., Wang Q., Yang W., Shen J., Li Y., Zheng X., Wang L., Chu Y., Deng Z., Chooi Y.-H., You D. (2020). Three Recently Diverging Duplicated
Methyltransferases Exhibit Substrate-Dependent Regioselectivity Essential
for Xantholipin Biosynthesis. ACS Chem. Biol..

[54] Park M. R., Chen X., Lang D. E., Ng K. K. S., Facchini P. J. (2018). Heterodimeric
O-Methyltransferases Involved in the Biosynthesis of Noscapine in *Opium Poppy*. Plant J..

[55] Schubert H. L., Blumenthal R. M., Cheng X. (2003). Many Paths to Methyltransfer: A Chronicle
of Convergence. Trends Biochem. Sci..

[56] Hobble H. V., Schaner Tooley C. E. (2024). Intrafamily Heterooligomerization as an Emerging Mechanism
of Methyltransferase Regulation. Epigenetics
Chromatin.

[57] Park T., Won J., Baek M., Seok C. (2021). GalaxyHeteromer: Protein Heterodimer
Structure Prediction by Template-Based and Ab Initio Docking. Nucleic Acids Res..

[58] Heo L., Lee H., Seok C. (2016). GalaxyRefineComplex:
Refinement of Protein-Protein
Complex Model Structures Driven by Interface Repacking. Sci. Rep..

[59] Schrödinger, LLC . PyMOL Molecular Graphics System, Version 1.8, 2015.

[60] Baer P., Rabe P., Fischer K., Citron C. A., Klapschinski T. A., Groll M., Dickschat J. S. (2014). Induced-Fit Mechanism in Class I
Terpene Cyclases. Angew. Chem., Int. Ed..

[61] Abramson J., Adler J., Dunger J., Evans R., Green T., Pritzel A., Ronneberger O., Willmore L., Ballard A. J., Bambrick J., Bodenstein S. W., Evans D. A., Hung C. C., O’Neill M., Reiman D., Tunyasuvunakool K., Wu Z., Žemgulytė A., Arvaniti E., Beattie C., Bertolli O., Bridgland A., Cherepanov A., Congreve M., Cowen-Rivers A. I., Cowie A., Figurnov M., Fuchs F. B., Gladman H., Jain R., Khan Y. A., Low C. M. R., Perlin K., Potapenko A., Savy P., Singh S., Stecula A., Thillaisundaram A., Tong C., Yakneen S., Zhong E. D., Zielinski M., Žídek A., Bapst V., Kohli P., Jaderberg M., Hassabis D., Jumper J. M. (2024). Accurate Structure
Prediction of Biomolecular Interactions with AlphaFold 3. Nature.

[62] Vranová E., Coman D., Gruissem W. (2013). Network Analysis
of the MVA and MEP
Pathways for Isoprenoid Synthesis. Annu. Rev.
Plant Biol..

[63] Kakumu Y., Chaudhri A. A., Helfrich E. J. N. (2025). The
Role and Mechanisms of Canonical
and Non-Canonical Tailoring Enzymes in Bacterial Terpenoid Biosynthesis. Nat. Prod. Rep..

[64] Domik D., Thürmer A., Weise T., Brandt W., Daniel R., Piechulla B. (2016). A Terpene Synthase Is Involved in the Synthesis of
the Volatile Organic Compound Sodorifen of *Serratia Plymuthica* 4Rx13. Front. Microbiol..

[65] Domik D., Magnus N., Piechulla B. (2016). Analysis of
a New Cluster of Genes
Involved in the Synthesis of the Unique Volatile Organic Compound
Sodorifen of *Serratia Plymuthica* 4Rx13. FEMS Microbiol. Lett..

[66] von
Reuss S., Domik D., Lemfack M. C., Magnus N., Kai M., Weise T., Piechulla B. (2018). Sodorifen Biosynthesis in the Rhizobacterium
Serratia Plymuthica Involves Methylation and Cyclization of MEP-Derived
Farnesyl Pyrophosphate by a SAM-Dependent C-Methyltransferase. J. Am. Chem. Soc..

[67] Lemfack M. C., Brandt W., Krüger K., Gurowietz A., Djifack J., Jung J.-P., Hopf M., Noack H., Junker B., von Reuß S., Piechulla B. (2021). Reaction Mechanism
of the Farnesyl Pyrophosphate C-Methyltransferase towards the Biosynthesis
of Pre-Sodorifen Pyrophosphate by *Serratia Plymuthica* 4Rx13. Sci. Rep..

[68] Reuter T., Dieminger L., Steidle S., Zoller K., Holocher M., Zhou L., Hanauska D. M., Racz K., Barra L. (2025). Non-Canonical
C16 Homoterpene Biosynthesis Widespread in Actinobacteria. Angew. Chem., Int. Ed..

[69] Zhou L., Reuter T., Schumann K., Mayer M., Hanauska D. M., Barra L. (2025). Homoterpene Biosynthesis
in Fungi. Angew. Chem.,
Int. Ed..

[70] Xu H., Goldfuss B., Dickschat J. S. (2024). Common Biosynthesis of Non-Canonical
C16 Terpenes through a Fragmentation-Recombination Mechanism. Angew. Chem., Int. Ed..

[71] Magnus N., Weise T., Piechulla B. (2017). Carbon Catabolite
Repression Regulates
the Production of the Unique Volatile Sodorifen of *Serratia
Plymuthica* 4Rx13. Front. Microbiol..

[72] Kai M., Crespo E., Cristescu S. M., Harren F. J. M., Francke W., Piechulla B. (2010). Serratia Odorifera:
Analysis of Volatile Emission and
Biological Impact of Volatile Compounds on Arabidopsis Thaliana. Appl. Microbiol. Biotechnol..

[73] Kai M., Piechulla B. (2018). Interspecies
Interaction of *Serratia Plymuthica* 4Rx13 and *Bacillus Subtilis* B2g Alters the Emission
of Sodorifen. FEMS Microbiol. Lett..

[74] Schmidt R., Jager V. d., Zühlke D., Wolff C., Bernhardt J., Cankar K., Beekwilder J., Ijcken W. v., Sleutels F., Boer W. d., Riedel K., Garbeva P. (2017). Fungal Volatile Compounds
Induce Production of the Secondary Metabolite Sodorifen in *Serratia Plymuthica* PRI-2C. Sci. Rep..

