# Supplementary material for: Discovery and Biosynthesis of Farnesyl Pyrophosphate-Derived Noncanonical C17 Terpenes from Pseudomonas Species
Source: J Am Chem Soc. 2026 Mar 16;148(11):11939–52. doi: 10.1021/jacs.5c21930 (PMC13022886; doi:10.1021/jacs.5c21930)
Supplement: Supplementary file 1 [file ja5c21930_si_001.pdf]

Discovery and Biosynthesis of Farnesyl Pyrophosphate-Derived Non-Canonical C_{17} Terpenes from *Pseudomonas* Species

Qing-Yin Pu,[1] Xu-Hua Mo,[2,*] Tilo Lübken,[3] Manuel Einsiedler,[1] Tobias A. M. Gulder[1,4,*]

[1] Chair of Technical Biochemistry, Technische Universität Dresden, Bergstraße 66, 01069 Dresden, Germany.

[2] Shandong Provincial Key Laboratory of Microbial Resource Exploration and Innovative Utilization, School of Life Sciences, Qingdao Agricultural University, Qingdao 266109, China.

[3] Chair of Organic Chemistry I, Technische Universität Dresden, Bergstraße 66, 01069 Dresden, Germany

[4] Department of Natural Product Biotechnology, Helmholtz Institute for Pharmaceutical Research Saarland (HIPS), Helmholtz Centre for Infection Research (HZI) and Department of Pharmacy at Saarland University, PharmaScienceHub (PSH), Campus E8.1, 66123 Saarbrücken, Germany.

[*] Correspondence: xhmo@qau.edu.cn (X.H.M.); tobias.gulder@helmholtz-hips.de (T.A.M.G)

Supplementary Information

Content

Materials.. 3

Experimental Methods 3

Supplementary Tables 10

Supplementary Figures... 26

Structure assignments including NMR and HRMS spectra 55

Proposed biosynthetic mechanism 83

References 84

Materials

Luria-Bertani broth (LB), Terrific broth (TB), *S*-adenosyl-L-methionine (SAM) (CAS# 29908-03-0) and (*E*,*E*)-farnesyl chloride were obtained from Sigma Aldrich. Primers were purchased from Sigma and DNA sequencing was performed by Genewiz (https://clims4.genewiz.com/). Q5 High-fidelity DNA polymerase (catalog no. M0491S, NEB) was used in PCR amplifications. Monarch Plasmid Miniprep Kit (catalog no. T1010S, NEB) was used for plasmid DNA purification. DNA Gel Extraction Kit (catalog no. T1020S, NEB) and PCR & DNA Cleanup Kit (5 μg) (catalog no. T1030S, NEB) were used for gel extraction and DNA purification. T4 DNA polymerase (catalog no. M0203S, NEB) was used for plasmid construction by using one-step Sequence- and Ligation-Independent Cloning (SLIC). PureCube 100 Ni-NTA Agarose (catalog no. 74103, Cube Biotech), Pierce™ Glutathion-Agarose (catalog no.16100, Thermo Scientific) and PD-10 Desalting Columns with Sephadex G-25 resin (catalog no. 17085101, Cytiva Life Sciences) were used for protein purification and desalting. Pierce BCA Protein Assay Kit (catalog no. 23225, Thermo Scientific) was used to accurately determine protein concentration. All chemicals and antibiotic used in this study were obtained from Sigma Aldrich and New England Biolabs (NEB) unless otherwise specified. Solvents for HPLC and MS analysis, such as acetonitrile and methanol, were purchased from Fisher Scientific and VWR in a purity of over 99% (HPLC-grade). Water was purified and deionized using a TKA GenPure water treatment system.

Experimental Methods

Bioinformatic analysis

The genome sequence data of *Pseudomonas grimontii* strain DSM 17515 and *Pseudomonas chlororaphis* subsp. *chlororaphis* DSM 50083 were downloaded from the NCBI database (https://www.ncbi.nlm.nih.gov/) using NCBI RefSeq assembly ID GCA_007858185.1 and GCF_003945765.1, respectively. The remaining genome information for this study is shown in Supplementary Tables S1-S3. All sequences were organized and stored within the SnapGene software package (GSL Biotech, version 6.0.2) to develop the cloning strategies, allow construction of plasmid maps and primers design. DiPaC primer design was done using the NEBuilder assembly web tool (NEB, http://nebuilder.neb.com, version 2.7.0) followed by manual adjustment of primer sequences to reduce secondary structure. BGC analyses were performed using AntiSMASH (version 5). *Pseudomonas* sp. genomes were retrieved from the Pseudomonas Genome Database (https://www.pseudomonas.com). Multiple sequence alignment and phylogenetic reconstructions were performed using the function "build" of ETE3 3.1.3[1] as implemented on the GenomeNet (https://www.genome.jp/tools/ete/). Sequence logos were created by uploading amino acid sequences to the WebLogo web (https://weblogo. berkeley.edu/).

Gene cloning and DNA assembly

Pseudomonas grimontii strain DSM 17515 was obtained from the German Collection of Microorganisms and Cell Cultures (DSMZ) and cultivated in medium 535 (3% Trypticase Soy Broth, pH 7.3) at 30 °C under 36 hours. Genomic DNA extraction from *P. grimontii* was performed with harvested cells from 5 mL culture, following an optimized method with bacterial genomic DNA preparation column kit (PP-214L, Jena Bioscience). The gDNA was dissolved in ddH_2O and quantified using a P330 NanoPhotometer (Implen, Germany), then stored at -20 °C for further use. Genomic DNA of *P. chlororaphis* subsp. *chlororaphis* DSM 50083 was obtained from a previous

study[2]. All linear fragments of BGCs, individual genes, and linear vectors were amplified by PCR with Q5 High Fidelity DNA Polymerase, in accordance with the protocol from NEB for DNA assembly. All amplified PCR products after purification using DNA Gel Extraction Kit and Cleanup Kit were directly treated with DpnI (NEB) at 37 °C to eliminate template DNA as transformation background. Subsequently, one-step sequence and ligation independent cloning (SLIC) assembly was conducted as recommended in the NEB protocol and published reference[2]. The constructs were transformed into chemically competent *E. coli* DH5α for further study. Colony PCR was initially performed to screen positive clones of all constructs with subsequent restriction digestion, followed by Sanger sequencing to finally confirm successful and mutation-free cloning. In this study, the linear pET28-ptetO-GFPv2 vector was used as backbone to construct plasmids for BGC expression in *E. coli* BAP1, while the linear pHis8-TEV vector was employed to assemble plasmids for protein overexpression in *E. coli* BL21(DE3). Additionally, the linear pGS-21a::ΔHis8 vector was utilized to generate plasmids for GST-PgrF protein overexpression. The primers used in this study are listed in Supplementary Table S6, while bacterial strains used are listed in Supplementary Table S7.

Production and isolation of volatile terpenes

A BIOSTAT A plus fermenter (type 8843812, Sartorius Stedim Biotech) was used to cultivate *P. grimontii* DSM 17515 and all produced recombinant *E. coli* BAP1 strains with 0.3 L/min air flow rate. Bacterial fermentation of *Pseudomonas grimontii* DSM 17515 was performed in 3 L DSM Medium 1 (5‰ w/v Peptone, 3‰ w/v Meat extract, pH 7.0) with 1‰ (v/v) antifoam SE-15 for 5 days at 30 °C. The constructed pET28b-ptetO-based plasmids were chemically transformed into *E.coli* BAP1. Resulting *E. coli* BAP1 derivative strains were used to generate 50 mL pre-cultures grown in LB medium supplemented with 50 µg/mL kanamycin and incubated overnight at 37 °C with shaking at 180 rpm. Expression cultures were inoculated with 1% (v/v) pre-culture in 3 L TB medium supplemented with 50 µg/mL kanamycin and 1‰ (v/v) antifoam SE-15. Expression cultures were incubated at 37 °C with 180 rpm until an optical density at 600 nm (OD_{600}) of 0.8-1.0 was reached. Cultures were cooled to 20 °C for 30 min, induced with 0.75 µg/mL tetracycline and incubated for 3-5 days at 20 °C with constant stirring and pH maintained between 6.8-7.6. The fermenter was covered to exclude light and hence reduce light-induced decomposition of tetracycline.

The production of terpenoids was performed using the *E. coli* BAP1 system and the above-described method in a total of 9 L of TB medium. VOCs were collected on pre-activated charcoal at the gas outlet of the fermenter with VitraPOR glass filters (porosity 00, ROBU). Filters were exchanged every 24 hours for 5 days of fermentation, using ca. 1.0 g of charcoal per filter. The charcoal samples loaded with VOCs were extracted twice with 4 mL of *n*-pentane in total. The combined *n*-pentane extracts were concentrated to approximately 500 µL under a gently stream of argon with continuous cooling on ice, as previously described[2]. The solution was then purified by micro-column chromatography on a 1 mm diameter glass column filled with silica (60 Å, 70-230 mesh, 63-200 µm; Sigma) and pentane as eluent. Fraction purity was assessed by GC-EI-MS analysis and pure fractions combined accordingly. After removing the pentane under a gentle stream of argon on ice, compounds were dissolved in $CDCl_3$ or C_6D_6 for NMR analysis. Following this procedure, 8 mg of **7** (optical rotation: $[\alpha]^{25}D$ = -43.7 (*c* 3.66, $CDCl_3$)) were isolated from expression experiments using pET28-ptetO-gfpv2::pgr*CDEF*; 1.6 mg of **8** were obtained from cultures containing pET28-ptetO-gfpv2::*pgrCE*; 6 mg of **10** were isolated from cultures containing pET28-

ptetO-gfpv2::*pgrE*; a combined yield of 7 mg of the mixture of **11** and **12** was obtained from heterologous expression of pET28-ptetO-gfpv2::*pgrCDE*^{L96D}*F*. All compounds were isolated as colorless oils and all yields are reported from 9 L culture volume.

Overexpression and purification of recombinant proteins

For protein overexpression, the constructed pHis8-TEV-based plasmids were chemically transformed into *E. coli* BL21 (DE3). Liquid overnight pre-cultures of transformants were used to inoculate an expression culture (1% (v/v)) grown in LB medium supplemented with kanamycin at a final concentration of 50 μg/mL, which was then incubated at 37 °C, 180 rpm. After an OD_{600} of 0.6-0.8 was reached, cultures were pre-cooled to 4 °C before induction. Expression was induced by addition of isopropyl-β-D-thiogalactopyranoside (IPTG) with a final concentration of 0.1 mM and cultures were incubated for 18-20 hours at 16 °C, 180 rpm. Cell pellets were collected by centrifugation (6000 rpm, 10 min, 4 °C), washed with lysis buffer (50 mM Tris-Cl buffer, 500 mM NaCl, 10% (v/v) glycerol, pH 8.0), and stored at -20 °C until further purification.

Protein purification was performed using nickel-nitrilotriacetic acid (Ni-NTA) resin (Cube Biotech) following the instructions of the manufacturer with some modifications. Cell pellets derived from 1 L culture were resuspended in 30 mL lysis buffer and disrupted by ultrasonication (Branson Ultrasonics Sonifier, USA) on ice at 35% amplitude for 2.5 min total duration under a 1 s pulse-on/ 1 s pulse-off cycle. Cellular debris and insoluble proteins were removed by centrifugation (12,000 rpm, 30 min, 4 ºC) and the supernatant was further purified by Ni-NTA affinity chromatography. Ni-NTA resin was mixed with the supernatant and incubated on ice for 1 hour while shaking. The bound protein was eluted with buffer B (50 mM Tris-Cl buffer, 500 mM NaCl, and 500 mM imidazole, pH 8.0) after washing with 5 bed volumes of buffer A (50 mM Tris-Cl buffer, 500 mM NaCl, 10 mM imidazole, pH 8.0). The affinity purification results were analyzed by SDS-PAGE of eluted fractions and pure fractions were pooled. The purified proteins were subsequently desalted on PD-10 columns (GE Healthcare, USA) according to the instructions of the manufacturer. The buffer was then exchanged to storage buffer (50 mM HEPES, 150 mM NaCl, 10% glycerol, pH 8.0). The protein solution was concentrated using Vivaspin centrifugal concentrator (Sigma-Aldrich, MWCO 10 kDa) and protein concentrations were measured using the Pierce BCA Protein Assay Kit (Thermo Scientific, USA). The purified proteins were directly used in *in vitro* assays or stored at -80 ºC for further experiments.

Mutagenesis of the DDMPLG motif in PgrE and purification of PgrE mutants

The mutagenesis of the DDMPLG motif in PgrE was carried out using the respective designed primers with the pHis8-TEV::*pgrE* plasmid as a template. The utilized oligonucleotide primers are summarized in Supplementary Table S6. After PCR amplification using Q5 high-fidelity DNA polymerase, the obtained DNA fragments were purified and digested with DpnI (NEB). Further DNA assembly steps were carried out according to the protocol described above. The plasmids containing the correct mutations were used to be chemically transformed into *E. coli* BL21(DE3) for subsequent expression. The expression and purification of the PgrE mutant proteins were performed analogous to the wild type proteins and purified proteins were used for *in vitro* assays or stored at -80 °C in storage buffer as described above.

***In vitro* enzymatic assays**

Enzymatic assays for functional characterization of selected proteins were performed for individual MTs and TPs or in combination. Assays were performed in 50 mM HEPES buffer (pH 8.0), 0.4 mM FPP was finally added to 20 μM $MgCl_2$, 0.05 μM $MnCl_2$, 4 mM DTT, 1 mM SAM, and 2 μM enzyme in 250 μL reaction scale. Each assay was overlaid with 300 μL hexane and incubated at 30 °C for 3 h before quenching. The two-phase assay solution was vortexed for 15 seconds twice following sufficient standing to allow separation of the two phases. The hexane extract was collected and used directly for GC-EI-MS analysis.

For the assay depicted in Figure 4, the enzymatic assay conditions were set as described above. FPP was added and incubated for 3 h at 30 °C without overlaying with hexane. Subsequently, 10 U of alkaline phosphatase (AP) was added, followed by the addition of 300 μL of hexane and incubated for another 3 h to facilitate full conversion. After the reaction, the two-phase assay solution was quenched by vortexing twice, followed by standing or centrifugation to allow for phase separation. Unless otherwise stated, all assays characterizing individual enzymes and/or different precursor combinations were performed using the same general conditions and analyzed by GC-EI-MS analyses.

Pull-down assays with MT PgrF

The MT-encoding gene *pgrF* from the *pgr* operon was amplified using specific primers with *P. grimontii* DZM 17515 genomic DNA as the template and subsequently cloned into the modified vector pGS-21a-ΔHis8. After confirmation by sequencing, the plasmid was transformed into *E. coli* BL21(DE3) for expression in 1.5 L LB supplemented with 100 mg/mL ampicillin, followed by induction with 0.1 mM IPTG at 16 °C, 180 rpm for 16-18 hours. Harvested pellet was resuspended in buffer C (50 mM HEPES, 150 mM NaCl, 2 mM DTT, 10% glycerol, pH 8.0) and lysed by sonication. The soluble portion of the lysate was incubated with GSH-agarose beads at 4 °C for 1 hour and incubated on a shaker to bind the GST fusion protein to the beads. The mixture was then loaded onto an affinity column, the beads were aggregated by centrifugation (500 rpm, 4 °C, 5 min), and the flow-through was collected for SDS-PAGE analysis, taking care not to lose any beads. Unbound components were fully removed by adding at least 5 times the bed volume of buffer C. The bound GST-PgrF was eluted with buffer D (containing 50 mM reduced glutathione) and collected for SDS-PAGE analysis.

The prepared beads bound to GST-PgrF were used for pull-down assays with His8-PgrC or His8-PgrD, in addition to beads bound to GST-PgrF alone as a control. The supernatant obtained by sonication and centrifugation from protein overexpression in *E. coli* BL21(DE3) pHis8-TEV::*pgrC* or *E. coli* BL21(DE3) pHis8-TEV::*pgrD* in buffer C with EDTA-free Protease-Inhibitor-Cocktail (catalog no. 04693159001, Roche) was mixed with above freshly-prepared GSH-agarose beads bound to GST-PgrF. The GST beads were gently suspended to mix with the His_8 fusion protein solution and incubated with shaking at 4 °C for 2-3 hours. The mixture was then loaded onto the affinity chromatography column and, as with the treatment of the GST fusion protein alone, the unbound fraction was fully removed using at least 5 bed volumes of buffer C. The bound protein fractions were eluted with buffer D and the fraction was collected and visualized by SDS-PAGE. Experiments with pGS-21a-ΔHis8 empty vector and pGS-21a-ΔHis8::*pgrF* were used as controls and treated as above. 20 μL of collected fractions and 10 μL of sample buffer were added and heated to 100 °C for 5 minutes in a water bath. Thereafter, the samples were centrifuged briefly. Half the

amount (15 µL) of supernatant was analyzed by SDS PAGE. The protein bands were visualized by Coomassie blue staining.

Synthesis of farnesyl pyrophosphate

Farnesyl chloride

In a 100 mL Schlenk flask, 2.8 mL farnesol (2.5 g, 11.2 mmol, 1.0 eq.; mixture of isomers) was dissolved in DMF (40 mL), and 1.64 mL *s*-collidine (1.49 g, 12.3 mmol, 1.1 eq.) and 1.42 g LiCl (33.6 mmol, 3.0 eq.) were added. The mixture was cooled to 0 °C and 0.95 mL MsCl (1.41 g, 12.3 mmol, 1.1 eq.) was added dropwise and the mixture was allowed to warm to room temperature. After stirring for 18 hours, the solution was poured into ice water (100 mL) and extracted with pentane (2 × 50 mL). Combined organic extracts were washed with saturated $CuSO_4$ solution (20 mL), water (50 mL) and brine (50 mL), dried over Na_2SO_4, filtered, and the solvent removed under reduced pressure to yield the product as a yellow oil (2.50 g, 10.4 mmol, 93%), which was used without further purification. The analytical data is in agreement with data reported in the literature.[3]

^{1}H-NMR (600 MHz, $CDCl_3$): δ [ppm] = 5.47-5.42 (m, 1H), 5.14-5.06 (m, 2H), 4.10 (d, J = 8.1 Hz, 1.7H), 4.08 (d, J = 8.1 Hz, 0.3H), 2.14-2.09 (m, 2.1H), 2.09-2.02 (m, 4.6H)*, 2.00-1.96 (m, 1.5H), 1.73 (d, J = 1.5 Hz, 1.8H), 1.72 (d, J = 1.5 Hz, 1H), 1.69 (d, J = 1.4 Hz, 1.5H)*, 1.68 (d, J = 1.4 Hz, 1.5H)*, 1.61 (bs, 1.5H), 1.60 (bs, 4.3H). *Signals overlay with uncharacterized impurity. ^{13}C {^{1}H}-NMR (151 MHz, $CDCl_3$): δ [ppm] = 142.9, 135.8, 131.5, 124.5, 123.6, 120.4, 41.3, 39.8, 39.6, 26.8, 26.3, 25.9, 17.8, 16.3, 16.2.

Farnesyl pyrophosphate (4a)

In a 10 mL Schlenk tube, 450 mg Tris(tetrabutylammonium) hydrogen pyrophosphate (499 µmol, 1.2 eq.) was dissolved in 0.9 mL ACN, and 100 mg farnesyl chloride (415 µmol, 1.0 eq.) was added dropwise. The mixture was stirred at room temperature for 4 hours and the volatiles removed under reduced pressure. The residue was suspended in ion exchange buffer (25 mM $(NH_4)_2CO_3$, 2 vol-% ^{i}PrOH) and applied to a 1.5 × 5.5 cm column with DOWEX 50 WX 8 ion exchange resin (H^+ form), which was equilibrated by flushing with 3 column volumes 1 M $(NH_4)_2SO_4$ and washed by flushing with 3 column volumes of ion exchange buffer. The sample was eluted by 2 column volumes of ion exchange buffer. The used flow rate was 0.67 mL/min. The eluted sample (slightly yellowish, opaque liquid) was freeze-dried, yielding 170 mg of a yellowish solid. This material was suspended in a small amount of 50 mM $(NH_4)_2CO_3$ (< 1 mL) in a 50 mL conical centrifuge tube and 4 mL of ^{i}PrOH/ACN was added. A white precipitate formed and the suspension was cleared by centrifugation (5 min, 2300 × *g*). The supernatant was transferred to a flask with a pipette and the procedure was repeated two times. Combined extracts were concentrated under reduced pressure and the residue diluted with 2 mL of water. Freeze-drying gave 119 mg of a pale yellow solid (275 µmol, 66%). The corresponding analytical data are in agreement with data reported in the literature.[4,5]

^{1}H-NMR (600 MHz, D_2O): δ [ppm] = 5.48-5.44 (m, 1H), 5.25-5.16 (m, 2H), 4.47 (t, J = 6.6 Hz, 2H), 2.19-2.13 (m, 2H), 2.12-2.07 (m, 4H), 2.05-2.00 (m, 2H), 1.72 (bs, 2.4H), 1.70-1.67 (m, 3.6H), 1.64 1.60 (m, 5.8H). ^{13}C{^{1}H}-NMR (151 MHz, D_2O): δ [ppm] = 142.9, 136.6, 133.4, 124.4, 124.2, 119.7, 62.7, 38.80, 38.77, 25.7, 25.6, 24.8, 16.9, 15.6, 15.2. ^{31}P {^{1}H}-NMR (243 MHz, D_2O): δ [ppm] = -9.0 (bd, J = 21.8 Hz, 1P), -10.6 (d, J = 21.8 Hz, 1P).

HR-MS (ESI–): $C_{15}H_{27}O_7P_2$; calcd. *m/z* 381.1237 [M-H]$^-$, obsd. *m/z* 381.1241[M-H]$^-$

General procedures for compound characterization

GC-MS measurements

Gas chromatography electron ionization mass spectrometry (GC-EI-MS) analysis was performed using an Agilent 7890A gas chromatograph coupled to an HP7683 automatic liquid sampler and a 5975C mass spectrometer equipped with a HP-5ms capillary column (30 m x 0.25 mm inner diameter x 0.25 μm film thickness, Agilent). Helium was used as carrier gas at a constant flow rate of 1.1 mL/min, and 1 μL of sample was injected using pulse split (1:5) mode. The inlet temperature was set to 250 °C and the actual oven temperature achieved varied depending on the method settings. For samples dissolved in pentane or hexane, the initial temperature was at 40 °C (2 min hold) and elevated to 280 °C (5 min hold) at a rate of 10 °C/min, while the sample dissolved in deuterated benzene was diluted with pentane (1:1000), the corresponding oven temperature program started at 40 °C, elevated with 10 °C/min to 280 °C (5 min hold). MS in SIM mode sets solvent delay 8 min. Samples in deuterated chloroform were diluted with pentane (1:1000) and the oven temperature program started at 63 °C (8 min hold), elevated with 7 °C/min to 280 °C (5 min hold), and MS in SIM mode sets solvent delay 5 min. The mass spectrometer was operated with electron impact ionization (70 eV) in scan mode (40 to 380 amu, threshold 40 amu). Data analysis was performed using GC-MS Chemstation Quantitative Analysis (version 7.0, Agilent), and data visualization was performed with OriginPro (version 9.0.0, OriginLab Corporation).

HR-MS measurement

High-resolution mass spectrometry (HRMS) analysis for farnesyl pyrophosphate (4a) was performed on a Bruker Elute UPLC instrument coupled to Bruker impact II High performance mass spectrometer with Quadrupole Time-of-Flight (Q-TOF) mass analyzer using Electrospray Ionization (ESI) Source (Apollo II) operated under negative ion mode. APCI-HRMS data for terpene products were acquired on an Agilent 6538 Q-TOF mass spectrometer utilizing an atmospheric pressure chemical ionization (APCI) source in positive ionization mode. See Supplementary Figure S63 for detailed spectra.

NMR measurements

Nuclear Magnetic Resonance (NMR) spectra were recorded on Bruker AVANCE II 300, AVANCE neo 400, ASCEND III 600 or AVANCE neo 600 spectrometers at ambient temperature. The chemical shifts are given in *δ*-values (ppm) relative to TMS (^{1}H, ^{13}C) or H_3PO_4 (^{31}P). ^{1}H and ^{13}C spectra were referenced internally using the residual solvent resonances ($CDCl_3$: δ_H = 7.26 ppm, δ_C = 77.16 ppm; D_2O: δ_H = 4.79 ppm; C_6D_6: δ_H = 7.16 ppm, δ_C = 128.06 ppm). ^{31}P chemical shifts were referenced by unified chemical shift scale.[6] The coupling constants *J* are given in Hertz [Hz] and determined assuming first-order spin-spin coupling. The following abbreviations were used for the allocation of signal multiplicities: s - singlet, bs - broad singlet, d - doublet, bd - broad doublet, t - triplet, qnt - quintet, m - multiplet, or any combination thereof. Structure elucidation was achieved by analysis of 1D-NMR (^{1}H, $^{13}C\{^{1}H\}$, DEPT135) and 2D-NMR (COSY, HSQC, HMBC, H2BC, NOESY) spectra. Data visualization was performed with MestReNova (version 6.1.0, Mestrelab Research) and TopSpin (version 4.5.0, Bruker BioSpin).

Protein structure modeling

The predicted protein structure models of PgrE and the selected TSs were generated using AlphaFold3[7] (https://alphafoldserver.com/) by uploading the corresponding amino acid sequences and using the default setting. The generated predicted structures were uploaded to the PDBsum Generate server[8] (https://www.ebi.ac.uk/-thornton-srv/databases/pdbsum/-Generate.html) for confidence coefficient verification and protein secondary structure analysis. The amino acid sequences after mutation of the active-site motif of PgrE were individually uploaded to AlphaFold3 for protein structure prediction. All selected protein structures were visualized using the PyMOL program[9] (https://www.pymol.org/). The prediction of protein heterodimer structures of MTs PgrF and PgrC or PgrD was performed by uploading protein sequences to the GalaxyHeteromer web server[10] (https://galaxy.seoklab.org/index.html) and the input sequences were untagged. From the independent predictions, the model that showed the highest combined reliability based on its overall confidence score and consistency within the top-ranking structural cluster was selected and the interaction interfaces were visualized by using the InterfaceResidues script (https://pymol-wiki.org/index.php/InterfaceResidues) in PyMOL to find the interface residues between the two proteins in the complex.

Table S1. Terpene biosynthetic gene clusters identified from *Pseudomonas* sp. genome database.

Species	RefSeq ID	Accession ID	Options/Filter
Pseudomonas grimontii DSM 17515	GCF_007858185.1	WP_090402647	terpene cyclase
Pseudomonas grimontii BS2976	GCF_900101085.1	WP_090402647	terpene cyclase
Pseudomonas chlororaphis Lzh-T5	GCF_002844145.1	WP_101282461	terpene cyclase
Pseudomonas fuscovaginae UPB0736	GCF_024585345.2	WP_010449696	terpene synthase
Pseudomonas asplenii ATCC 23835	GCF_900105475.1	WP_090206687	terpene synthase
Pseudomonas sp. 286	GCF_900581295.1	WP_122577304	terpene synthase
Pseudomonas atacamensis SM1	GCF_017167965.1	WP_206420717	terpene synthase
Pseudomonas chlororaphis subsp. *aurantiaca* CW2	GCF_003851225.1	WP_124299460	terpene synthase
Pseudomonas synxantha 30B	GCF_003851465.1	WP_124376239	terpene synthase
Pseudomonas syringae PA-4-7F	GCF_021606685.1	WP_236418672	terpene synthase
Pseudomonas fuscovaginae IRRI 6609	GCF_001293465.1	WP_054063904	terpene synthase
Pseudomonas asplenii B21-058	GCF_026016305.1	WP_265085601	terpene synthase
Pseudomonas sp. FW300-N1A1	GCF_002901565.1	WP_103400897	terpene synthase
Pseudomonas sp. 286	GCF_900590465.1	WP_122722183	terpene synthase
Pseudomonas baetica 25P2F9	GCF_019733355.1	WP_221733304	terpene synthase
Pseudomonas chlororaphis subsp. *chlororaphis* DSM 50083	GCF_003945765.1	WP_125738087	terpene synthase
Pseudomonas chlororaphis subsp. *chlororaphis* DSM 50083	GCF_007858335.1	WP_125738087	terpene synthase
Pseudomonas chlororaphis ATCC 17414	GCF_028747925.1	WP_125738087	terpene synthase
Pseudomonas agarici NCPPB 2472	GCF_001543125.1	WP_060782784	terpene synthase
Pseudomonas chlororaphis DSM 50083	GCF_016803445.1	WP_125738087	terpene synthase
Pseudomonas chlororaphis PcR3-3(2)	GCF_030388745.1	WP_273865072	terpene synthase
Pseudomonas sp. GM50	GCF_000282375.1	WP_008013502	terpene synthase
Pseudomonas chlororaphis Lzh-T5	GCF_002844145.1	WP_101282458	terpene synthase
Pseudomonas sp. 286	GCF_900590725.1	WP_122577304	terpene synthase
Pseudomonas fluorescens EK007-7t-asp	GCF_001931665.1	WP_073523146	terpene synthase
Pseudomonas sp. 286	GCF_900576665.1	WP_122557629	terpene synthase
Pseudomonas chlororaphis subsp. aurantiaca zm-1	GCF_010092525.1	WP_162095310	terpene synthase
Pseudomonas chlororaphis ATCC 17814	GCF_028747465.1	WP_125738087	terpene synthase
Pseudomonas sp. OV341	GCF_004368865.1	WP_134103164	terpene synthase
Pseudomonas syringae PA-6-9A	GCF_021605315.1	WP_236418672	terpene synthase
Pseudomonas asplenii IPG72	GCF_028616935.1	WP_273824123	terpene synthase
Pseudomonas sp. GM102	GCF_000282555.1	WP_007907633	terpene synthase
Pseudomonas chlororaphis subsp. *aurantiaca* StFRB508	GCF_002355875.1	WP_096374226	terpene synthase
Pseudomonas sp. RW409	GCF_003184125.1	WP_110177179	terpene synthase
Pseudomonas sp. 286	GCF_900581175.1	WP_122427640	terpene synthase
Pseudomonas agarici IPO3739	GCF_013385335.1	WP_248672820	terpene synthase

Species	RefSeq ID	Accession ID	Options/Filter
Pseudomonas chlororaphis ATCC 9446	GCF_028747405.1	WP_125738087	terpene synthase
Pseudomonas brassicacearum 48H11	GCF_003732225.1	WP_123426621	terpene synthase
Pseudomonas syringae PA-6-3H	GCF_021605805.1	WP_236418672	terpene synthase
Pseudomonas fuscovaginae UPB0736 isolate E124	GCF_000251185.1	WP_010449696	terpene synthase
Pseudomonas grimontii BS2976	GCF_900101085.1	WP_090408242	terpene synthase
Pseudomonas chlororaphis CGMCC 1.1793	GCF_030505175.1	WP_125738087	terpene synthase
Pseudomonas asplenii ATCC 23835	GCF_900105475.1	WP_090210520	terpene synthase
Pseudomonas sp. 286	GCF_900583255.1	WP_122577304	terpene synthase
Pseudomonas zeae OE 48.2	GCF_014268485.2	WP_217827617	terpene synthase
Pseudomonas chlororaphis subsp. aureofaciens 66	GCF_003851405.1	WP_103331240	terpene synthase
Pseudomonas grimontii DSM 17515	GCF_007858185.1	WP_090408242	terpene synthase
Pseudomonas moraviensis OTU5BARRA1	GCF_024072135.1	WP_252874173	terpene synthase
Pseudomonas fuscovaginae IRRI 7007	GCF_001293475.1	WP_054056865	terpene synthase
Pseudomonas mandelii 29	GCF_024138395.1	WP_253544710	terpene synthase
Pseudomonas asplenii B21-058	GCF_026016305.1	WP_265084422	terpene synthase
Pseudomonas chlororaphis HT66	GCF_000597925.1	WP_025804375	terpene synthase
Pseudomonas sp. MYb60	GCF_002979555.1	WP_105522479	terpene synthase
Pseudomonas sp. 286	GCF_900590695.1	WP_122722183	terpene synthase
Pseudomonas sp. 25 R 14	GCF_900004755.1	WP_065943924	terpene synthase
Pseudomonas chlororaphis subsp. chlororaphis	GCF_900625015.1	WP_125738087	terpene synthase
Pseudomonas chlororaphis subsp. aurantiaca zm-1	GCF_010092525.1	WP_101282458	terpene synthase
Pseudomonas zeae BIM B-582	GCF_024610595.1	WP_257358031	terpene synthase
Pseudomonas syringae PA-6-8G	GCF_021605305.1	WP_236418672	terpene synthase
Pseudomonas chlororaphis ATCC 9447	GCF_029536045.1	WP_125738087	terpene synthase
Pseudomonas sp. GM18	GCF_000282195.1	WP_007933138	terpene synthase
Pseudomonas sp. GW531-T4	GCF_002901665.1	WP_103331240	terpene synthase
Pseudomonas sp. 286	GCF_900601395.1	WP_122722183	terpene synthase
Pseudomonas fluorescens EK007-RG4	GCF_001902145.1	WP_073523146	terpene synthase
Pseudomonas sp. 286	GCF_900580655.1	WP_122427640	terpene synthase
Pseudomonas agarici IPO3740	GCF_013385305.1	WP_248672820	terpene synthase
Pseudomonas chlororaphis ATCC 9446	GCF_900183055.1	WP_125738087	terpene synthase
Pseudomonas kairouanensis KC12	GCF_004682055.1	WP_135288510	terpene synthase
Pseudomonas syringae PA-6-9D	GCF_021605335.1	WP_236418672	terpene synthase
Pseudomonas chlororaphis O6	GCF_000264555.1	WP_009051468	terpene synthase
Pseudomonas trivialis MYb75	GCF_002975295.1	WP_105522479	terpene synthase
Pseudomonas glycinae BML-PP039	GCF_021602405.1	WP_236196480	terpene synthase family protein
Pseudomonas gingeri D1001	GCF_013385725.1	WP_177064801	terpene synthase family protein
Pseudomonas sputi BML-PP014	GCF_021603585.1	WP_236182010	terpene synthase family protein
Pseudomonas gingeri IPO3776	GCF_013386815.1	WP_177064801	terpene synthase family protein
Pseudomonas koreensis B1M1-15	GCF_025447595.1	WP_262194564	terpene synthase family protein

Species	RefSeq ID	Accession ID	Options/Filter
Pseudomonas atagonensis PS14	GCF_011369485.1	WP_166219950	terpene synthase family protein
Pseudomonas proteolytica PA-6-4D	GCF_021605705.1	WP_092232649	terpene synthase family protein
Pseudomonas gingeri C3001	GCF_013385845.1	WP_177064801	terpene synthase family protein
Pseudomonas fluorescens 51484_Pf0-1_Pf0-2x_glnA-T1078G	GCF_019898665.1	WP_011333305	terpene synthase family protein
Pseudomonas gingeri IPO3737	GCF_013385355.1	WP_177057043	terpene synthase family protein
Pseudomonas gingeri IPO3769	GCF_013386895.1	WP_177056953	terpene synthase family protein
Pseudomonas marginalis MGMM3	GCF_029916865.1	WP_281112584	terpene synthase family protein
Pseudomonas mandelii 29	GCF_024138395.1	WP_253544709	terpene synthase family protein
Pseudomonas gingeri C1001	GCF_013385925.1	WP_177064801	terpene synthase family protein
Pseudomonas fluorescens 51486_Pf0-1_Pf0-2x_glnA-G1249C	GCF_019898705.1	WP_011333305	terpene synthase family protein
Pseudomonas gingeri E1001	GCF_013385575.1	WP_177057043	terpene synthase family protein
Pseudomonas gingeri IPO3767	GCF_013386915.1	WP_177057043	terpene synthase family protein
Pseudomonas chlororaphis PcR3-3(2)	GCF_030388745.1	WP_273865073	terpene synthase family protein
Pseudomonas glycinae MS586	GCF_001594225.2	WP_190241533	terpene synthase family protein
Pseudomonas fluorescens 29639_Pf0-1_Pf0-2x_ntrB-A46C	GCF_019898745.1	WP_011333305	terpene synthase family protein
Pseudomonas chlororaphis qlu-1	GCF_014524625.1	WP_101282458	terpene synthase family protein
Pseudomonas gingeri J4002	GCF_013386645.1	WP_177082823	terpene synthase family protein
Pseudomonas glycinae XJFL-1	GCF_019719375.1	WP_011333305	terpene synthase family protein
Pseudomonas gingeri D5001	GCF_013385685.1	WP_177064801	terpene synthase family protein
Pseudomonas gingeri P8018	GCF_013387125.1	WP_177057043	terpene synthase family protein
Pseudomonas gingeri IPO3777	GCF_013386805.1	WP_177064801	terpene synthase family protein
Pseudomonas koreensis WZ005	GCF_017939605.1	WP_210709313	terpene synthase family protein
Pseudomonas chlororaphis qlu-1	GCF_014524625.1	WP_101282462	terpene synthase family protein
Pseudomonas proteolytica PA-6-9F	GCF_021605255.1	WP_092232649	terpene synthase family protein
Pseudomonas gingeri C4002	GCF_013385835.1	WP_177064801	terpene synthase family protein
Pseudomonas fluorescens DR397	GCF_010448615.1	WP_163974473	terpene synthase family protein
Pseudomonas gingeri IPO3769	GCF_013386895.1	WP_177057043	terpene synthase family protein
Pseudomonas proteolytica 1912-L	GCF_029269195.1	WP_092232649	terpene synthase family protein
Pseudomonas gingeri IPO3738	GCF_013385325.1	WP_146049213	terpene synthase family protein
Pseudomonas carnis PA-1-5B	GCF_021887455.1	WP_237506360	terpene synthase family protein
Pseudomonas gingeri C2001	GCF_013385905.1	WP_177131578	terpene synthase family protein
Pseudomonas fluorescens 51485_Pf0-1_Pf0-2x_glnA-C5A	GCF_019898675.1	WP_011333305	terpene synthase family protein
Pseudomonas gingeri E1001	GCF_013385575.1	WP_177056953	terpene synthase family protein
Pseudomonas gingeri IPO3757	GCF_013386905.1	WP_177057043	terpene synthase family protein
Pseudomonas fitomaticsae FIT81	GCF_021018765.1	WP_230736619	terpene synthase family protein
Pseudomonas agarici IPO3740	GCF_013385305.1	WP_017133849	terpene synthase family protein
Pseudomonas fluorescens 51487_Pf0-1_Pf0-2x_glnA-T1078C	GCF_019898725.1	WP_011333305	terpene synthase family protein

Species	RefSeq ID	Accession ID	Options/Filter
Pseudomonas chlororaphis subsp. *aurantiaca* YS21	GCF_016757635.1	WP_124299460	terpene synthase family protein
Pseudomonas gingeri A6001	GCF_013386115.1	WP_177082823	terpene synthase family protein
Pseudomonas glycinae PSB00018	GCF_016009155.1	WP_197869477	terpene synthase family protein
Pseudomonas gingeri D8001	GCF_013385635.1	WP_177064801	terpene synthase family protein
Pseudomonas gingeri IPO3756	GCF_013386965.1	WP_177057043	terpene synthase family protein
Pseudomonas fluorescens 29636_Pf0-1_Pf0-2x_glnA-A938CG943A	GCF_019898805.1	WP_011333305	terpene synthase family protein
Pseudomonas chlororaphis qlu-1	GCF_014524625.1	WP_101282461	terpene synthase family protein
Pseudomonas gingeri J1002	GCF_013386725.1	WP_177082823	terpene synthase family protein

Table S2. Methyltransferase genes in the *Pseudomonas* genome DB.

Protein ID	Gene	Putative function	Organism	Group (in this study)
WP_198560294.1	HLB40_RS14745	class I SAM-dependent methyltransferase	*Pseudomonas chlororaphis* qlu-1	I
WP_101284138.1	CXP47_RS14860	class I SAM-dependent methyltransferase	*Pseudomonas chlororaphis* Lzh-T5	I
WP_162095311.1	GSF17_RS15435	class I SAM-dependent methyltransferase	*Pseudomonas chlororaphis* subsp. *aurantiaca* zm-1	I
WP_096376005.1	PCAU_RS14795	class I SAM-dependent methyltransferase	*Pseudomonas chlororaphis* StFRB508	I
WP_009051472.1	PchlO6_6045	methyltransferase type 12	*Pseudomonas chlororaphis* O6	I
WP_025804372.1	M217_RS0102670	methyltransferase type 12	*Pseudomonas chlororaphis* HT66	I
WP_253491185.1	QGQ83_RS27230	class I SAM-dependent methyltransferase	*Pseudomonas marginalis* MGMM3	I
WP_007933134.1	PMI21_00067	methyltransferase type 12	*Pseudomonas* sp. GM18	I
WP_007907637.1	PMI18_05946	methyltransferase type 12	*Pseudomonas* sp. GM102	I
WP_008013508.1	PMI30_05086	methyltransferase type 12	*Pseudomonas* sp. GM50	I
WP_090408237.1	BLQ48_RS29165	class I SAM-dependent methyltransferase	*Pseudomonas grimontii* BS2976	I
WP_146371476.1	FIV39_RS03785	methyltransferase domain-containing protein	*Pseudomonas grimontii* DSM 17515	I
WP_281112585.1	QGQ83_RS27215	methyltransferase domain-containing protein	*Pseudomonas marginalis* MGMM3	II
WP_090408240.1	FIV39_RS03780	class I SAM-dependent methyltransferase	*Pseudomonas grimontii* DSM 17515	II
WP_090408240.1	BLQ48_RS29170	class I SAM-dependent methyltransferase	*Pseudomonas grimontii* BS2976	II
WP_007933137.1	PMI21_00070	Methyltransferase	*Pseudomonas* sp. GM18	II
WP_008013504.1	PMI30_05084	Methyltransferase	*Pseudomonas* sp. GM50	II

WP_007907635.1	PMI18_05944	Methyltransferase	*Pseudomonas* sp. GM102	II
WP_101282459.1	CXP47_RS14835	class I SAM-dependent methyltransferase	*Pseudomonas chlororaphis* Lzh-T5	II
WP_101282459.1	HLB40_RS14720	class I SAM-dependent methyltransferase	*Pseudomonas chlororaphis* qlu-1	II
WP_162095308.1	GSF17_RS15415	methyltransferase domain-containing protein	*Pseudomonas chlororaphis* subsp. *aurantiaca* zm-1	II
WP_025804374.1	M217_RS0102680	Methyltransferase	*Pseudomonas chlororaphis* HT66	II
WP_096374227.1	PCAU_RS14775	methyltransferase domain-containing protein	*Pseudomonas chlororaphis* subsp. *aurantiaca* StFRB508	II
WP_009051469.1	PchlO6_6042	Methyltransferase	*Pseudomonas chlororaphis* O6	II
WP_090408245.1	BLQ48_RS29180	SAM-dependent methyltransferase	*Pseudomonas grimontii* BS2976	III
WP_090408245.1	FIV39_RS03770	SAM-dependent methyltransferase	*Pseudomonas grimontii* DSM 17515	III

Table S3. Terpene synthase genes in the *Pseudomonas* genome DB.

Protein ID	Gene	Putative function	Organism	Group (in this study)
WP_096374228.1	PCAU_RS14785	terpene synthase family protein	*Pseudomonas chlororaphis* subsp. *aurantiaca* StFRB508	A
WP_101282462.1	CXP47_RS14850	terpene synthase family protein	*Pseudomonas chlororaphis* Lzh-T5	A
WP_101282462.1	HLB40_RS14735	terpene synthase family protein	*Pseudomonas chlororaphis* qlu-1	A
WP_281112584.1	QGQ83_RS27210	terpene synthase family protein	*Pseudomonas marginalis* MGMM3	B
WP_025804375.1	M217_RS0102685	terpene synthase	*Pseudomonas chlororaphis* HT66	B
WP_007933138.1	PMI21_00071	terpene synthase	*Pseudomonas* sp. GM18	B
WP_090408242.1	BLQ48_RS29175	terpene synthase	*Pseudomonas grimontii* BS2976	B
WP_090408242.1	FIV39_RS03775	terpene synthase	*Pseudomonas grimontii* DSM 17515	B
WP_008013502.1	PMI30_05083	terpene synthase	*Pseudomonas* sp. GM50	B
WP_007907633.1	PMI18_05943	terpene synthase	*Pseudomonas* sp. GM102	B
WP_101282458.1	HLB40_RS14715	terpene synthase family protein	*Pseudomonas chlororaphis* qlu-1	B
WP_101282458.1	GSF17_RS15410	terpene synthase	*Pseudomonas chlororaphis* subsp. *aurantiaca* zm-1	B
WP_101282458.1	CXP47_RS14830	terpene synthase	*Pseudomonas chlororaphis* Lzh-T5	B
WP_096374226.1	PCAU_RS14770	terpene synthase	*Pseudomonas chlororaphis* subsp. *aurantiaca* StFRB508	B
WP_009051468.1	PchlO6_6041	terpene synthase	*Pseudomonas chlororaphis* O6	B

WP_096374229.1	PCAU_RS14790	terpene synthase family protein	*Pseudomonas chlororaphis* subsp. *aurantiaca* StFRB508	C
WP_101282463.1	HLB40_RS14740	terpene synthase family protein	*Pseudomonas chlororaphis* qlu-1	C
WP_101282463.1	CXP47_RS14855	terpene synthase family protein	*Pseudomonas chlororaphis* Lzh-T5	C
WP_009051471.1	PchlO6_6044	terpene synthase family protein	*Pseudomonas chlororaphis* O6	D
WP_101282461.1	HLB40_RS14730	terpene synthase family protein	*Pseudomonas chlororaphis* qlu-1	D
WP_101282461.1	CXP47_RS14845	terpene cyclase	*Pseudomonas chlororaphis* Lzh-T5	D
WP_162095310.1	GSF17_RS15430	terpene synthase	*Pseudomonas chlororaphis* subsp. *aurantiaca* zm-1	E

Table S4. Bioinformatic annotation of genes in the *pgr* pathway from *Pseudomonas grimontii* DSM 17515

Protein ID	Gene	Size (bp)	Putative function	Identity to corresponding gene in *sod* gene cluster	Identity to corresponding gene in *pchlO6* gene cluster
WP_090408231.1	*pgrA*	564	isopentenyl-diphosphate Delta-isomerase	52.27% to SodA	62.03% to PchlO6_6047
WP_090408234.1	*pgrB*	1833	1-deoxy-D-xylulose-5-phosphate synthase	64.09% to SodB	75.08% to PchlO6_6046
WP_146371476.1	*pgrC*	942	methyltransferase domain-containing protein	61.11% to SodC	81.41% to PchlO6_6045
WP_090408240.1	*pgrD*	921	class I SAM-dependent methyltransferase	44.70% to SodC	90.46% to PchlO6_6042
WP_090408242.1	*pgrE*	1029	terpene synthase	22.51% to SodD	88.50% to PchlO6_6041
WP_090408245.1	*pgrF*	1218	SAM-dependent methyltransferase	18.66 % to SodC	15.92% to PchlO6_6045

Table S5. Bioinformatics annotations of PgrF homologs in selected *Pseudomonas* species.

Homolog	RefSeq ID	Host strain	Genomic location (nt)	Nearest terpene BGC location (nt)	In terpene BGC?	Identity to pgrF gene
PgrF	WP_090408245.1	*P. grimontii* DSM 17515	Within *pgr* cluster	0	Yes	100 %
BLQ48_RS29180	WP_090408245.1	*Pseudomonas grimontii* BS2976	6,126,561 - 6,127,778	0	Yes	100%
PcchF (C4K27_RS06760)	WP_053259895.1	*Pseudomonas chlororaphis* subsp. *chlororaphis* DSM 50083	1,504,265 - 1,505,482	4,718,636 - 4,738,572	No	86.6%
C4K13_RS06940	WP_041988785.1	*Pseudomonas chlororaphis* subsp. *aureofaciens* DSM 6698	1,520,560 - 1,521,777	3,384,256 - 3,409,857	No	86.4%
QGQ83_RS27950	WP_281112651.1	*Pseudomonas marginalis* MGMM3	6,077,081 - 6,078,298	5,913,775 - 5,933,302	No	97.5%
PMI30_03552	WP_008010838.1	*Pseudomonas* sp. GM50	7,722 - 8,936	1,885 - 21,494	Yes	90.6%
PMI21_01439	WP_007935760.1	*Pseudomonas* sp. GM18	38,904 - 40,130	106,669 - 124,104	No	90.3%
PMI18_04265	WP_007903762.1	*Pseudomonas* sp. GM102	6,619 - 7,833	15,309 - 34,123	No	90.3%
PCAU_RS06015	WP_081362479.1	*Pseudomonas chlororaphis* subsp. *aurantiaca* StFRB508	1,322,441 - 1,323,658	3,194,377 - 3,223,260	No	86.7%
GSF17_RS06720	WP_016702088.1	*Pseudomonas chlororaphis* subsp. *aurantiaca* zm-1	1,493,572 -1,494,789	3,363,535 - 3,384,548	No	86.4%
PchlO6_1426	WP_009047435.1	*Pseudomonas chlororaphis* O6	1,554,945 - 1,556,162	6,622,143 - 6,643,162	No	86.9%
M217_RS0115985	WP_025806786.1	*Pseudomonas chlororaphis* HT66	310,484 - 311,701	232,847 - 245,130	No	85.9%
CXP47_RS06775	WP_041988785.1	*Pseudomonas chlororaphis* Lzh-T5	1,489,641 - 1,490,858	3,283,767 - 3,310,652	No	86.4%
HLB40_RS06715	WP_041988785.1	*Pseudomonas chlororaphis* qlu-1	1,489,683 - 1,490,900	3,284,148 - 3,311,033	No	86.4%

Table S6. Primers used in this study.

Primer name	Sequence (5'-3')	Description
spec-ptet-Rev	GGTCGATCCTCTTCTCTATC	Amplification of pET28-ptetO-GFPv2 vector backbone for DiPaC
spec-ptet-For	CATGGTTAGCAAAGGTGAAG	Amplification of pET28-ptetO-GFPv2 vector backbone for DiPaC
pHis8-TEV-R	TCCCTGAAAATAAAGATTCTCAC	Amplification of pHis8-TEV vector backbone for DiPaC
pHis8-TEV-F	GGATCCGAATTCGAGCTCC	Amplification of pHis8-TEV vector backbone for DiPaC
pET28-ptetO-pgrA-F	GTGATAGAGAAGAGGATCGACCATGGAAGAGTTACTGATTCTGG	Amplification of *pgrA-E* for generating plasmid pET28b-ptetO::*pgrABCDE*
pET28-ptetO-pgrC-F	GTGATAGAGAAGAGGATCGACCATGAGCACACAAGCGAAACAG	Amplification of *pgrC-F* or *pgrC* for generating plasmid pET28b-ptetO::*pgrCDEF*,pET28b-ptetO::*pgrCDE*, pET28b-ptetO::*pgrCD*, pET28b-ptetO*::pgrCDF*, pET28b-ptetO::pgrCE and pET28b-ptetO::pgrCEF
pET28-ptetO-pgrC-R	GTTCTTCACCTTTGCTAACCATGTTACGTGGCGTTGCGG	Amplification of *pgrC* for generating plasmid pET28b-ptetO::*pgrC*
pET28-ptetO-pgrCF-F	TGCCCGCAACGCCACGTAAATGTCCGTTACCGCTCAACCTGG	Amplification of *pgrF* for generating plasmid pET28b-ptetO::*pgrCF*
pET28-ptetO-pgrCF-V-R	TGCCCGCAACGCCACGTAA	Linearization of plasmid for generating plasmid pET28b-ptetO::*pgrCF*
pET28-ptetO-pgrCE-F	CAACGCCACGTAATGTGAGGAGCGAACCATGAACC	Amplification of *pgrE* for generating plasmid pET28b-ptetO::*pgrCE*
pET28-ptetO-pgrCE-V-R	TCGCTCCTCACATTACGTGGCGTTGCGGGCAA	Linearization of plasmid for generating plasmid pET28b-ptetO::*pgrCE*
pET28-ptetO-pgrD-F	GTGATAGAGAAGAGGATCGACCATGCCAGCACACGAGAGT	Amplification of *pgrD* for generating plasmid pET28b-ptetO::*pgrD* or pET28b-ptetO::*pgrDEF*, pET28b-ptetO*::pgrDE and* pET28b-ptetO*::pgrDF*
pET28-ptetO-pgrD-R	GTTCTTCACCTTTGCTAACCATGCTAGCGTTTGCACGCGATG	Amplification of *pgrD* for generating plasmid pET28b-ptetO::*pgrCD* and pET28b-ptetO*::pgrD*
pET28-ptetO-pgrDF-F	CATCGCGTGCAAACGCTAGATGTCCGTTACCGCTCAACCTGG	Amplification of *pgrF* for generating plasmid pET28b-ptetO::*pgrDF*
pET28-ptetO-pgrD-V-R	CTAGCGTTTGCACGCGATG	Linearization of plasmid for generating plasmid pET28b-ptetO::*pgrDF*
pET28-ptetO-pgrE-F	GTGATAGAGAAGAGGATCGACCATGAACCATTCTGCACAAG	Amplification of *pgrE* for generating plasmid pET28b-ptetO::*pgrEF*, pET28b-ptetO*::pgrE*

Primer name	Sequence (5'-3')	Description
pET28-ptetO-pgrE-R	GTTCTTCACCTTTGCTAACCATGCTACTCGATTCCGGGGTTG	Amplification of *pgrE* for generating plasmid pET28b-ptetO::*pgrABCDE*, pET28b-ptetO::*pgrCDE*, pET28b-ptetO::*pgrCE*, pET28b-ptetO::*pgrDE* and pET28b-ptetO::*pgrE*
pET28-ptetO-pgrEF-F	CAACCCCGGAATCGAGTAGATGTCCGTTACCGCTCAACC	Amplification of *pgrF* for generating plasmid pET28b-ptetO::*pgrEF*
pET28-ptetO-pgrE-V-R	CTACTCGATTCCGGGGTTG	Linearization of plasmid for generating plasmid pET28b-ptetO::*pgrEF*
pET28-ptetO-pgrF-R	GTTCTTCACCTTTGCTAACCATGTCAGGCCAGGTAACCATCCG	Amplification of *pgrC-F* or *pgrF* for generating plasmid pET28b-ptetO::*pgrCDEF*,pET28b-ptetO::*pgrCDF*, pET28b-ptetO::*pgrCF* and pET28b-ptetO::*pgrEF*
His-pgrC-F	GTGAGAATCTTTATTTTCAGGGAATGAGCACACAAGCGAAAC	Amplification of *pgrC* for generating plasmid pHis8-TEV::*pgrC*
His-pgrC-R	GACGGAGCTCGAATTCGGATCCTTACGTGGCGTTGCGGGCAAT	Amplification of *pgrC* for generating plasmid pHis8-TEV::*pgrC*
His-pgrD-F	GTGAGAATCTTTATTTTCAGGGAATGCCAGCACACGAGAGTA	Amplification of *pgrC* for generating plasmid pHis8-TEV::*pgrD*
His-pgrD-R	GACGGAGCTCGAATTCGGATCCCTAGCGTTTGCACGCGATG	Amplification of *pgrC* for generating plasmid pHis8-TEV::*pgrD*
His-pgrE-F	GTGAGAATCTTTATTTTCAGGGAATGAACCATTCTGCACAAG	Amplification of *pgrC* for generating plasmid pHis8-TEV::*pgrE*
His-pgrE-R	GACGGAGCTCGAATTCGGATCCCTACTCGATTCCGGGGTTG	Amplification of *pgrC* for generating plasmid pHis8-TEV::*pgrE*
His-pgrF-F	GTGAGAATCTTTATTTTCAGGGAATGTCCGTTACCGCTCAACC	Amplification of *pgrC* for generating plasmid pHis8-TEV::*pgrF*
His-pgrF-R	GACGGAGCTCGAATTCGGATCCTCAGGCCAGGTAACCATCCG	Amplification of *pgrC* for generating plasmid pHis8-TEV::*pgrF*
His-pcchB-F	GGTGAGAATCTTTATTTTCAGGGAATGAGTACGTTGACCTTGCC	Amplification of *pcchB* for generating plasmid pHis8-TEV::*pcchB*
His-pcchB-R	GTCGACGGAGCTCGAATTCGGATCCTCACTGCCGGGCCGGATCG	Amplification of *pcchB* for generating plasmid pHis8-TEV::*pcchB*
His-pcchC-F	GGTGAGAATCTTTATTTTCAGGGAATGAGCACTCAAGCCGCCAC	Amplification of *pcchC* for generating plasmid pHis8-TEV::*pcchC*
His-pcchC-R	GTCGACGGAGCTCGAATTCGGATCCTCATAGAGAGTCTCCCGGGC	Amplification of *pcchC* for generating plasmid pHis8-TEV::*pcchC*
His-pcchF-F	GAATCTTTATTTTCAGGGAATGTCTGCAACCGCAAC	Amplification of *pcchC* for generating plasmid pHis8-TEV::*pcchF*
His-pcchF-R	GGAGCTCGAATTCGGATCCTCAGCCCAGGTAGCC	Amplification of *pcchC* for generating plasmid pHis8-TEV::*pcchF*
pGS21a-Δhis-F	AGCAGCGGTATGTCCC	Amplification of pGS21a-Δhis vector backbone
pGS21a-Δhis-R	GGGACATACCGCTGCTAGAACCAGACATATGTATATCTCCTTC	Amplification of pGS21a-Δhis vector backbone

Primer name	Sequence (5'-3')	Description
GST-F	TCGGATCTGGGCCACACA	Linearization of plasmid for generating plasmid pGS21a-Δhis::*pgrF*
GST-R	TTTTGGAGGATGGTCGCC	Linearization of plasmid for generating plasmid pGS21a-Δhis::*pgrF*
GST-PgrF-F	GGCGACCATCCTCCAAAAATGTCCGTTACCGCTCAAC	Amplification of *pgrF* mutant for generating plasmid pGS21a-Δhis::*pgrF*
GST-PgrF-R	TGTGTGGCCCAGATCCGATCAGGCCAGGTAACCATC	Amplification of *pgrF* mutant for generating plasmid pGS21a-Δhis::*pgrF*
D92N-F	CCATCaatGATATGCCCCTG	Amplification of *pgrE* mutant for generating plasmid pHis8-TEV::*pgrE-D92N*
D92N-R	ATCattGATGGCCAGGACAAG	Amplification of *pgrE* mutant for generating plasmid pHis8-TEV::*pgrE-D92N*
D93N-F	TCGACaatATGCCCCTGGGC	Amplification of *pgrE* mutant for generating plasmid pHis8-TEV::*pgrE-D93N*
D93N-R	CATattGTCGATGGCCAGG	Amplification of *pgrE* mutant for generating plasmid pHis8-TEV::*pgrE-D93N*
D93E-F	TCGACgaaATGCCCCTGGGC	Amplification of *pgrE* mutant for generating plasmid pHis8-TEV::*pgrE-D93E*
D93E-R	CATttcGTCGATGGCCAGG	Amplification of *pgrE* mutant for generating plasmid pHis8-TEV::*pgrE-D93E*
D93S-F	TCGACagtATGCCCCTGGGC	Amplification of *pgrE* mutant for generating plasmid pHis8-TEV::*pgrE-D93S*
D93S-R	CATactGTCGATGGCCAGG	Amplification of *pgrE* mutant for generating plasmid pHis8-TEV::*pgrE-D93S*
D93L-F	TCGACctgATGCCCCTGGGC	Amplification of *pgrE* mutant for generating plasmid pHis8-TEV::*pgrE-D93L*
D93L-R	CATcagGTCGATGGCCAGG	Amplification of *pgrE* mutant for generating plasmid pHis8-TEV::*pgrE-D93L*
D93C-F	TCGACtgtATGCCCCTGGGC	Amplification of *pgrE* mutant for generating plasmid pHis8-TEV::*pgrE-D93C*
D93C-R	CATacaGTCGATGGCCAGG	Amplification of *pgrE* mutant for generating plasmid pHis8-TEV::*pgrE-D93C*
L96D-F	TGCCCgatGGCCGGCATG	Amplification of *pgrE* mutant for generating plasmid pHis8-TEV::*pgrE-L96D*
L96D-R	GCCatcGGGCATATCGTCG	Amplification of *pgrE* mutant for generating plasmid pHis8-TEV::*pgrE-L96D*
L96N-F	TGCCCaatGGCCGGCATGCG	Amplification of *pgrE* mutant for generating plasmid pHis8-TEV::*pgrE-L96N*
L96N-R	GGCCattGGGCATATCGTCG	Amplification of *pgrE* mutant for generating plasmid pHis8-TEV::*pgrE-L96N*
L96S-F	TGCCCagtGGCCGGCATGCG	Amplification of *pgrE* mutant for generating plasmid pHis8-TEV::*pgrE-L96S*
L96S-R	GGCCactGGGCATATCGTCG	Amplification of *pgrE* mutant for generating plasmid pHis8-TEV::*pgrE-L96S*
L96C-F	TGCCCtgtGGCCGGCATGCG	Amplification of *pgrE* mutant for generating plasmid pHis8-TEV::*pgrE-L96C*
L96C-R	GGCCacagGGCATATCGTCG	Amplification of *pgrE* mutant for generating plasmid pHis8-TEV::*pgrE-L96C*

Primer name	Sequence (5'-3')	Description
G97D-F	GCCCCTGgatCGGCATGCG	Amplification of *pgrE* mutant for generating plasmid pHis8-TEV::*pgrE-G97D*
G97D-R	CCGatcCAGGGGCATATCGTC	Amplification of *pgrE* mutant for generating plasmid pHis8-TEV::*pgrE-G97D*
D93NL96D-F	GACaatATGCCCgatGGCCGGCATGC	Amplification of *pgrE* mutant for generating plasmid pHis8-TEV::*pgrE-D93NL96D*
D93NL96D-R	CCatcGGGCATattGTCGATGGCCAGG	Amplification of *pgrE* mutant for generating plasmid pHis8-TEV::*pgrE-D93NL96D*

Homology arms are underlined.

Table S7. Bacterial strains used in this study.

Bacterial strains	Description	Source
E. coli DH5α	Used for all gene clones in this study	NEB
E. coli BL21(DE3)	Used for the expression of PgrF, PgrE and their mutant proteins	NEB
E. coli BAP1	Used for the heterologous expression of VOCs in this study	Pfeifer et al, 2001[11]
E. coli Δmtn (DE3)	Used for the overexpression of PgrC, PgrD	Liao et al, 2019 [12]
P. chlororaphis subsp. *chlororaphis* DSM 50083	Used for corresponding gDNA extraction	Mo et al, 2024 [2]
P. grimontii DSM 17515	Used for corresponding gDNA extraction and fermentation	DSMZ
E. coli BAP1::*pET28b-ptetO-GFPv2*	Used for expression of pET28b-ptetO-GFPv2 as control	Duell et al, 2019 [13]
E. coli BAP1::*pgrABCDEF*	Used for recombinant expression of pET28b-ptetO-GFPv2::*pgrABCDEF*	This study
E. coli BAP1::*pgrCDEF*	Used for recombinant expression of pET28b-ptetO-GFPv2::*pgrCDEF*	This study
E. coli BAP1::*pgrCDE*	Used for recombinant expression of pET28b-ptetO-GFPv2::*pgrCDE*	This study
E. coli BAP1::*pgrCDF*	Used for recombinant expression of pET28b-ptetO-GFPv2::*pgrCDF*	This study
E. coli BAP1::*pgrCD*	Used for recombinant expression of pET28b-ptetO-GFPv2::*pgrCD*	This study
E. coli BAP1::*pgrCEF*	Used for recombinant expression of pET28b-ptetO-GFPv2::*pgrCEF*	This study
E. coli BAP1::*pgrCE*	Used for recombinant expression of pET28b-ptetO-GFPv2::*pgrCE*	This study
E. coli BAP1::*pgrCF*	Used for recombinant expression of pET28b-ptetO-GFPv2::*pgrCF*	This study
E. coli BAP1::*pgrC*	Used for recombinant expression of pET28b-ptetO-GFPv2::*pgrC*	This study
E. coli BAP1::*pgrEF*	Used for recombinant expression of pET28b-ptetO-GFPv2::*pgrEF*	This study
E. coli BAP1::*pgrE*	Used for recombinant expression of pET28b-ptetO-GFPv2::*pgrE*	This study
E. coli BAP1::*pgrDE*	Used for recombinant expression of pET28b-ptetO-GFPv2::*pgrDE*	This study
E. coli BAP1::$pgrCDFE^{D93N}$	Used for recombinant expression of pET28b-ptetO-GFPv2::$pgrCDFE^{D93N}$	This study
E. coli Δmtn::*pgrC*	Used for recombinant expression of pHis8-TEV::*pgrC*	This study
E. coli Δmtn::*pgrD*	Used for recombinant expression of pHis8-TEV::*pgrD*	This study
E. coli BL21(DE3)::*pgrE*	Used for recombinant expression of pHis8-TEV::*pgrE*	This study

E. coli BL21(DE3)::*pgrF*	Used for recombinant expression of pHis8-TEV::*pgrF*	This study
E. coli Δmtn::*pcchB*	Used for recombinant expression of pHis8-TEV::*pcchB*	This study
E. coli Δmtn::*pcchC*	Used for recombinant expression of pHis8-TEV::*pcchC*	This study
E. coli BL21(DE3)::*pcchD*	Used for recombinant expression of pHis8-TEV::*pcchD*	This study
E. coli BL21(DE3)::*pGS21a-ΔHis8*	Used for recombinant expression of pGS21a-ΔHis8	This study
E. coli BL21(DE3)::*pgrF*	Used for recombinant expression of pGS21a-ΔHis::*pgrF*	This study
E. coli BL21(DE3)::$pgrE^{L96D}$	Used for recombinant expression of pHis8-TEV:: $pgrE^{L96D}$	This study
E. coli BL21(DE3)::$pgrE^{G97D}$	Used for recombinant expression of pHis8-TEV:: $pgrE^{G97D}$	This study
E. coli BL21(DE3)::$pgrE^{L96N}$	Used for recombinant expression of pHis8-TEV:: $pgrE^{L96N}$	This study
E. coli BL21(DE3)::$pgrE^{L96S}$	Used for recombinant expression of pHis8-TEV:: $pgrE^{L96S}$	This study
E. coli BL21(DE3)::$pgrE^{L96C}$	Used for recombinant expression of pHis8-TEV:: $pgrE^{L96C}$	This study
E. coli BL21(DE3)::$pgrE^{D92N}$	Used for recombinant expression of pHis8-TEV:: $pgrE^{D92N}$	This study
E. coli BL21(DE3)::$pgrE^{D93N}$	Used for recombinant expression of pHis8-TEV:: $pgrE^{D93N}$	This study
E. coli BL21(DE3)::$pgrE^{D93S}$	Used for recombinant expression of pHis8-TEV:: $pgrE^{D93S}$	This study
E. coli BL21(DE3)::$pgrE^{D93L}$	Used for recombinant expression of pHis8-TEV:: $pgrE^{D93L}$	This study
E. coli BL21(DE3)::$pgrE^{D93C}$	Used for recombinant expression of pHis8-TEV:: $pgrE^{D93C}$	This study
E. coli BL21(DE3)::$pgrE^{D93E}$	Used for recombinant expression of pHis8-TEV:: $pgrE^{D93E}$	This study
E. coli BL21(DE3)::$pgrE^{D93NL96D}$	Used for recombinant expression of pHis8-TEV:: $pgrE^{D93NL96D}$	This study

A

```
PCAU_RS14785      MTTTEQDRYLVADYDLPAMPCSVPARINPHYPTIRERHAAWSYKFLPVREPHKIPRGIAQ 60
HLB40_RS14735     MTTTEQDRYLVADYDLPAMPCSVPARINPHYPTIRERHAAWSYKFLPVREPHKIPRGIAQ 60
CXP47_RS14850     MTTTEQDRYLVADYDLPAMPCSVPARINPHYPTIRERHAAWSYKFLPVREPHKIPRGIAQ 60
                  ************************************************************

PCAU_RS14785      LDALFDSLTFPDGMPDRIYHHLCLLSTLVDGDDLAQQDPLLYEQVVLEQYDDPYSRAFAD 120
HLB40_RS14735     LDALFDSLTFPDGMPDRIYHHLCLLSTLVDGDDLAQQDPLLYEQVVLEQYDDPYSRAFAD 120
CXP47_RS14850     LDALFDSLTFPDGMPDRIYHHLCLLSTLVDGDDLAQQDPLLYEQVVLEQYDDPYSRAFAD 120
                  ************************************************************

PCAU_RS14785      AWQTIRENAPTKAVYQRIRDRWRFWLLAVQKEIEWSKAPNYDYEEMFSIRLQTVGMEPYF 180
HLB40_RS14735     AWQTIRENAPTKAVYQRIRDRWRFWLLAVQKEIEWSKAPNYDYEEMFSIRLQTVGMEPYF 180
CXP47_RS14850     AWQTIRENAPTKAVYQRIRDRWRFWLLAVQKEIEWSKAPNYDYEEMFSIRLQTVGMEPYF 180
                  ************************************************************

PCAU_RS14785      VVAEYVLDIDAGDVANDPDAIDAMTAASSFIICVNDIFSHRKECLHGDSMSTVSALRRIH 240
HLB40_RS14735     VVAEYVLDIDAGDVANDPDAIDAMTAASSYIICVNDIFSHRKECLHGDSMSTVSALRRIH 240
CXP47_RS14850     VVAEYVLDIDAGDVANDPDAIDAMTAASSYIICVNDIFSHRKECLHGDSMSTVSALRRIH 240
                  *****************************:******************************

PCAU_RS14785      GYSVQQAIDTLYTRMVDLDAKLSMLLAKLHERYAKHPLGERIHTYIDTYHAIVAGATQWH 300
HLB40_RS14735     GYSVQQAIDTLYTRMVDLDAKLSMLLAKLHERYTKHPLGERIHTYIDTYHAIVAGATQWH 300
CXP47_RS14850     GYSVQQAIDTLYTRMVDLDAKLSMLLAKLHERYTKHPLGERIHTYIDTYHAIVAGATQWH 300
                  *********************************:**************************

PCAU_RS14785      LETPRYNGVGYIWDGLRTRHITVETEMLPPHPLS-------------------------- 334
HLB40_RS14735     LETPRYNGVGYIWDGLRTRHITVETEMLPPHPLSMSAVVSKEDFTSQTNSPLQASSIASI 360
CXP47_RS14850     LETPRYNGVGYIWDGLRTRHITVETEMLPPHPLS-------------------------- 334
                  **********************************

PCAU_RS14785      -----------------    334
HLB40_RS14735     AWWWTIDNQISVALRRE    377
CXP47_RS14850     -----------------    334
```

B

**SodD**	100	19.93	20.96	19.93
**PCAU_RS14785**	19.93	100	99.40	99.40
**HLB40_RS14735**	20.96	99.40	100	100
**CXP47_RS14850**	19.93	99.40	100	100

Figure S1. Protein sequence alignments of terpene synthases from group A identified in this study.

(A) Protein sequence alignment results containing the conserved motifs highlighted in red. (B) Percent identity matrix of protein sequence alignment of terpene synthases in group A compared to SodD. Alignment was performed using Clustal Omega with default settings. Percent identity matrix was created by Clustal2.1.

A

```
QGQ83_RS27210      MSHSVQALAPQVSFYIPEMYCSVLPRIHPDYPIIDERNAAWVREFLPFTDEAALLRFLRL   60
BLQ48_RS29175      MNHSAQDFAPEVSFYIPEMYCSVLPRIHPDYPTIDELNAAWVREFLPFTDEAARLRFLRL   60
PgrE               MNHSAQDFAPEVSFYIPEMYCSVLPRIHPDYPTIDELNAAWVREFLPFTDEAARLRFLRL   60
HLB40_RS14715      MNHSAQALTPEVSFYITEMHCSVPPRINPDYPIIDELNASWVREFLPFTDEAAQLRFLRL   60
GSF17_RS15410      MNHSAQALTPEVSFYITEMHCSVPPRINPDYPIIDELNASWVREFLPFTDEAAQLRFLRL   60
CXP47_RS14830      MNHSAQALTPEVSFYITEMHCSVPPRINPDYPIIDELNASWVREFLPFTDEAAQLRFLRL   60
PCAU_RS14770       MNHSAQALTPQVSFYIPEMYCSVPPRINPDYPIIDERNASWVREFLPFTDEAAQLRFLRL   60
Pch106_6041        MNHSAQALAPEVSFYIPEMYCSVLPRIHPDYPIIDERNASWVREFLPFTDEAAQLRFLRL   60
PMI18_05943        MNHSAQSLASEVSFYIQDMHCSVLPRMHPDYPIIDELNAAWVREFLPFTDEAARLRFLRL   60
PMI30_05083        MNHSAQSLASEVSFYIPEMHCSELPRIHPDYPIIDELNAAWVREFLPFTDEAARLHFLRL   60
PMI21_00071        MNHSAQALTPEVSFYIPEMHCSVPPRIHPDYPIIDELNAAWVREFLPFTDEAARLRFLQL   60
M217_RS0102685     MNHSAQALAPEVSFYIPEMYCSVPPRIHPDYPIIDECNAAWIREFLPFADEAARLRFLRL   60
                   *.**.* :: :***** :*:**  **::**** *** **:*:******:**** *:**:*

QGQ83_RS27210      RLPMWDSLILPVGSADRLVQTSCVTSLILAIDDMPLGRHAMFHDGEVALVEGHPFAHAAQ   120
BLQ48_RS29175      RLPMWDSLIFPIGPADRIVLTSCVTSLVLAIDDMPLGRHAIFHDGEIALLDGHPFAHAAQ   120
PgrE               RLPMWDSLIFPIGPADRIVLTSCVTSLVLAIDDMPLGRHAIFHDGEIALLDGHPFAHAAQ   120
HLB40_RS14715      HTPMWDSMIFPIGSADRLVQTSCVTSLITAIDDMPLGRHAMFHDGEVALLEGHPFAHAAQ   120
GSF17_RS15410      HTPMWDSMIFPIGSADRLVQTSCVTSLITAIDDMPLGRHAMFHDGEVALLEGHPFAHAAQ   120
CXP47_RS14830      HTPMWDSMIFPIGSADRLVQTSCVTSLITAIDDMPLGRHAMFHDGEVALLEGHPFAHAAQ   120
PCAU_RS14770       HTPMWDSMIFPIGSADRLVHTSCVTSLITAIDDMPLGRHAMFHDGEVALLEGHPFARAAQ   120
Pch106_6041        HTPMWDSMIFPIGSADRLVHTSCVTSLITAIDDMPLGRHAMFHDGEVALLEGHPFARAAQ   120
PMI18_05943        HLPMWDSLIFPIGPADRLVHTSCVTSLILAIDDMPLGRHAMFHDGEVALLEGHPFAPAAQ   120
PMI30_05083        HLPMWDSLIFPIGPADRLVHTSCVTSLILAIDDMPLGRHAMFHDGEVALLEGHPFAPAAQ   120
PMI21_00071        HLPMWDCLIFPIGSADRLVHTSCVTSLILAIDDMPLGRHAMFHDGEVALLEGHPFAHAAQ   120
M217_RS0102685     HLPMWDSLLFPIGSADRLVHTSCVTSLILAIDDMPLGRHAMFDDGEIVLLEGHPFAHAAQ   120
                   : ****.:::*:* ***:* *******: ***********:*.***:.*::***** ***

QGQ83_RS27210      DIFGKLRQQMPAPVYRRYCQEWQAWFESVEEEARLVATGTVLPFDEFLELRHPNTGLFPS   180
BLQ48_RS29175      DIFGKLRQHMPAPVYRRYCREWQAWFESVEEEARLVAAGKVLPFDEFLELRHPNTGLLPY   180
PgrE               DIFGKLRQHMPAPVYRRYCREWQAWFESVEEEARLVAAGKVLPFDEFLELRHPNTGLLPY   180
HLB40_RS14715      DIFGKLRQHMPAPVYRRYCQELQAWFESVEEEARLLAAGKVLPLDEFLELRHPNTGLLPS   180
GSF17_RS15410      DIFGKLRQHMPAPVYRRYCQELQAWFESVEEEARLLAAGKVLPLDEFLELRHPNTGLLPS   180
CXP47_RS14830      DIFGKLRQHMPAPVYRRYCQELQAWFESVEEEARLLAAGKVLPLDEFLELRHPNTGLLPS   180
PCAU_RS14770       DIFGKLRQHMRAPVYRRYCQELQAWFESVEEEARLVAAGKVLPLDEFLELRHPNTGLLPS   180
Pch106_6041        DIFGKLRQHMPAPVYRRYCQELQAWFESVEEEARLVAAGKVLPLDEFLELRHPNTGLLPS   180
PMI18_05943        DIFGKLRQYMPAPVYRRYCQAWQAWFESVEEEARLVATGKVLPFDEFLELRHWNTGLLPS   180
PMI30_05083        DIFGKLRQHMPAPVYRRYCQAWQAWFESVEEEARLVATGKVLPFDEFLELRHWNTGLLPS   180
PMI21_00071        NIFGKLRQHMPAPVYRRYCQAWQAWFESVEEEARLVAAGKVLPFDEFLELRHLNTGLLPS   180
M217_RS0102685     DIFGKLRQHMPAPVYRRYCQEWQAWFESVEEEARLVAAGKVLPFDEFLDLRHLNTGLLPS   180
                   :******* * ********:  *************:*:*.***:****:*** ****:*

QGQ83_RS27210      FPVAEFLYDLDLTELLAQDQELRLAIKATNEHVGLVNDILSHRKEHTAGVTLNAMESLRM   240
BLQ48_RS29175      FPVAEFLYGLDLTELLAQDRELQLAIRVTNEHVGLVNDLLSHRKEHAIGVTLNAMESLRM   240
PgrE               FPVAEFLYGLDLTELLAQDRELQLAIRVTNEHVGLVNDLLSHRKEHAIGVTLNAMESLRM   240
HLB40_RS14715      FPVAEFLYDLDLTELLAQDRELQLAIRVTNEHVGLVNDILSHRKEHAIGVTLNAMESLRI   240
GSF17_RS15410      FPVAEFLYDLDLTELLAQDRELQLAIRVTNEHVGLVNDILSHRKEHAIGVTLNAMESLRI   240
CXP47_RS14830      FPVAEFLYDLDLTELLAQDRELQLAIRVTNEHVGLVNDILSHRKEHAIGVTLNAMESLRI   240
PCAU_RS14770       FPVAEFLYDLDLTELLAQDRELQLAIRVTNEHVGLVNDILSHRKEHAIGVTLNAMESLRI   240
Pch106_6041        FPVAEFLYDLDLTELLAQDRELQLAIRVTNEHVGLVNDILSHRKEHAIGVTLNAMESLRI   240
PMI18_05943        FPVAEFLYGLDLTELLAQDRELQLAIRVTNEHVGLVNDILSHRKEHAIGVTLNAMESLRI   240
PMI30_05083        FPVAEFLYGLDLTELLAQDRELQLAIRVTNEHVGLVNDILSHRKEHAIGVTLNAMESLRI   240
PMI21_00071        FPVAEFLYKLDLTELLAEDRELQSAIRVTNEHVGLVNDILSHRKEHTAGVTLNAMESLRM   240
M217_RS0102685     FPVAEFLYDLDLTELLEKDRELQLVIRVTNEHVGLVNDLLSHRKEHTAGVTLNAMESLRM   240
                   ******** ******* :*:**: .*:.**********:*******: ***********:
```

```
QGQ83_RS27210      AHGHNAQEAADILCQRIRDADRTRVELCEVLRRRYANRPDADRIGMYLDGLGQICAGNLR      300
BLQ48_RS29175      AHGHSPQEAADILCQRIREADRTRMELCEVLRHRYANRPDADRIGMYLDGLGRICAGNLR      300
PgrE               AHGHSPQEAADILCQRIREADRTRMELCEVLRHRYANRPDADRIGMYLDGLGRICAGNLR      300
HLB40_RS14715      AHGHSAQEAADILCQRIREADRARVELCEVLRHRYANRPDAARIGMYLDGLGRICAGNLR      300
GSF17_RS15410      AHGHSAQEAADILCQRIREADRARVELCEVLRHRYANRPDAARIGMYLDGLGRICAGNLR      300
CXP47_RS14830      AHGHSAQEAADILCQRIREADRARVELCEVLRHRYANRPDAARIGMYLDGLGRICAGNLR      300
PCAU_RS14770       VHGHSAQEAADILCQRIREADRARVELCEVLRHRYANRPDADRIGMYLDGLGRICAGNLR      300
Pch106_6041        VHGHSAQEAADILCQRIREADRARVELCEVLRHRYANRPDADRIGMYLDGLGRICAGNLR      300
PMI18_05943        AHGHSPQEAADILCQRIRKADRTRVGLCKVLRRRYANRPDADRIGMYLDGLGRICAGNLR      300
PMI30_05083        AHGHSPQEAADILCQRIRKADRTRVELCEVLRRRYADRPDADRIGMYLDGLGRICAGNLR      300
PMI21_00071        VHGHSPQEAADILCQRIREADRTRIELCEVLRRRYANRPDADRIGMYLDGLGQICAGNLR      300
M217_RS0102685     VHGHSPQEAADILCQRIREADRARVELCEVLRRRYANRPDADRIGMYLDGLGRMCAGNLR      300
                   .***. ************.***:*: **:***:***:**** **********::******

QGQ83_RS27210      WLLENDRYVDSRGKGWDWTRSRHIVLDPEPAFVRVES------------      337
BLQ48_RS29175      WLLENDRYVDSRGNGWDWTRSRLIVLDPEPSSIFGESNPGIE-------      342
PgrE               WLLENDRYVDSRGNGWDWTRSRLIVLDPEPSSIFGESNPGIE-------      342
HLB40_RS14715      WLLESDRYVDSRGNGWDWTRSRLIVLDPEPALALAEN------------      337
GSF17_RS15410      WLLESDRYVDSRGNGWDWTRSRLIVLDPEPALALAEN------------      337
CXP47_RS14830      WLLESDRYVDSRGNGWDWTRSRLIVLDPEPALALAEN------------      337
PCAU_RS14770       WLLESDRYVDSRGNGWDWTRSRLIVLDPEPASALAQS------------      337
Pch106_6041        WLLESDRYVDSRGNGWDWTRSRLIVLDPEPAPAPALAQS----------      339
PMI18_05943        WLLENDRYVDSQGHGWDWTRSRLIVLDPDPAPAHSAIAGSHYDNVKPSL      349
PMI30_05083        WLLENDRYVDSHGNGWDWTRSRLIVLDPDPAPAHSAIGGSHYDNVKPSS      349
PMI21_00071        WLLENDRYVDSRGNGWDWTRSRLIVLDPEPAPALAES------------      337
M217_RS0102685     WLLENDRYVDSRGNGWDWTRSRLIVLDPEPALALAES------------      337
                   ****.******:*:******** *****:*:
```

B

**SodD**	100	19.87	21.85	21.85	20.54	20.54	20.54	20.54	20.74	21.04	21.04	20.54	21.21
**QGQ83_RS27210**	19.87	100	86.65	86.65	86.35	86.35	86.35	86.94	87.54	86.05	86.65	88.13	86.94
**BLQ48_RS29175**	21.85	86.65	100	100	88.13	88.13	88.13	88.13	88.50	87.13	88.30	89.32	89.02
**PgrE**	21.85	86.65	100	100	88.13	88.13	88.13	88.13	88.50	87.13	88.30	89.32	89.02
**HLB40_RS14715**	20.54	86.35	88.13	88.13	100	100	100	96.14	95.55	88.13	89.02	90.50	89.32
**GSF17_RS15410**	20.54	86.35	88.13	88.13	100	100	100	96.14	95.55	88.13	89.02	90.50	89.32
**CXP47_RS14830**	20.54	86.35	88.13	88.13	100	100	100	96.14	95.55	88.13	89.02	90.50	89.32
**PCAU_RS14770**	20.54	86.94	88.13	88.13	96.14	96.14	96.14	100	97.33	87.54	88.72	90.50	89.91
**PchlO6_6041**	20.74	87.54	88.5	88.50	95.55	95.55	95.55	97.33	100	88.79	89.97	90.50	90.21
**PMI18_05943**	21.04	86.05	87.13	87.13	88.13	88.13	88.13	87.54	88.79	100	96.28	89.91	87.24
**PMI30_05083**	21.04	86.65	88.30	88.30	89.02	89.02	89.02	88.72	89.97	96.28	100	91.1	88.43
**PMI21_00071**	20.54	88.13	89.32	89.32	90.50	90.50	90.50	90.50	90.50	89.91	91.1	100	92.58
**M217_RS0102685**	21.21	86.94	89.02	89.02	89.32	89.32	89.32	89.91	90.21	87.24	88.43	92.58	100

Figure S2. Protein sequence alignments of terpene synthases from group B identified in this study.

(A) Protein sequence alignment results containing the conserved motifs highlighted in red. (B) Percent identity matrix of protein sequence alignment of terpene synthase in group B compared to SodD. Alignment was performed using Clustal Omega with default settings. Percent identity matrix was created by Clustal2.1.

A

```
PCAU_RS14790      MNDLECPIPSAIHPAADSVDTQSAVWMRRFGLCANELERNRLTHSGCGQLAARIAPRATR 60
CXP47_RS14855     MNDLECPIPSAIHPAADSVDTQSAVWMRRFGLCANEIERNRLTHSGCGQLAARIAPSATR 60
HLB40_RS14740     MNDLECPIPSAIHPAADSVDTQSAVWMRRFGLCANEIERNRLTHSGCGQLAARIAPSATR 60
                  ************************************:******************* ***

PCAU_RS14790      QTLQIVSDFFIWNVSFDDEYCDEGPLSRQPGELARTVSLIHRAIEVPESELHKGDRYACA 120
CXP47_RS14855     QTLQIVSDFFIWNVSFDDEYCDEGPLSRQPGELARTVSLIHRAIEVPESELHKGDRYACA 120
HLB40_RS14740     QTLQIVSDFFIWNVSFDDEYCDEGPLSRQPGELARTVSLIHRAIEVPESELHKGDRYACA 120
                  ************************************************************

PCAU_RS14790      MLDIRRRLDAYATPSQMYQWLMAMRGWFLAETWKAGNVAVGRIPTLDEYATLRLYSGGAM 180
CXP47_RS14855     MLDIRRRLDAYATPSQMYQWLMAMRGWFLAETWKAGNVAVGRIPTLDEYATLRLYSGGAM 180
HLB40_RS14740     MLDIRRRLDAYATPSQMYQWLMAMRGWFLAETWKAGNVAVGRIPTLDEYATLRLYSGGAM 180
                  ************************************************************

PCAU_RS14790      VFPVLTAIAENYAVSLDSLEDRRVRALTEIAASLVAWMADIASYGKEVARENGGHNLVSV 240
CXP47_RS14855     VFPVLTAIVENYAVSLDSLEDRRVKALTEIAASLVAWMADIASYGKEVARENGGHNLVSV 240
HLB40_RS14740     VFPVLTAIVENYAVSLDSLEDRRVKALTEIAASLVAWMADIASYGKEVARENGGHNLVSV 240
                  ********.***************:***********************************

PCAU_RS14790      IQHEYEGSPEWAIVQATAMCGQMMELFLRSRDSLAPDAPADLHRYLQTLGHYIRASFDWC 300
CXP47_RS14855     IQHEHGCSPEWATVQATAMCGQMMELFLRSRDSLAPDAPAELHRYLQTLGHYIRASFDWC 300
HLB40_RS14740     IQHEHGCSPEWATVQATAMCGQMMELFLRSRDSLAPDAPAELHRYLQTLGHYIRASFDWC 300
                  ****:  ***** ***************************:*******************

PCAU_RS14790      HASERYAYGDMSAVVSKEDFTSQTNSPLQASSIASIAWWWTIDNQISVALRRE        353
CXP47_RS14855     HASERYAYGDMSAVVSKEDFTSQTNSPLQASSIASIAWWWTIDNQISVALRRE        353
HLB40_RS14740     HASERYAYGDMSAVVSKEDFTSQTNSPLQASSIASIAWWWTIDNQISVALRRE        353
                  *****************************************************
```

B

**SodD**	100	31.82	31.82	31.82
**PCAU_RS14790**	31.82	100	97.45	97.45
**CXP47_RS14855**	31.82	97.45	100	100
**HLB40_RS14740**	31.82	97.45	100	100

Figure S3. Protein sequence alignments of terpene synthases from group C identified in this study.

(A) Protein sequence alignment results containing the conserved motifs highlighted in red. (B) Percent identity matrix of protein sequence alignment of terpene synthase in group C compared to SodD. Alignment was performed using Clustal Omega with default settings. Percent identity matrix was created by Clustal2.1.

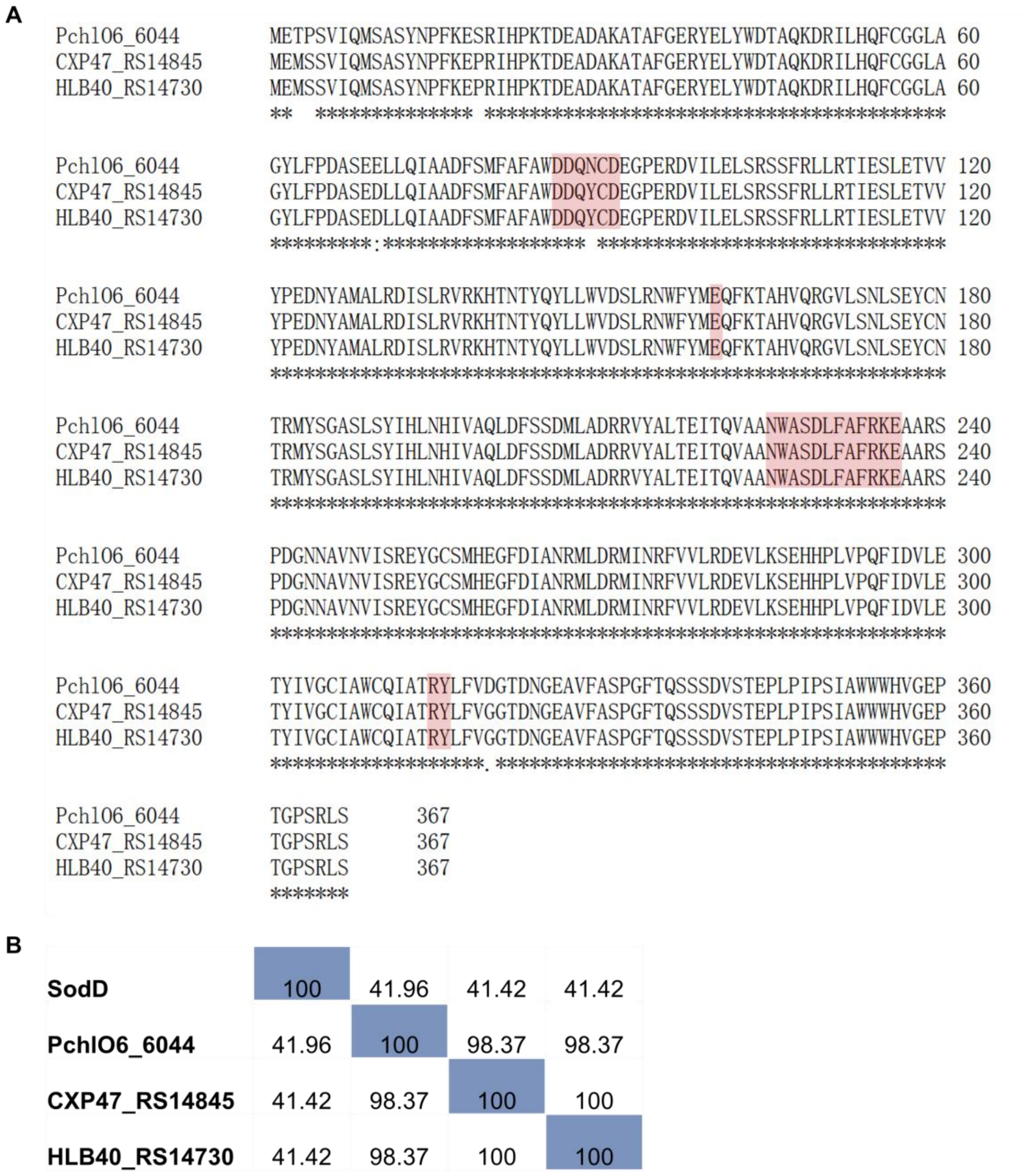

Figure S4. Protein sequence alignments of terpene synthases from group D identified in this study.

(A) Protein sequence alignment results containing the conserved motifs highlighted in red. (B) Percent identity matrix of protein sequence alignment of terpene synthase in group D compared to SodD. Alignment was performed using Clustal Omega with default settings. Percent identity matrix was created by Clustal2.1.

A

```
GSF17_RS15430        MLSPVLVVPPIPLLQKHKRHPSFDFDQPEANQFEKATRDYCDRFQLYWHEPQRLRLYATQ        60
GSF17_RS15430        MLSPVLVVPPIPLLQKHKRHPSFDFDQPEANQFEKATRDYCDRFQLYWHEPQRLRLYATQ        60
                     ************************************************************

GSF17_RS15430        NARLGALMYPKGSDELLQVGMDFCIWAFAFDDEYCDEGAISTRPDLLSRKIFDIQRAMDV        120
GSF17_RS15430        NARLGALMYPKGSDELLQVGMDFCIWAFAFDDEYCDEGAISTRPDLLSRKIFDIQRAMDV        120
                     ************************************************************

GSF17_RS15430        PEDPPCTDDRYALAARDMRIRLQKYATPLHAQRFADAFRTYMMMELHKSVDLHPTLNDYV        180
GSF17_RS15430        PEDPPCTDDRYALAARDMRIRLQKYATPLHAQRFADAFRTYMMMELHKSVDLHPTLNDYV        180
                     ************************************************************

GSF17_RS15430        LMRFYGGGGWAFAVLSHIIPLIPLPEEEYTDRRVRALAEMLTMVTVWDTEPYSYAKEQAR        240
GSF17_RS15430        LMRFYGGGGWAFAVLSHIIPLIPLPEEEYTDRRVRALAEMLTMVTVWDTEPYSYAKEQAR        240
                     ************************************************************

GSF17_RS15430        AVDDREHNLIQILMRDHQCTFAQAMERFLDLRWRVICLYQRLSAVVFKEASQAVLDYFDA        300
GSF17_RS15430        AVDDREHNLIQILMRDHQCTFAQAMERFLDLRWRVICLYQRLSAVVFKEASQAVLDYFDA        300
                     ************************************************************

GSF17_RS15430        LNLFLSGGIEWDRHNHRYRSVSGLELGQAYEGGEVAYEYPAESFEAVDLPSIAWWWRYDP        360
GSF17_RS15430        LNLFLSGGIEWDRHNHRYRSVSGLELGQAYEGGEVAYEYPAESFEAVDLPSIAWWWRYDP        360
                     ************************************************************

GSF17_RS15430        ARTG         364
GSF17_RS15430        ARTG         364
                     ****
```

B

**SodD**	100	30.64
**GSF17_RS15430**	30.64	100

Figure S5. Protein sequence alignments of terpene synthases from group E identified in this study.

(A) Protein sequence alignment results containing the conserved motifs highlighted in red. (B) Percent identity matrix of protein sequence alignment of terpene synthase in group E compared to SodD. Alignment was performed using Clustal Omega with default settings. Percent identity matrix was created by Clustal2.1.

A

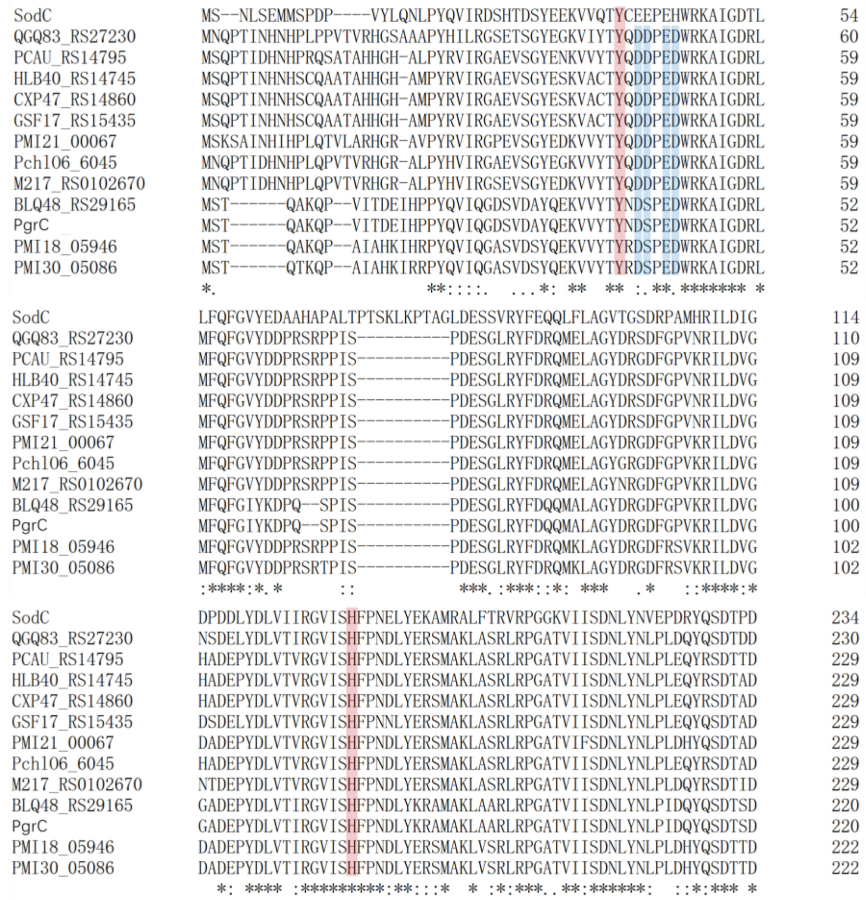

B

**SodC**	100	58.41	58.28	58.6	58.6	59.55	58.6	57.96	57.64	60.71	60.71	60.32	60
**QGQ83_RS27230**	58.41	100	87.27	87.89	87.89	88.47	87.27	88.79	90.68	80.51	80.51	81.9	81.9
**PCAU_RS14795**	58.28	87.27	100	95.65	95.65	92.52	90.68	93.77	93.17	83.33	83.33	87.9	87.9
**HLB40_RS14745**	58.6	87.89	95.65	100	100	96.88	89.44	90.97	90.68	81.73	81.73	85.35	85.35
**CXP47_RS14860**	58.6	87.89	95.65	100	100	96.88	89.44	90.97	90.68	81.73	81.73	85.35	85.35
**GSF17_RS15435**	59.55	88.47	92.52	96.88	96.88	100	89.41	89.1	89.41	80.71	80.71	84.66	84.35
**PMI21_00067**	58.6	87.27	90.68	89.44	89.44	89.41	100	89.72	91.93	83.01	83.01	87.26	86.94
**PchlO6_6045**	57.96	88.79	93.77	90.97	90.97	89.1	89.72	100	94.08	81.99	81.99	84.98	84.98
**M217_RS0102670**	57.64	90.68	93.17	90.68	90.68	89.41	91.93	94.08	100	82.69	82.69	86.31	86.31
**BLQ48_RS29165**	60.71	80.51	83.33	81.73	81.73	80.71	83.01	81.99	82.69	100	100	88.18	86.58
**PgrC**	60.71	80.51	83.33	81.73	81.73	80.71	83.01	81.99	82.69	100	100	88.18	86.58
**PMI18_05946**	60.32	81.9	87.9	85.35	85.35	84.66	87.26	84.98	86.31	88.18	88.18	100	98.1
**PMI30_05086**	60	81.9	87.9	85.35	85.35	84.35	86.94	84.98	86.31	86.58	86.58	98.1	100

Figure S6. Protein sequence alignments of methyltransferases from group I identified in this study.

(A) Protein sequence alignment results of methyltransferases from group I with SodC (partial alignment result shown here). The conserved catalytic dyad in SodC-type methyltransferases highlighted in red and the conserved acidic amino acid rich region are highlighted in blue. (B) Percent identity matrix of protein sequence alignment of methyltransferases in group I compared to SodC. Alignment was performed using Clustal Omega with default settings. Percent identity matrix was created by Clustal2.1.

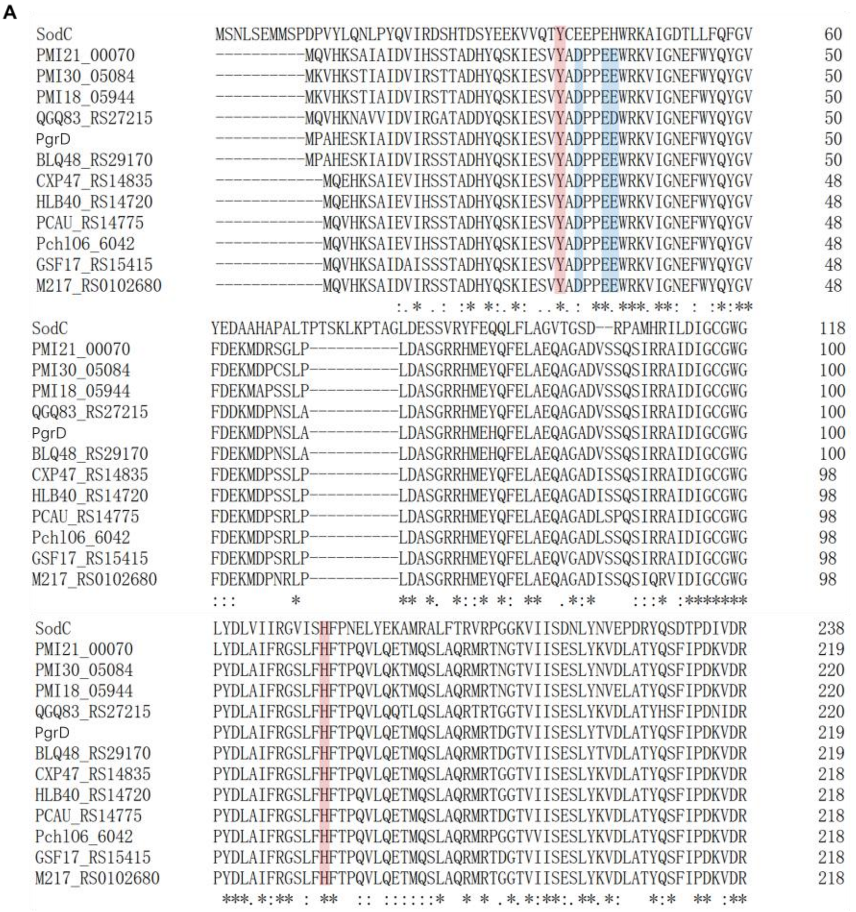

B

**SodC**	100	44.41	45.25	45.57	44.26	44.74	44.74	44.55	44.55	43.89	43.89	43.89	44.22
**PMI21_00070**	44.41	100	91.83	90.52	86.27	89.87	89.87	90.46	90.46	90.13	90.13	90.79	90.13
**PMI30_05084**	45.25	91.83	100	97.11	85.67	90.85	90.85	89.18	89.18	89.18	88.52	90.16	89.51
**PMI18_05944**	45.57	90.52	97.11	100	84.36	89.54	89.54	88.2	88.2	88.2	87.54	89.18	88.2
**QGQ83_RS27215**	44.26	86.27	85.67	84.36	100	87.91	87.91	88.52	88.52	88.2	87.87	88.52	89.84
**PgrD**	44.74	89.87	90.85	89.54	87.91	100	100	91.45	91.45	91.45	90.46	92.43	91.78
**BLQ48_RS29170**	44.74	89.87	90.85	89.54	87.91	100	100	91.45	91.45	91.45	90.46	92.43	91.78
**CXP47_RS14835**	44.55	90.46	89.18	88.2	88.52	91.45	91.45	100	100	95.74	96.72	96.07	96.39
**HLB40_RS14720**	44.55	90.46	89.18	88.2	88.52	91.45	91.45	100	100	95.74	96.72	96.07	96.39
**PCAU_RS14775**	43.89	90.13	89.18	88.2	88.2	91.45	91.45	95.74	95.74	100	97.7	96.39	96.72
**PchIO6_6042**	43.89	90.13	88.52	87.54	87.87	90.46	90.46	96.72	96.72	97.7	100	95.74	96.39
**GSF17_RS15415**	43.89	90.79	90.16	89.18	88.52	92.43	92.43	96.07	96.07	96.39	95.74	100	96.72
**M217_RS0102680**	44.22	90.13	89.51	88.2	89.84	91.78	91.78	96.39	96.39	96.72	96.39	96.72	100

Figure S7. Protein sequence alignments of methyltransferases from group II identified in this study.

(A) Protein sequence alignment results of methyltransferases from group II with SodC (partial alignment result shown here). The conserved catalytic dyad in SodC-type methyltransferases highlighted in red and the conserved acidic amino acid rich region are highlighted in blue. (B) Percent identity matrix of protein sequence alignment of methyltransferases in group II compared to SodC. Alignment was performed using Clustal Omega with default settings. Percent identity matrix was created by Clustal2.1.

A

```
BLQ48_RS29180       MSVTAQPGRPAPDHHAQFIELLSTSLAQNAFIKLVLAKYVGDEAELQRLIIKAVTVKEQP 60
FIV39_RS03770       MSVTAQPGRPAPDHHAQFIELLSTSLAQNAFIKLVLAKYVGDEAELQRLIIKAVTVKEQP 60
SodC                ------------------------------------------------------------ 0

BLQ48_RS29180       CLSFVYRYKTRDITKNLALADGVAAIAELL---PASFKNAHLLSLTDEAQLEYSKKNKSS 117
FIV39_RS03770       CLSFVYRYKTRDITKNLALADGVAAIAELL---PASFKNAHLLSLTDEAQLEYSKKNKSS 117
SodC                ----------------------MSNLSEMMSPDPVYLQNLPYQVIRDSHTDSYEEKV-VQ 37
                                          :: ::*::   *. ::*     : *.    .*.:*   .

BLQ48_RS29180       LFRSKPQQLREAPSAEHNREKNRFLDLSRPFLADLGVTDAR---QALIP------SMSRK 168
FIV39_RS03770       LFRSKPQQLREAPSAEHNREKNRFLDLSRPFLADLGVTDAR---QALIP------SMSRK 168
SodC                TYCEEPEHWRKA--------------IGDTLLFQFGVYEDAAHAPALTPTSKLKPTAGLD 83
                     : .:*:: *:*              :.   :* ::** :      ** *      : . .

BLQ48_RS29180       WKQINKFI-EVFSHALTSSPLKLDQPVRVADFGSGKGYLTFAIHDYLRNTLKAEGEVTGV 227
FIV39_RS03770       WKQINKFI-EVFSHALTSSPLKLDQPVRVADFGSGKGYLTFAIHDYLRNTLKAEGEVTGV 227
SodC                ESSVRYFEQQLFLAGVTGS--DRPAMHRILDIGCGWGYILKHLAERYPEC----QRLDGV 137
                     ..:. *  ::*  .:*.*  .      *: *:*.* **:   : :   :      .: **

BLQ48_RS29180       ELREDMVTLCNTAAARLEHPG--LVFKCGDVRS-VAP--SELDVMIALHA------CD-- 274
FIV39_RS03770       ELREDMVTLCNTAAARLEHPG--LVFKCGDVRS-VAP--SELDVMIALHA------CD-- 274
SodC                NVSAQQLNYCARSHAEQGLSGRINLFLCNAQDIGLLPDPDDLYDLVIIRGVISHFPNELY 197
                    ::  : :. *  : *.     *   :* *.      : *  .:*  :: ::.       :

BLQ48_RS29180       IATDYAIHTGIRSGASIIMCSPCCH-KQIRLQIQSPALLKPMLQYGLHLGQQAEMVTDSL 333
FIV39_RS03770       IATDYAIHTGIRSGASIIMCSPCCH-KQIRLQIQSPALLKPMLQYGLHLGQQAEMVTDSL 333
SodC                EKAMRALFTRVRPGGKVIISDNLYNVEPDRYQSDTPDIVDRLACR-----H---QKTPAY 249
                      :  *:.* :* *..:*:..    : :  * * ::* ::. :         :     * :

BLQ48_RS29180       RALFLEACGYETKVFEFISLEHTNKNKMI---LAVKRAEPVDNAQLLEKIQALKAFYHIS 390
FIV39_RS03770       RALFLEACGYETKVFEFISLEHTNKNKMI---LAVKRAEPVDNAQLLEKIQALKAFYHIS 390
SodC                FSQVLEDSGFTIKDFRVLPSNVDVAHWLMDSKANIERHFPTGVDGALEELRVLAENWSV- 308
                      : .** .*:  * *..:  :     : ::      ::*  *..    **:::.*   : :

BLQ48_RS29180       EHCLETLLRA--DGYLA-----       405
FIV39_RS03770       EHCLETLLRA--DGYLA-----       405
SodC                -----ALLKNKVSTYSVIACKK       325
                         :**:   . * .
```

B

**SodC**	100	19.44	19.44
**BLQ48_RS29180**	19.44	100	100
**PgrF**	19.44	100	100

Figure S8. Protein sequence alignments of methyltransferases from group III identified in this study.

(A) Protein sequence alignment results of methyltransferases from group III with SodC. (B) Percent identity matrix of protein sequence alignment of methyltransferases in group III compared to SodC. Alignment was performed using Clustal Omega with default settings. Percent identity matrix was created by Clustal2.1.

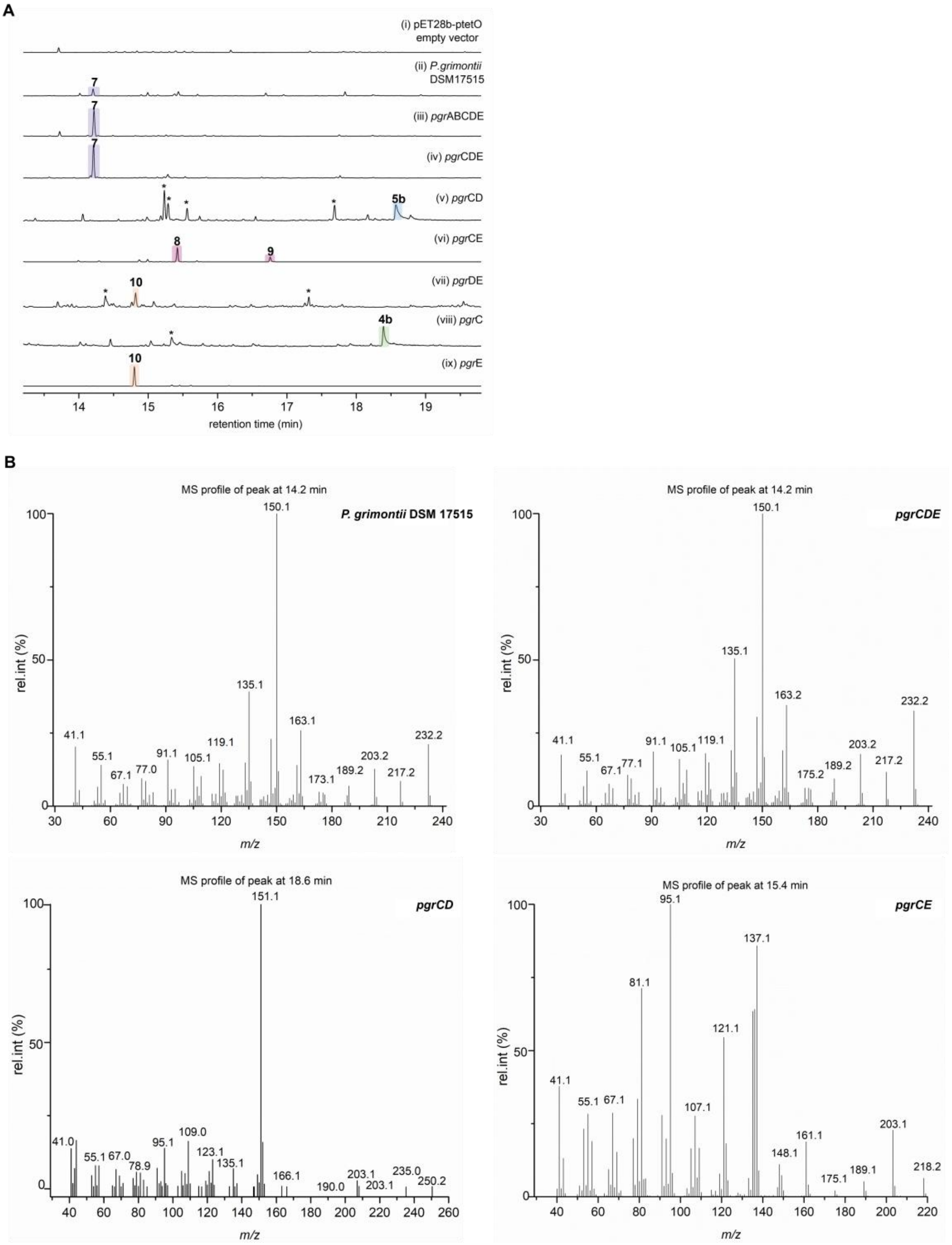
A
(i) pET28b-ptetO empty vector
(ii) P.grimontii DSM17515
(iii) pgrABCDE
(iv) pgrCDE
(v) pgrCD
(vi) pgrCE
(vii) pgrDE
(viii) pgrC
(ix) pgrE
7
5b
8
9
10
4b
retention time (min)
B
MS profile of peak at 14.2 min
P. grimontii DSM 17515
pgrCDE
MS profile of peak at 18.6 min
pgrCD
MS profile of peak at 15.4 min
pgrCE
rel.int (%)
m/z

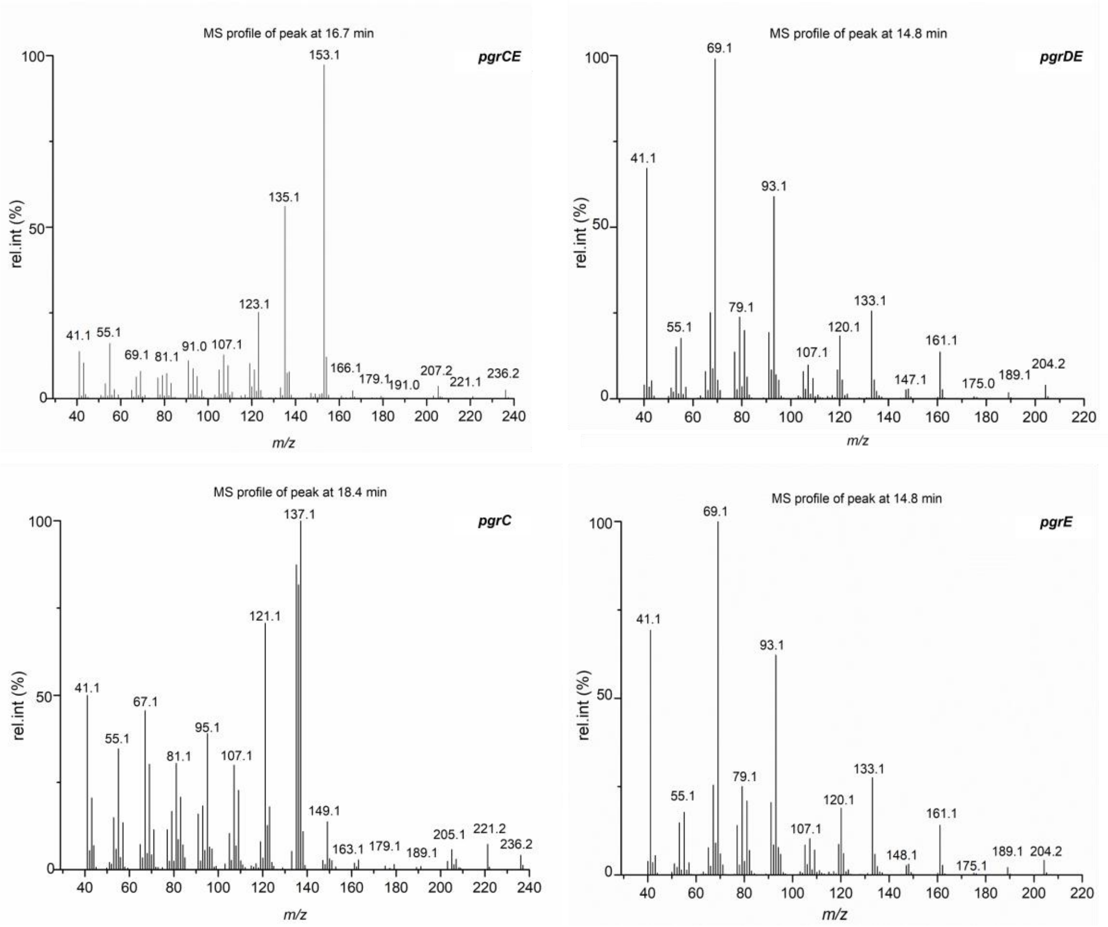

Figure S9. GC-EI-MS analyses of extracts of *P. grimontii* DSM 17515 and *in vivo* heterologous expression of recombinant constructs in *E. coli.*

(A) Comparison of GC-EI-MS total ion chromatograms of volatile compounds produced by *P. grimontii* DSM 17515 and the *E. coli* BAP1 strains carrying vector pET28b-ptetO-GFPv2 containing empty vector, *pgr*ABCDE, *pgr*CDE, *pgr*CD, *pgr*CE, *pgr*DE, *pgr*C and *pgr*E. Peaks labelled with an asterisk indicate uncharacterized VOCs. The signal intensity of **7** in (iii) was set to 100%, to which all other chromatograms were normalized. (B) GC-EI-MS spectra of elucidated VOCs produced by *P. grimontii* DSM 17515 and by recombinant *E. coli* BAP1 strains. All samples were extracted with pentane and then measured by GC-EI-MS.

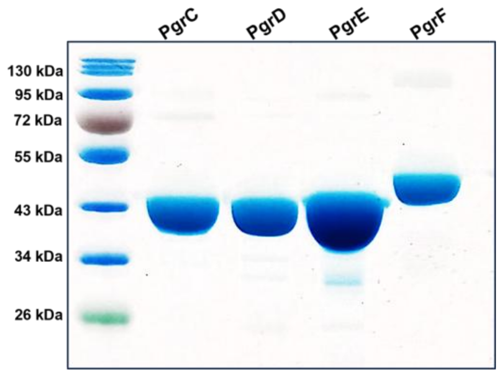

Figure S10. SDS-PAGE analyses of the purified methyltransferases PgrC, PgrD and PgrF and terpene synthases PgrE heterologously expressed as recombinant proteins in *E.coli*.

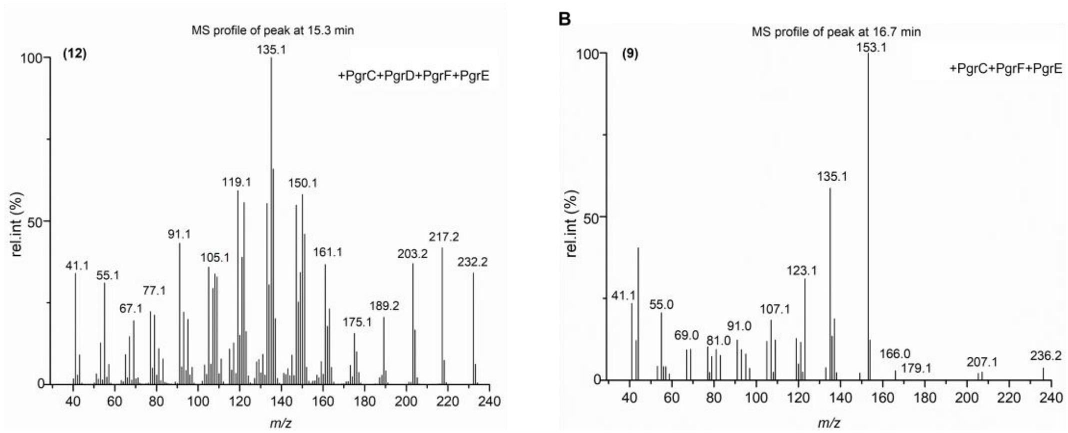

Figure S11. GC-EI-MS analyses of extracts of *in vitro* enzymatic assays using combinations of methyltransferases PgrC, PgrD, PgrF and terpene synthase PgrE from *Pseudomonas grimontii* DSM 17515.

(A) GC-EI-MS fingerprint of compound **12** at retention time of 15.3 min in the *in vitro* enzyme assay extracts in the presence of FPP, SAM, and PgrC, PgrD, PgrF, and PgrE. (B) GC-EI-MS fingerprint of compound **9** at retention time of 16.7 min in the extracts of *in vitro* enzyme assay in the presence of FPP, SAM, and PgrC, PgrF, and PgrE.

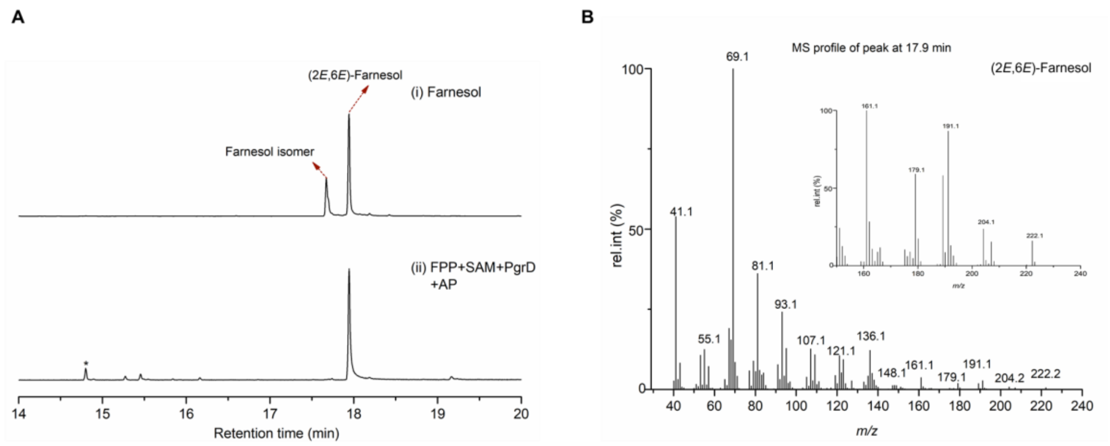

Figure S12. GC-EI-MS analyses of extracts of *in vitro* enzymatic assays using combinations of methyltransferases PgrD from *Pseudomonas grimontii* DSM 17515.

(A) GC-EI-MS chromatograms of comerical farnesol and the result of the enzyme assay of FPP with SAM and PgrD. (B) GC-MS fingerprint of farnesol at retention time 17.9 min. An asterisk represents a peak whose structure has not been characterized.

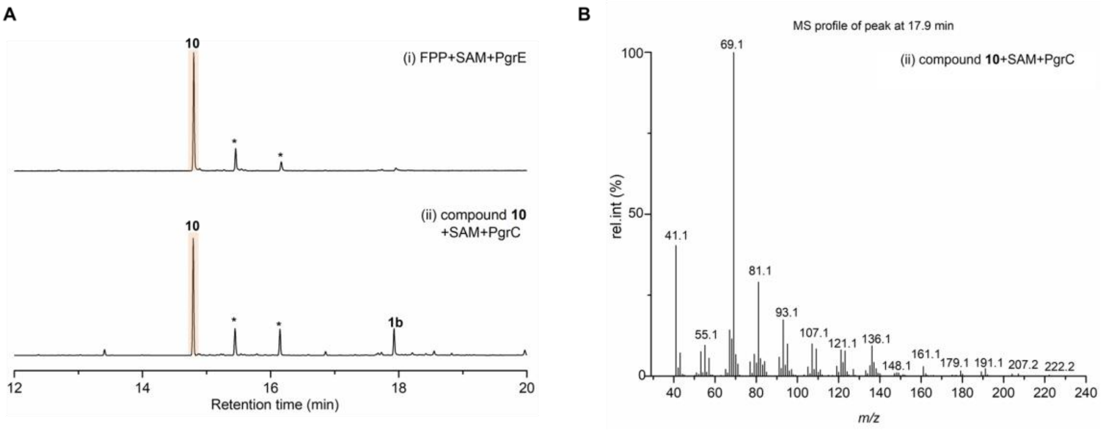

Figure S13. GC-EI-MS analyses of extracts of *in vitro* stepwise enzymatic assays using terpene synthases PgrE and methyltransferases PgrC from *Pseudomonas grimontii* DSM 17515.
(A) GC-EI-MS chromatograms of *in vitro* assay (i) in the presence of FPP, SAM and PgrE, and assay (ii) with the product obtained in (i) and SAM and PgrC. β-farnesene (**10**) is highlighted in yellow. (B) GC-MS fingerprint of extracts of *in vitro* assay (ii) at rentation time 17.9 min, which is consistent with compound **1b.** An asterisk represents a peak whose structure has not been characterized.

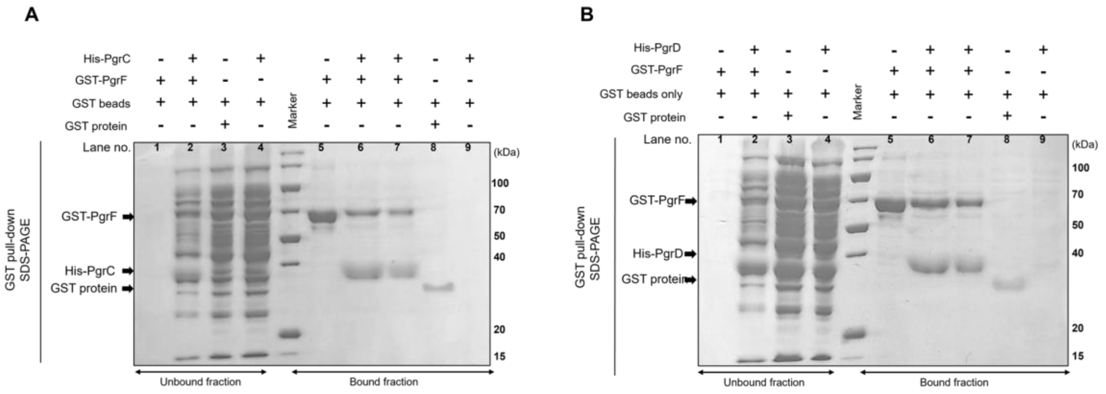

Figure S14. Analyses of the interaction of PgrC (His tag) or PgrD (His tag) with PgrF (GST tag) using GST pull down assays.

Lanes 1-4 are unbound fractions, and the corresponding bound fractions are shown in lanes 5-9, where the loading amount in lane 6 is 1.5 times that of lane 7. As a control, cell lysate of His-tagged (**A**) PgrC (36 kDa)/(**B**) PgrD (35 kDa) was incubated with GST beads alone, and the bound and unbound fractions are shown in lanes 9 and 4. His-PgrC/His-PgrD and GST non-fusion protein (28 kDa) were used as controls, and the bound and unbound fractions are shown in lanes 8 and 3. Lanes 5 and 1 show the elution and flow-through fractions, respectively, after GST-tagged PgrF (71 kDa) was incubated with glutathione-agarose beads alone as a control. The bound fractions after incubation of His-tagged (**A**) PgrC/(**B**) PgrD cell lysates with GST-tagged protein (GST-PgrF) are shown in lanes 6 and 7, and the unbound fractions in lane 2. The legend at the top of A and B indicate various combinations of His-tagged PgrC/PgrD, GST-tagged PgrF protein, GST beads alone, and GST protein. (+) indicates the presence of a component in each lane, while (-) indicates the absence of a component in each lane. Unless otherwise specified, all samples of the unbound fraction have the same loading amount, while the loading amount of the bound fraction is the same.

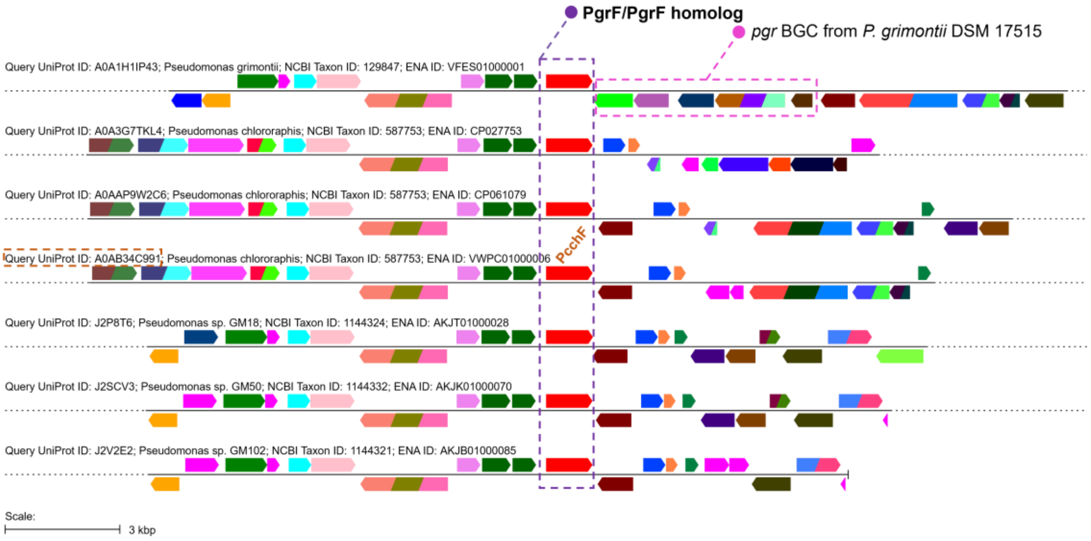

Figure S15. Genomic neighborhood analysis of PgrF homologs in selected *Pseudomonas* sp. The figure depicts the genomic neighborhood analysis corresponding to the PgrF homologs listed in Table S5, after removal of identical entries from different bacterial strains. The homolog C4K27_RS06760 (PcchF) from *Pseudomonas chlororaphis* subsp. *chlororaphis* DSM 50083, functionally characterized in this work, is included. Protein identifiers correspond to the following UniProt entries: A0A1H1IP43 (WP_090408245.1), A0A3G7TKL4 (WP_009047435.1), A0AAP9W2C6 (WP_041988785.1), A0AB34C991 (WP_053259895.1), J2P8T6 (WP_007935760.1), J2SCV3 (WP_008010838.1), and J2V2E2 (WP_007903762.1)

```
WP_025806786.1     MSATATPASLAPDHHAQFLELLDTSLAQNAFIKLVLAKHVGAEADLQRIIIKQVTVKEQP 60
PcchF              MSATATPASLAPDHHAQFLELLETSLAQNAFIKLVLAKHVGAEADLQRLIIKQLTVKEQP 60
WP_009047435.1     MSATATPASLAPDHHAQFLELLETSLAQNAFIKLVLAKHVGAEADLQRLIIKQLTVKEQP 60
WP_081362479.1     MSATATPASLAPDHHAQFLELLETSLAQNAFIKLVLAKHVGAEADLQRLIIKQLTVKEQP 60
WP_016702088.1     MSATATPASLAPDHHAQFLELLETSLAQNAFIKLVLAKHVGAEADLQRLIIKQLTVKEQP 60
WP_041988785.1     MSATATPASLAPDHHAQFLELLETSLAQNAFIKLVLAKHVGAEADLQRLIIKQLTVKEQP 60
WP_007935760.1     MSVTATPASLAPDHHAQFIDLLQTSLDANAFIKLVLAKYVGGEADLQRLIIKQLTVKDQS 60
WP_007903762.1     MSVTATPASLAPDHHAQFIDLLQASLDQNAFIKLVLAKYVGTEADLQRLIIKQLTVKDQQ 60
WP_008010838.1     MSVTATPASLVPDHHAQFIDLLQASLDQNAFIKLVLAKYVGGEADLQRLIIKQLTVKDQP 60
PgrF               MSVTAQPGRPAPDHHAQFIELLSTSLAQNAFIKLVLAKYVGDEAELQRLIIKAVTVKEQP 60
WP_281112651.1     MSVTAQPASPAPDHHAQFIELLSTSLAQNAFIKLVLAKYVGDEAELQRLIIKAVTVKEQP 60
                   **.** *.  .*******::**.:**  **********:** **:***:***  :***:*
WP_025806786.1     CLSFVYRYKTRDITKNFPLGEGVALIAGLLPASFKNAHLLSLTDEVQLEYSKKGKSSLFK 120
PcchF              CLSFVYRYKTRDITKNFPLDEGVALIAGLLPASFKNAHLLSLTDEVQLEYSKKGKSSLFK 120
WP_009047435.1     CLSFVYRYKTRDITKNFPLDEGVALIAGLLPASFKNAHLLSLTDEVQLEYSKKGKSSLFK 120
WP_081362479.1     CLSFVYRYKTRDITKNFPLDEGVALIAGLLPASFKNAHLLSLTDEVQLEYSKKGKSSLFK 120
WP_016702088.1     CLSFVYRYKTRDITKNFPLGEGVALIAGLLPASFKNAHLLSLTDEVQLEYSKKGKSSLFK 120
WP_041988785.1     CLSFVYRYKTRDITKNFPLGEGVALIAGLLPASFKNAHLLSLTDEVQLEYSKKGKSSLFK 120
WP_007935760.1     CLSFVYRYKTRDITKNLPIAEGVATIAALLPASFKNAHLLSLTDEAQLEYSKKGKSSLFK 120
WP_007903762.1     CLSFVYRYKTRDITKNLPITEGVATIAALLPASFKNAHLLSLTDEAQLEYSKKGKSSLFK 120
WP_008010838.1     CLSFVYRYKTRDITKNLPLAEGVATIAALLPASFKNAHLLSLTDEAQLEYSKKGKSSLFK 120
PgrF               CLSFVYRYKTRDITKNLALADGVAAIAELLPASFKNAHLLSLTDEAQLEYSKKNKSSLFR 120
WP_281112651.1     CLSFVYRYKTRDITKNLALADGVAAIAALLPGSFKNAHLLSLTDEAQLEYSKKNKSSLFR 120
                   ****************: : :*** ** ***.**************.*******.*****:
WP_025806786.1     GRAQQQREVPSAEHNREKNRYLELSRPFLTDLGVTNKQHELIPAMSRKWKQINKFIEVFS 180
PcchF              GKAQQQREVPSAEHNREKNRYLELSRPFLTDLGVTNKQHELIPAMSRKWKQINKFIEVFS 180
WP_009047435.1     GKAQQQREVPSAEHNREKNRYLELSRPFLTDLGVTNKQHELIPAMSRKWKQINKFIEVFS 180
WP_081362479.1     GKAQQQREVPSAEHNREKNRYLELSRPFLTDLGVTNKQHELIPAMSRKWKQINKFIEVFS 180
WP_016702088.1     GKAQQQREVPSAEHNREKNRYLELSRPFLTDLGVTNKQHELIPAMSRKWKQINKFIEVFS 180
WP_041988785.1     GKAQQQREVPSAEHNREKNRYLELSRPFLTDLGVTNKQHELIPAMSRKWKQINKFIEVFS 180
WP_007935760.1     SKPQQLREVPSAEHNREKNRFLDLSRPFLKDLGVTNAQHELIPAMSRKWKQINKFIEVFS 180
WP_007903762.1     SKPQQLREVPSAEHNREKNRFLDLSRPFLKDLGVTNAQHELIPAMSRKWKQINKFIEVFS 180
WP_008010838.1     SKPQQLREVPSAEHNREKNRFLDLSRPFLKDLGVTNAQHELIPAMSRKWKQINKFIEVFS 180
PgrF               SKPQQLREAPSAEHNREKNRFLDLSRPFLADLGVTDARQALIPSMSRKWKQINKFIEVFS 180
WP_281112651.1     SKPQQLREAPSVEHNREKHRFLDLSRPFLADLGVTDARQALIPSMSRKWKQINKFIEVFS 180
                   .: ** **.**.******:*:*:****** *****: :: ***:****************
WP_025806786.1     HALSASPIDLQQPVRVADFGSGKGYLTFAIHDYLCNTLQAQGQVTGVELREDMVTLCNAA 240
PcchF              HALSSSPIDLQQPVRVADFGSGKGYLTFAIHDYLCNTLQAQGQVTGVELREDMVTLCNAA 240
WP_009047435.1     HALSSSPIDLQQPVRVADFGSGKGYLTFAIHDYLCNTLQAQGQVTGVELREDMVTLCNAA 240
WP_081362479.1     HALSSSPIDLQQPVRVADFGSGKGYLTFAIHDYLCNTLQAQGQVTGVELREDMVTLCNAA 240
WP_016702088.1     HALSSSPIDLQQPVRVADFGSGKGYLTFAIHDYLCNTLQAQGQVTGVELREDMVTLCNAA 240
WP_041988785.1     HALSSSPIDLQQLVRVADFGSGKGYLTFAIHDYLCNTLQAQGQVTGVELREDMVTLCNAA 240
WP_007935760.1     HALTSSPLALDKPVRVADFGSGKGYLTFAIHDYLRNTLKAEGEVTGVELREDMVTLCNSA 240
WP_007903762.1     HALTSSPLALDKPVRVADFGSGKGYLTFAIHDYLRNTLKAEGEVTGVELREDMVNLCNSA 240
WP_008010838.1     HALTSSPLALDKPVRVADFGSGKGYLTFAIHDYLRNTLKAEGEVTGVELREDMVNLCNSA 240
PgrF               HALTSSPLKLDQPVRVADFGSGKGYLTFAIHDYLRNTLKAEGEVTGVELREDMVTLCNTA 240
WP_281112651.1     HALTSSPLKLDQPVRVADFGSGKGYLTFAIHDYLRNTLKAEGEVTGVELREDMVTLCNTA 240
                   ***::**: *:: ********************* ***:*:*:***********.***:*
```

```
WP_025806786.1      AARLDHQGLEFKCGDVRSVAPSELDVMIALHACDIATDYAIHTGIRSGASIIMCSPCCHK 300
PcchF               AARLDHQGLEFKCGDVRSVAPSELDVMIALHACDIATDYAIHTGIRSGAAIIMCSPCCHK 300
WP_009047435.1      AARLEHQGLEFKCGDVRSVAPSELDVMIALHACDIATDYAIHTGIRSGASIIMCSPCCHK 300
WP_081362479.1      AARLEHQGLEFKCGDVRSVAPSELDVMIALHACDIATDYAIHTGIRSGASIIMCSPCCHK 300
WP_016702088.1      AARLDHQGLEFKCGDVRSVAPSELDVMIALHACDIATDYAIHTGIRSGASIIMCSPCCHK 300
WP_041988785.1      AARLDHQGLEFKCGDVRSVAPSELDVMIALHACDIATDYAIHTGIRSGASIIMCSPCCHK 300
WP_007935760.1      AAKLEHPGLVFKCGDVRSVAPSELDVMIALHACDIATDYAIHTGIRSGAAIIMCSPCCHK 300
WP_007903762.1      AAKLEHPGLVFKCGDVRSVAPSELDVMIALHACDIATDYAIHTGIRSGASIIMCSPCCHK 300
WP_008010838.1      AAKLEHPGLVFKCGDVRSVAPSELDVMIALHACDIATDYAIHTGIRSGASIIMCSPCCHK 300
PgrF                AARLEHPGLVFKCGDVRSVAPSELDVMIALHACDIATDYAIHTGIRSGASIIMCSPCCHK 300
WP_281112651.1      AARLEHPGLVFKCGDVRSVAPSQLDVMIALHACDIATDYAIHTGIRSGASIIMCSPCCHK 300
                    **:*:* ** ************:**************************:**********

WP_025806786.1      QIRPQMQSPALLKPMLQYGLHLGQQAEMVTDSLRALFLEACGYETKVFEFISLEHTNKNK 360
PcchF               QIRPQMQSPALLKPMLQYGLHLGQQAEMVTDSLRALFLEACGYETKVFEFISLEHTNKNK 360
WP_009047435.1      QIRPQMQSPALLKPMLQYGLHLGQQAEMVTDSLRALFLEACGYETKVFEFISLEHTNKNK 360
WP_081362479.1      QIRPQMQSPALLKPMLQYGLHLGQQAEMVTDSLRALFLEACGYETKVFEFISLEHTNKNK 360
WP_016702088.1      QIRPQMQSPALLKPMLQYGLHLGQQAEMVTDSLRALFLEACGYETKVFEFISLEHTNKNK 360
WP_041988785.1      QIRPQMQSPALLKPMLQYGLHLGQQAEMVTDSLRALFLEACGYETKVFEFISLEHTNKNK 360
WP_007935760.1      QIRLQIQSPALLKPMLQYGLHLGQQAEMVTDSLRALFLEACGYETKVFEFISLDHTNKNK 360
WP_007903762.1      QIRLQIQSPALLKPMLQYGLHLGQQAEMVTDSLRALFLEACGYETKVFEFISLDHTNKNK 360
WP_008010838.1      QIRLQIQSPALLKPMLQYGLHLGQQAEMVTDSLRALFLEACGYETKVFEFISLDHTNKNK 360
PgrF                QIRLQIQSPALLKPMLQYGLHLGQQAEMVTDSLRALFLEACGYETKVFEFISLEHTNKNK 360
WP_281112651.1      QIRLQIQSPALLKPMLQYGLHLGQQAEMVTDSLRALFLEACGYETKVFEFISLEHTNKNK 360
                    *** *:***********************************************:******

WP_025806786.1      MILAVKRAEPQDPTQLLAKIGELKAFYHISEHCLETLLRADGYLS---    405
PcchF               MILAVKRAEPQDPAQLLAKIGELKAFYHISEHCLETLLRADGYLG---    405
WP_009047435.1      MILAVKRAEPQDPTQLLAKIGELKAFYHISEHCLETLLRADGYLG---    405
WP_081362479.1      MILAVKRAEPQDPTQLLAKIGELKAFYHISEHCLETLLRADGYLS---    405
WP_016702088.1      MILAVKRAEPQDPTQLLAKIGELKAFYHISEHCLETLLRADGYLS---    405
WP_041988785.1      MILAVKRAEPQDPTQLLAKIGELKAFYHISEHCLETLLRADGYLG---    405
WP_007935760.1      MILAVKRAEPVDPAQLLVKIQELKDFYHISEHCLETLLRADGFLVAKA    408
WP_007903762.1      MILAVKRAEPVDPAQLLVKIQELKDFYHISEHCLETLLRADGYL----    404
WP_008010838.1      MILAVKRAEPVDPAPLLVKIQELKDFYHISEHCLETLLRADGYL----    404
PgrF                MILAVKRAEPVDNAQLLEKIQALKAFYHISEHCLETLLRADGYLA---    405
WP_281112651.1      MILAVKRAEPVDNAQLLERIQELKAFYHITEHCLETLLRADGYLK---    405
                    ********** * : ** :*  ** ****:************:*
```

Figure S16. Conserved sequence features of PgrF-type group III methyltransferases analyzed in this study.

Protein sequence alignment of PgrF, PcchF and selected homologs from *Pseudomonas* sp. (detailed in Table S5) was performed using CLUSTAL X (1.83). The alignment highlights the high sequence conservation within this enzyme class.

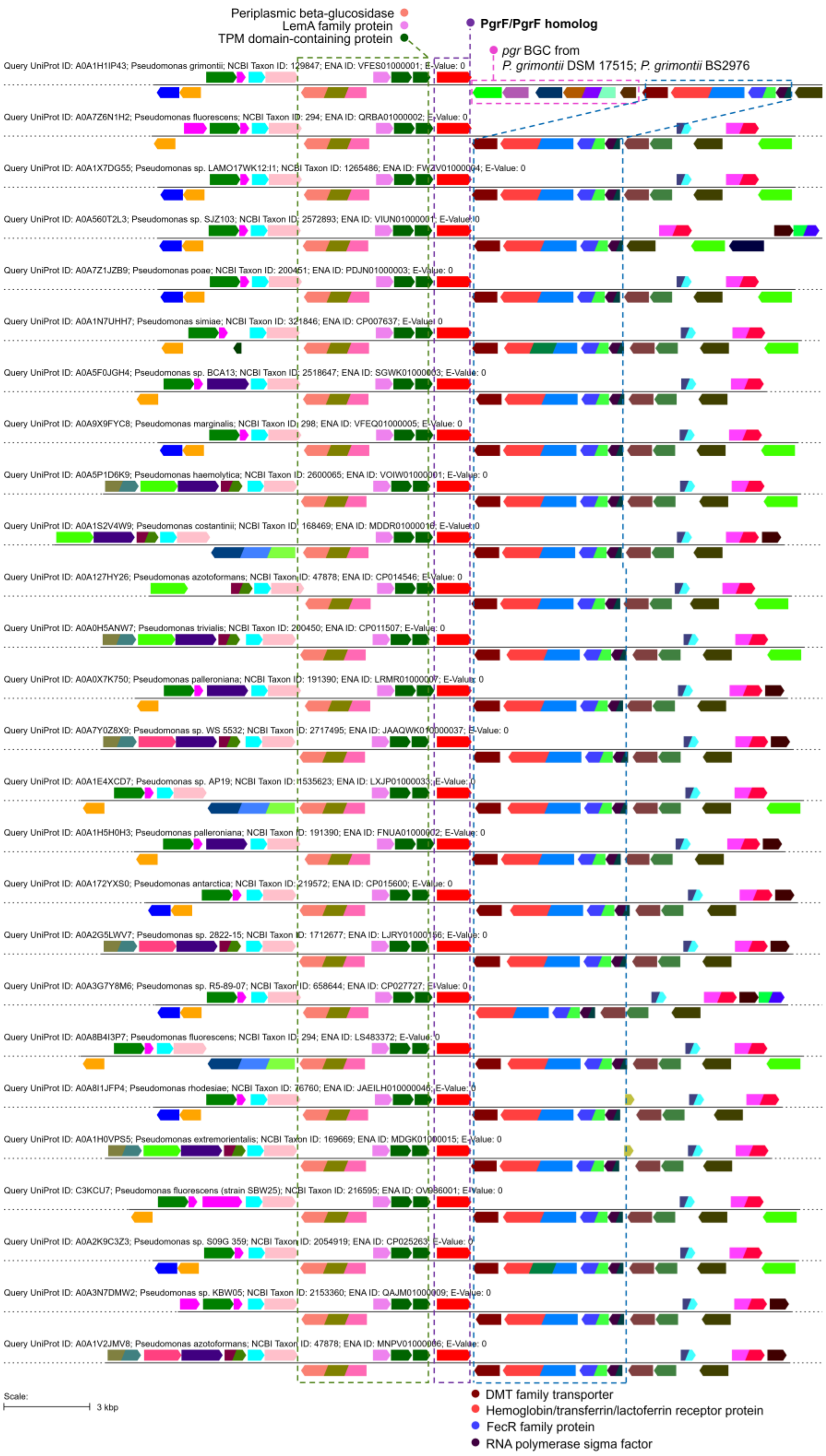

Figure S17. Genomic neighborhood analysis of PgrF-like group III methyltransferases. Global genomic neighborhood analysis was performed using EFI GNT with *pgrF* as probe. The analysis reveals that *pgrF* homologs are widely distributed in *Pseudomonas* sp. and that *pgrF* homologs typically reside in conserved loci associated with genes unrelated to terpene biosynthesis. In *P. grimontii*, by contrast, the *pgrABCDE* grimophan BGC is present within this conserved region (top entry), suggesting acquisition and insertion of this BGC into this locus by horizontal gene transfer.

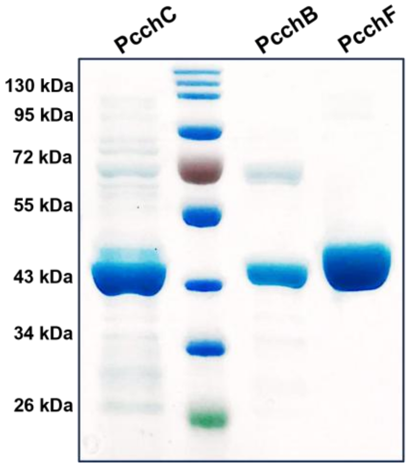

Figure S18. SDS-PAGE analyses of the purified proteins PcchB, PcchC and PcchF from *P. chlororaphis* subsp. *chlororaphis* DSM 50083.

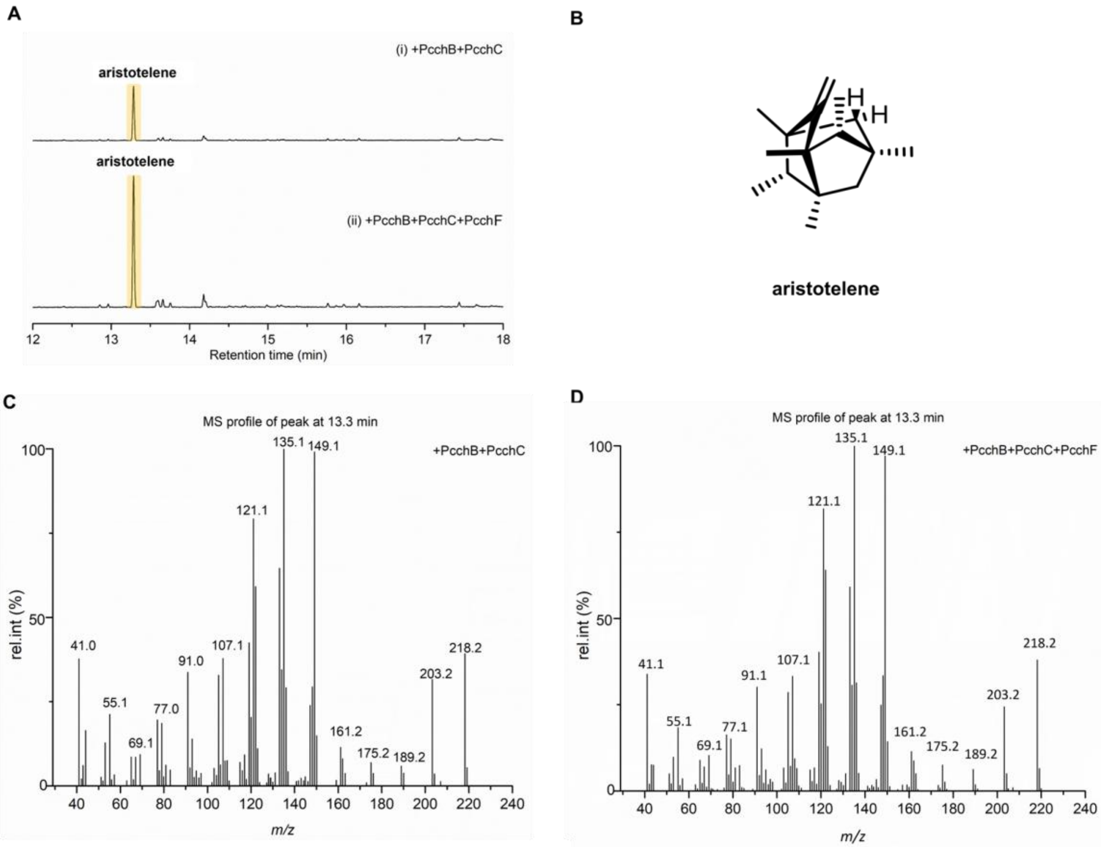

Figure S19. GC-EI-MS analyses of VOCs obtained by *in vitro* assay of *pcch* protein from *P. chlororaphis* subsp. *chlororaphis* DSM 50083.

(A) GC-EI-MS spectrum of aristotelene produced by enzymatic synthesis with (i) PcchB+PcchC and (ii) PcchB+PcchC+PcchF in the presence of FPP and SAM. The absorption intensity of aristotelene produced in (ii) is assigned as 100%. The peak corresponding to aristotelene is highlighted in yellow. (B) The structure of the C_{16} terpenoid aristotelene. (C)-(D) GC-EI-MS analysis of VOCs produced in (i) assay (C) and (ii) assay (D) with a retention time of 13.3 min.

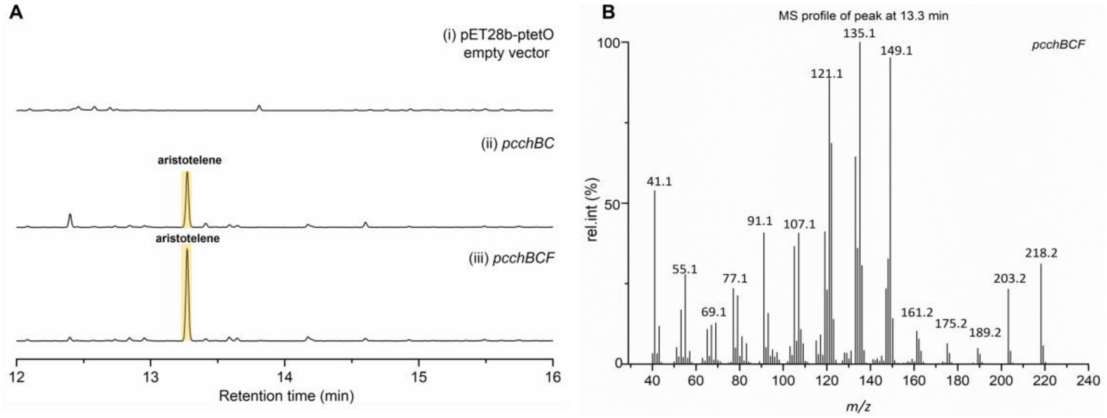

Figure S20. GC-EI-MS analyses of VOCs generated from the *in vivo* expression of *pcchBCF* from *P. chlororaphis* subsp. *chlororaphis* DSM 50083.

(A) GC-EI-MS spectrum showing aristotelene production by *E. coli* BAP1 strains on day 2 harboring: (i) the empty vector pET28-ptetO-GFPv2 (control), (ii) pET28-ptetO-gfpv2::*pcchBC*, and (iii) pET28-ptetO-gfpv2::*pcchBCF*. The peak intensity of aristotelene in trace (iii) is set as 100%. The aristotelene peak is highlighted in yellow. (B) GC-EI-MS trace of the VOC produced by strain (iii) at a retention time of 13.3 min.

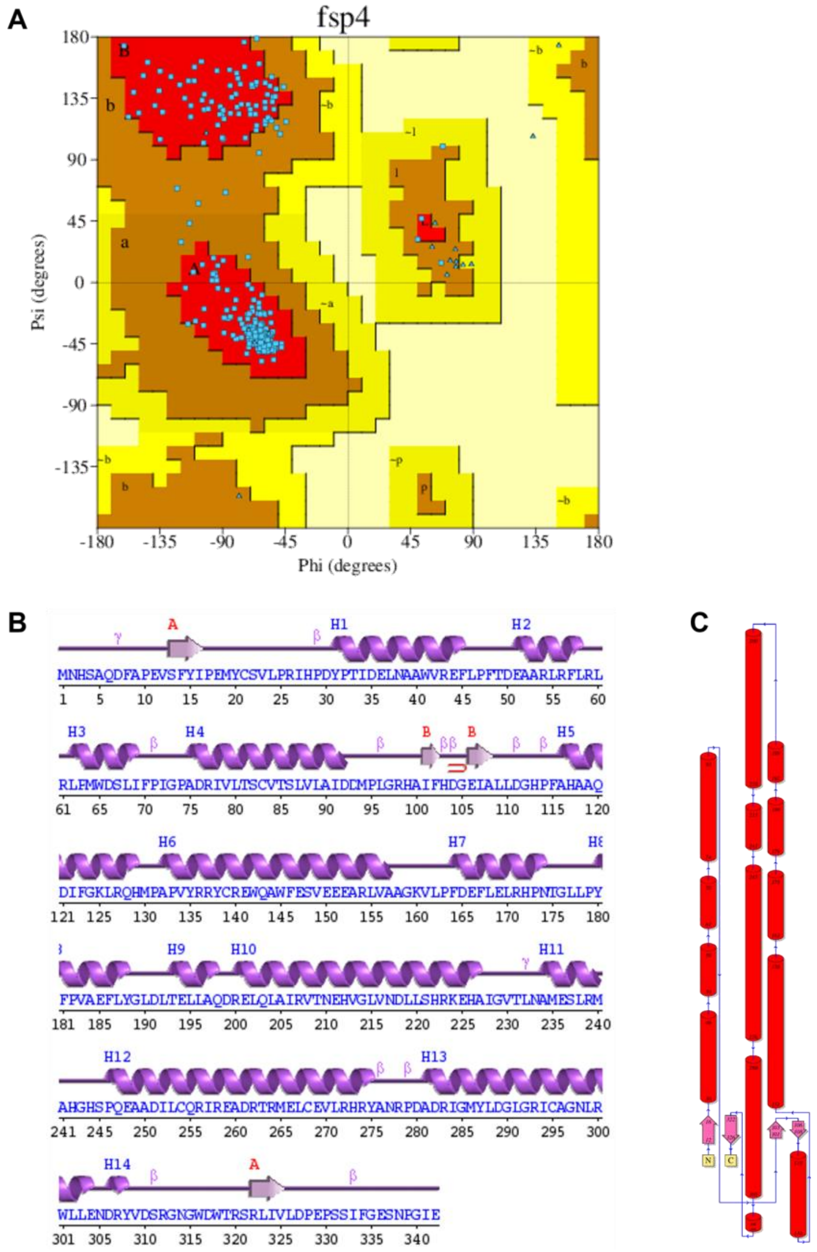

Figure S21. Protein structure analysis of protein model of terpene synthases PgrE generated by AlphaFold3.

(A) Ramachandran plot of protein model of terpene synthase PgrE generated by AlphaFold3.[7] Based on an analysis of 118 structures at a resolution of at least 2.0 Å and an R-factor no greater than 20.0, 281 residues are in 94.0% of the most favorable regions [A, B, L], indicating a good quality model. The overall average G-factor is 0.20, which is within the normal range, comparing backbone bond lengths and angles to the ideal values derived from small molecule data by Engh & Huber (1991). (B) The secondary structure of the protein model of the terpene synthase PgrE generated by AlphaFold3 includes 14 helices, 4 strands, 2 sheets, 32 helix–helix interactions, and motifs with 1 beta hairpin, 11 beta turns, and 2 gamma turns. C. Topology of the protein model of the terpene synthase PgrE generated by AlphaFold3. Protein structure analyses are provided by PDBsum web server (https://www.ebi.ac.uk/pdbsum)[8].

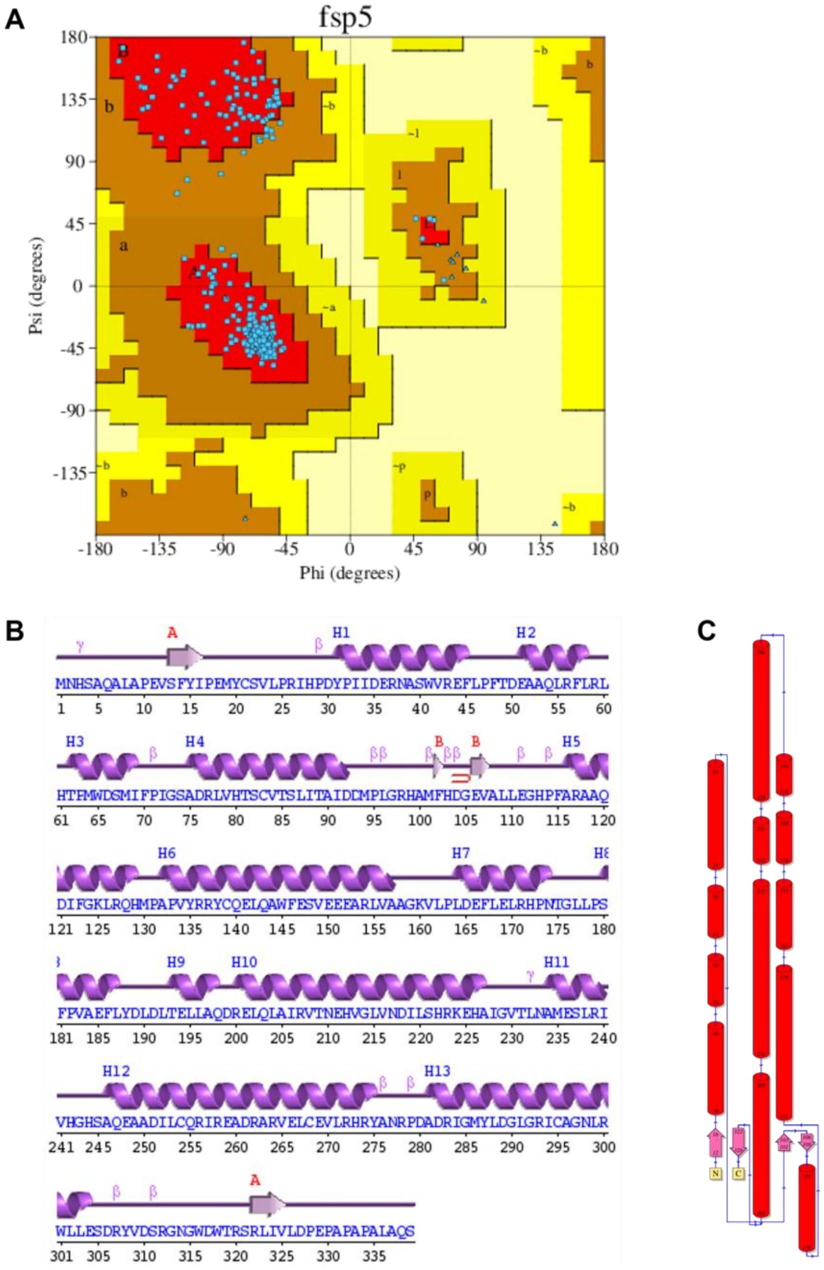

Figure S22. Protein structure analysis of protein model of terpene synthases PchlO6_6041 generated by AlphaFold3.

(**A**) Ramachandran plot of protein model of PchlO6_6041 generated by AlphaFold3.[7] Based on an analysis of 118 structures of resolution of at least 2.0 Å and an R-factor no greater than 20.0, 281 residues are in 94.7% of the most favorable regions [A, B, L], indicating a good quality model. The overall average G-factor is 0.21, which is within the normal range, comparing backbone bond lengths and angles to the ideal values derived from small molecule data by Engh & Huber (1991). (**B**) The secondary structure of the protein model of PchlO6_6041 generated by AlphaFold3 includes 13 helices, 4 strands, 2 sheets, 30 helix–helix interactions, and motifs with 1 beta hairpin, 13 beta turns, and 2 gamma turns. **C**. Topology of the protein model of the PchlO6_6041 generated by AlphaFold3. Protein structure analyses are provided by PDBsum web server (https://www.ebi.ac.uk/pdbsum)[8].

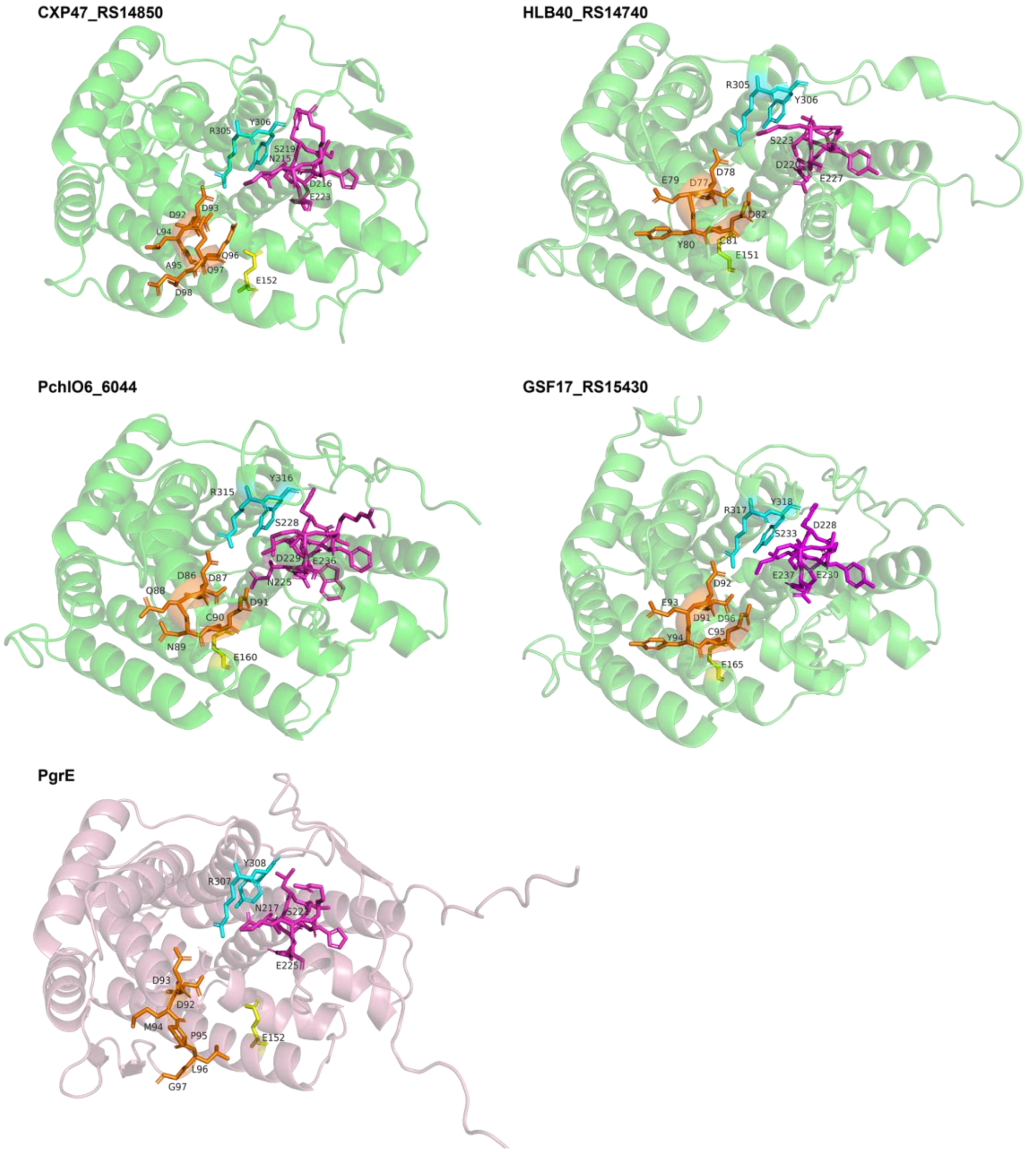

Figure S23. Protein models of group A terpene synthases generated by AlphaFold3.

The conserved motifs of each group of terpene synthases defined in this study are highlighted, displayed as sticks, and the corresponding amino acid residues are indicated. Orange, magenta, and yellow represent Asp-rich motifs, NSE triad, and conserved E, respectively, and blue represents RY dimer. Only one terpene synthase is shown for each TS group. CXP47_RS14850 from *P. chlororaphis* Lzh-T5, PgrE from *P. grimontii* DSM 17515, HLB40_RS14740 from *P. chlororaphis* qlu-1, PchlO6_6044 from *P. chlororaphis* O6 and GSF17_RS15430 from *P. chlororaphis* subsp. *aurantiaca* zm-1 were classified in group A, B, C, D and E, respectively.

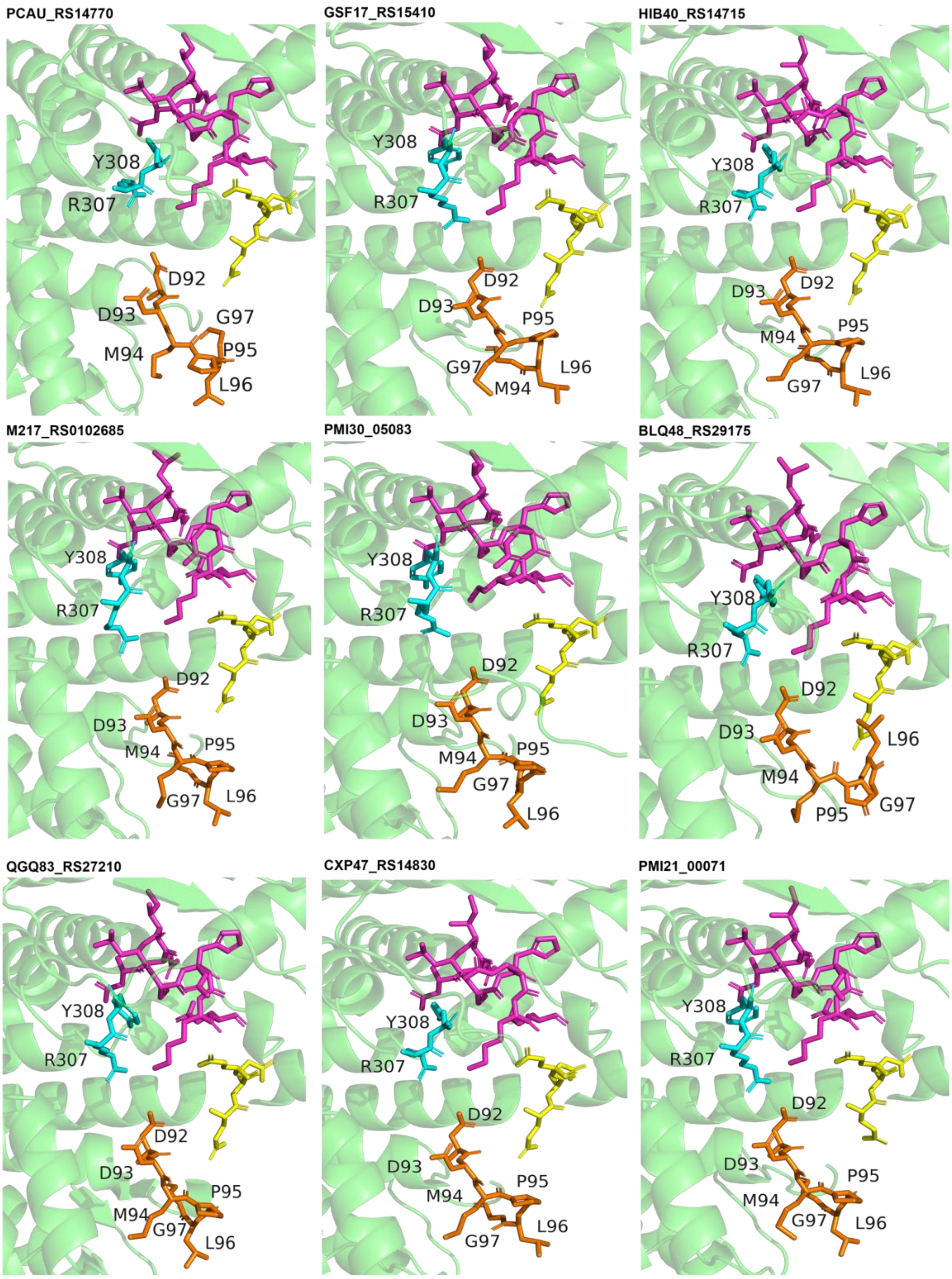

Figure S24. Protein models of group B terpene synthases generated by AlphaFold3.

The partial zoom-in views of potential active-site pockets are shown. The conserved motifs of each group of terpene synthases defined in this study are highlighted, displayed as sticks, and the corresponding amino acid residues are indicated. Orange, magenta, and yellow represent Asp-rich motifs, NSE triad, and conserved E, respectively, and blue represents RY dimer.

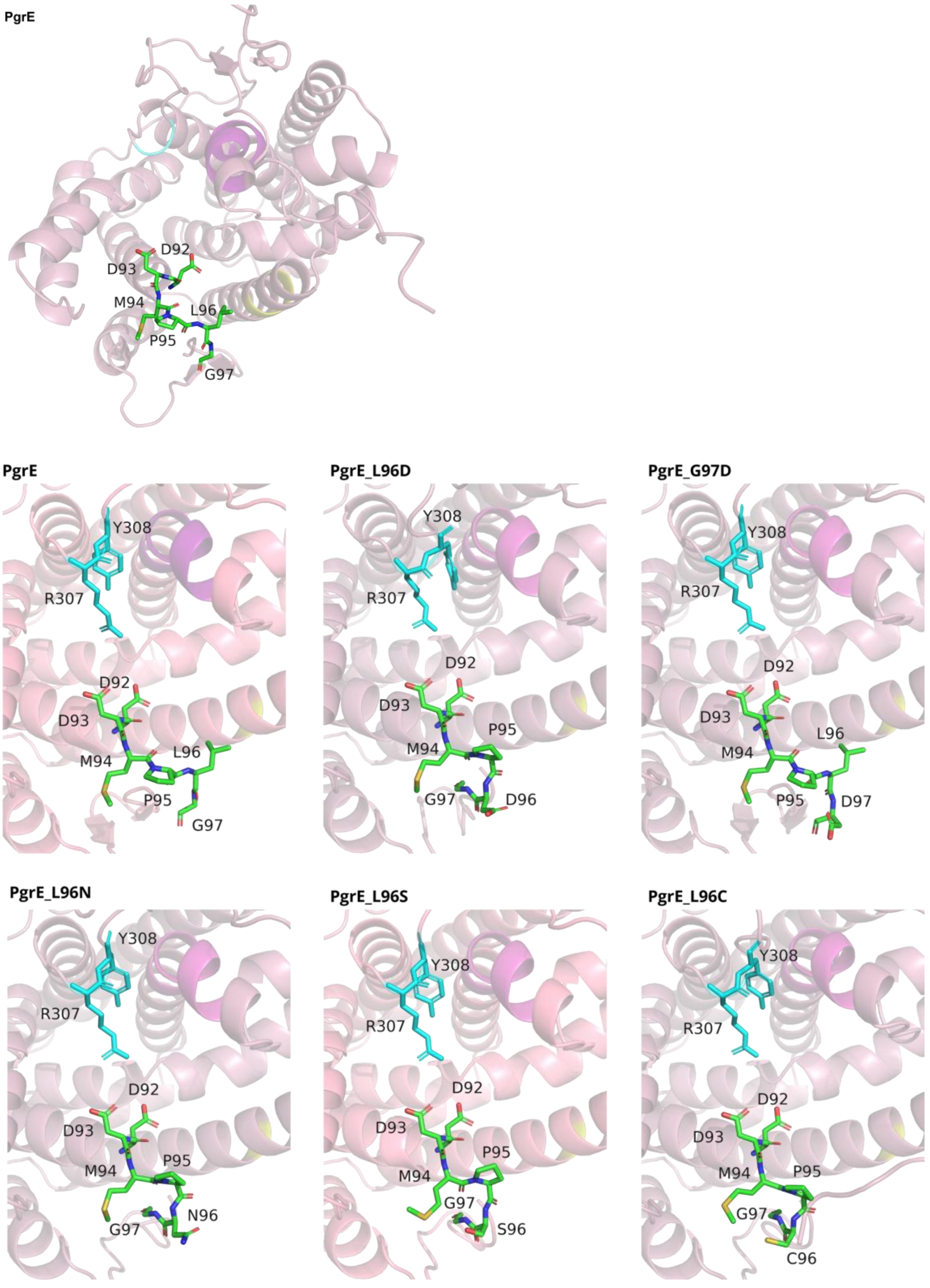
PgrE
D92
D93
M94
L96
P95
G97
PgrE
Y308
R307
D92
D93
L96
M94
P95
G97
PgrE_L96D
Y308
R307
D92
D93
P95
M94
G97
D96
PgrE_G97D
Y308
R307
D92
D93
L96
M94
P95
D97
PgrE_L96N
Y308
R307
D92
D93
P95
M94
N96
G97
PgrE_L96S
Y308
R307
D92
D93
P95
M94
G97
S96
PgrE_L96C
Y308
R307
D92
D93
M94
P95
G97
C96

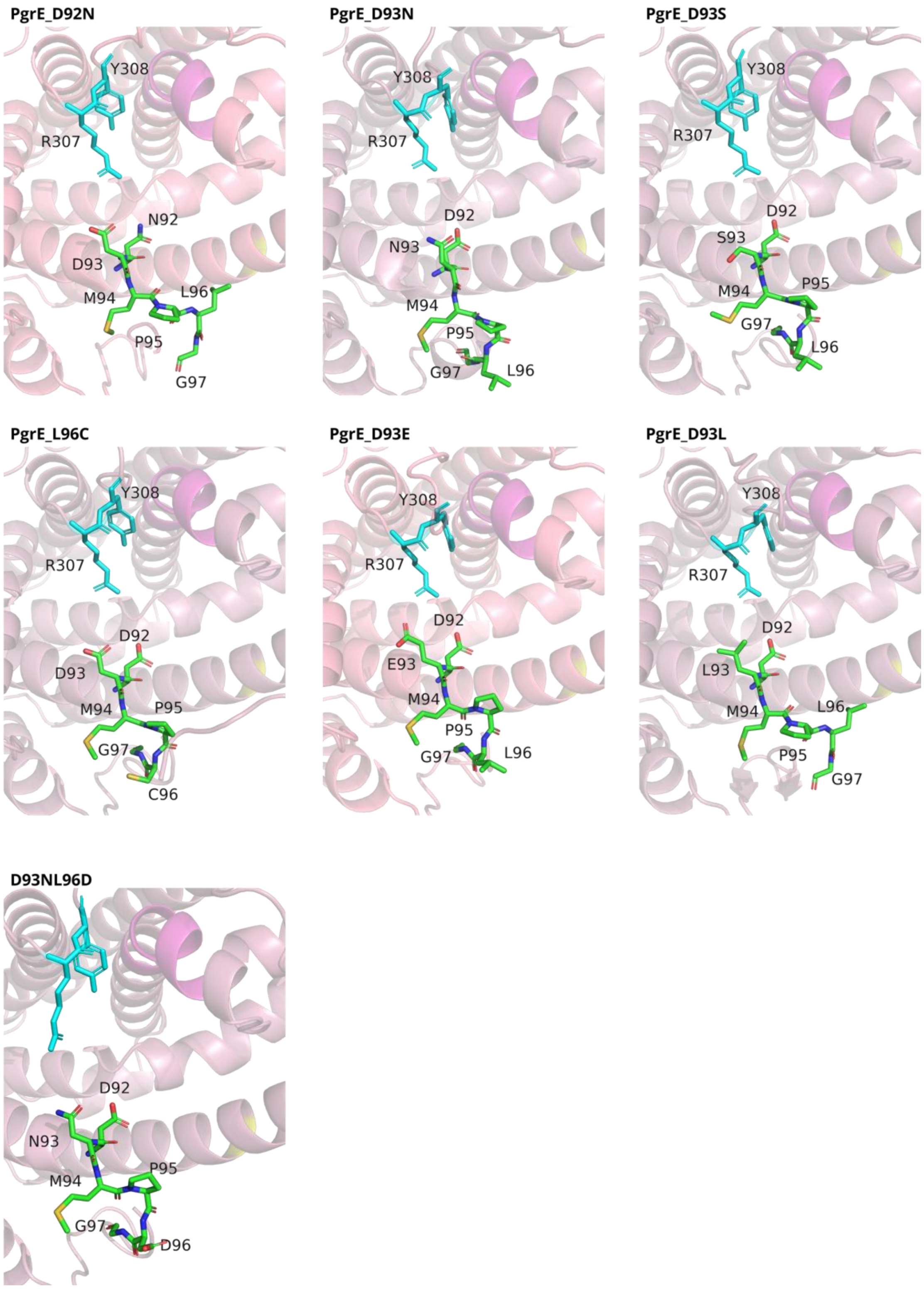

Figure S25. Protein models of PgrE terpene synthase mutants generated by AlphaFold3. The partial zoom-in views of potential active-site pockets are shown. The conserved motifs of each group of terpene synthases defined in this study are highlighted, and the corresponding amino acid residues are indicated. Magenta and yellow represent NSE triad, and conserved E, respectively, and blue represents RY dimer. Asp-rich motif and RY dimer are shown as sticks with corresponding amino acid residue indicated.

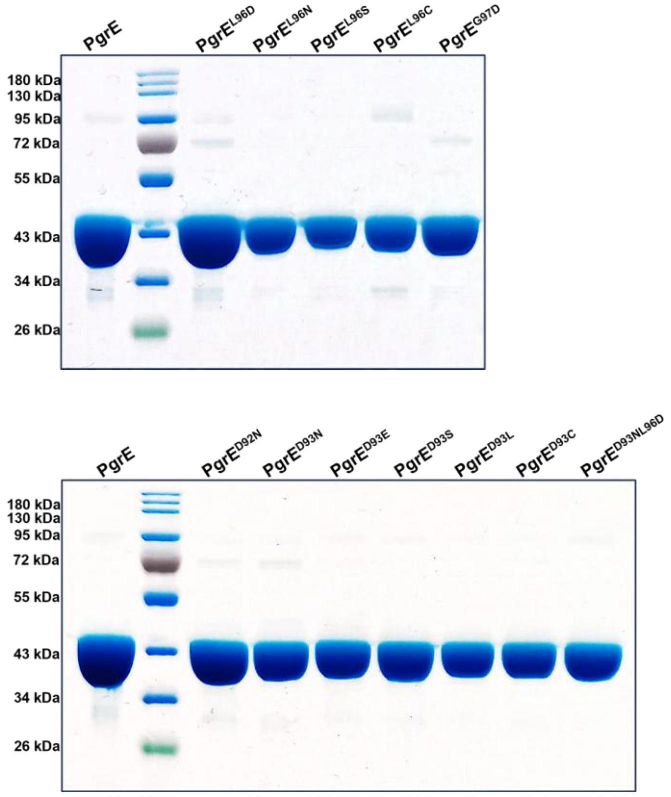

Figure S26. SDS-PAGE analyses of the purified terpene synthase PgrE and its mutants generated in this study.

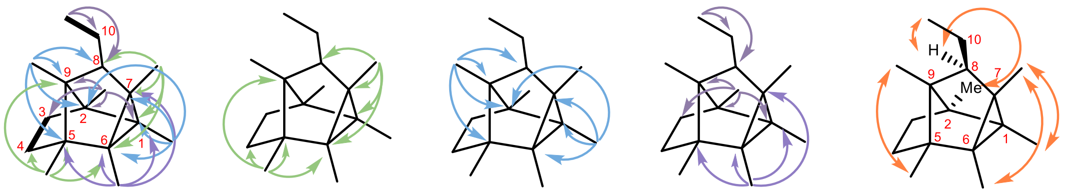

grimophan (**7**)

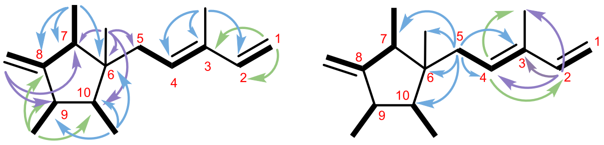

sodoritriene (**8**)

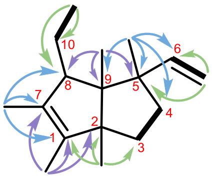

bicycloprechlororaphen (**11**)

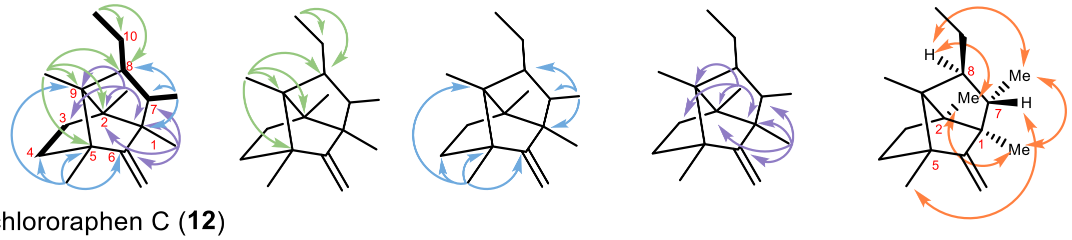

chlororaphen C (**12**)

— ^2J- & ^3J-H,H- correlations (^{1}H-COSY)

^2J- & ^3J-H,C- correlations (HMBC)

NOE H,H- correlations (NOESY)

Figure S27: Structure assignment of grimophan (**7**), sodoritriene (**8**), bicycloprechlororaphen (**11**) and chlororaphen C (**12**).

Table S8. ^1H and ^{13}C NMR data (*δ* in ppm, *J* in Hz) of compound 7 in C_6D_6* and $CDCl_3$.

No.	Types	7			
		δ_C*	δ_H* (*J* in Hz)	δ_C	δ_H (*J* in Hz)
1	C	29.69	-	29.48	-
2	C	54.47	-	54.26	-
3	CH_2	29.68	1.17 *m*<br>1.45 *m*	29.42	1.10 *m*<br>1.36 *m*
4	CH_2	31.17	1.18 *m*<br>1.42 *m*	30.91	1.11 *m*<br>1.33 *m*
5	C	53.30	-	53.08	-
6	C	32.36	-	31.76	-
7	C	28.89	-	28.66	-
8	CH	53.21	1.22 *dd* (7.5/3.3)	52.91	1.14 *dd* (7.4/3.4)
9	C	51.99	-	51.76	-
10	CH_2	20.73	1.32 *dqnt* (13.8/7.4)<br>1.40 *dqd* (13.6/7.4/3.1)	20.42	1.29 *dqnt* 13.7/7.4)<br>1.37 *dqd* (13.7/7.8/3.0)
1-CH_3	CH_3	4.72	0.78 *s*	4.66	0.75 *s*
2-CH_3	CH_3	14.13	0.73 *s*	14.04	0.66 *s*
5-CH_3	CH_3	16.31	0.84 *s*	16.18	0.78 *s*
6-CH_3	CH_3	5.30	0.80 *d* (0.5)	5.23	0.77 *d* (0.5)
7-CH_3	CH_3	9.72	0.99 *s*	9.68	0.97 *s*
9-CH_3	CH_3	7.46	0.68 *s*	7.39	0.65 *s*
10-CH_3	CH_3	16.28	0.98 *dd* (7.8/7.3)	16.17	0.92 *dd* (7.8/7.4)

NMR data were recorded at ambient temperature at 600.1 MHz for ^1H and 150.9 MHz for ^{13}C. Chemical shifts *δ* are given in ppm relative to TMS. Residual proton signals as well as ^{13}C signals of the NMR solvents were used as reference (^1H: δ_H 7.160 ppm for residual C_6HD_5 and ^1H: δ_H 7.260 ppm for residual $CHCl_3$, ^{13}C: δ_C 128.06 ppm for C_6D_6 and ^{13}C: δ_C 77.16 ppm for $CDCl_3$). Coupling constants are given in Hertz and determined assuming first-order spin-spin coupling unless otherwise stated.

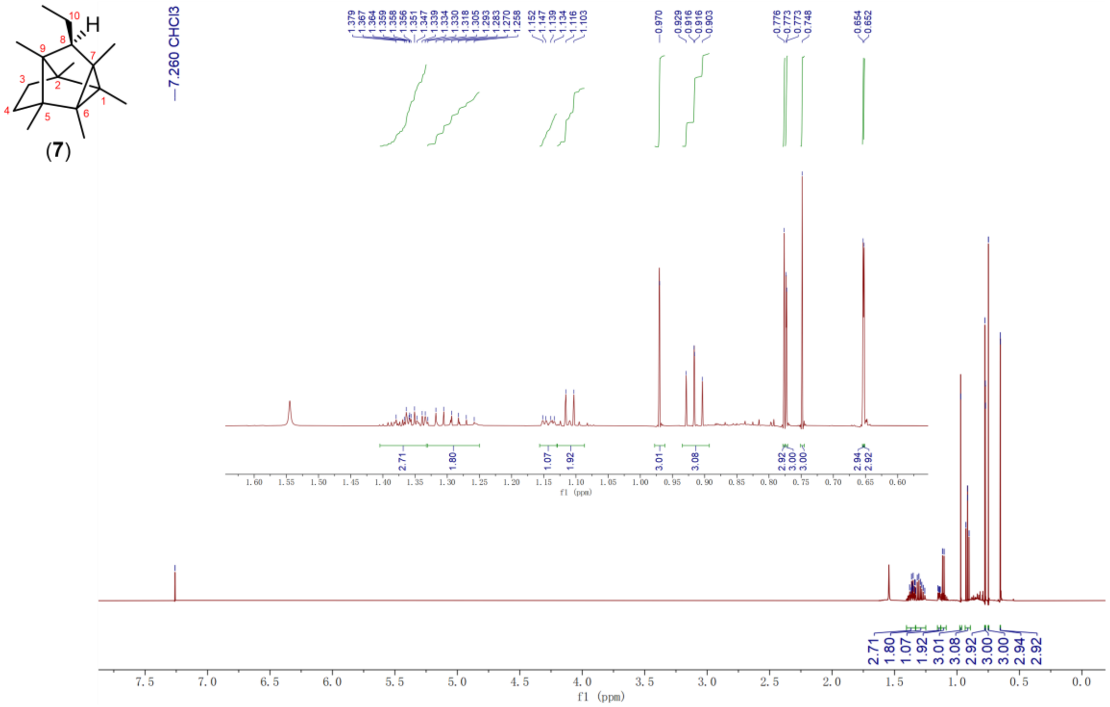

Figure S28. ^{1}H NMR spectrum of compound 7 in $CDCl_3$.

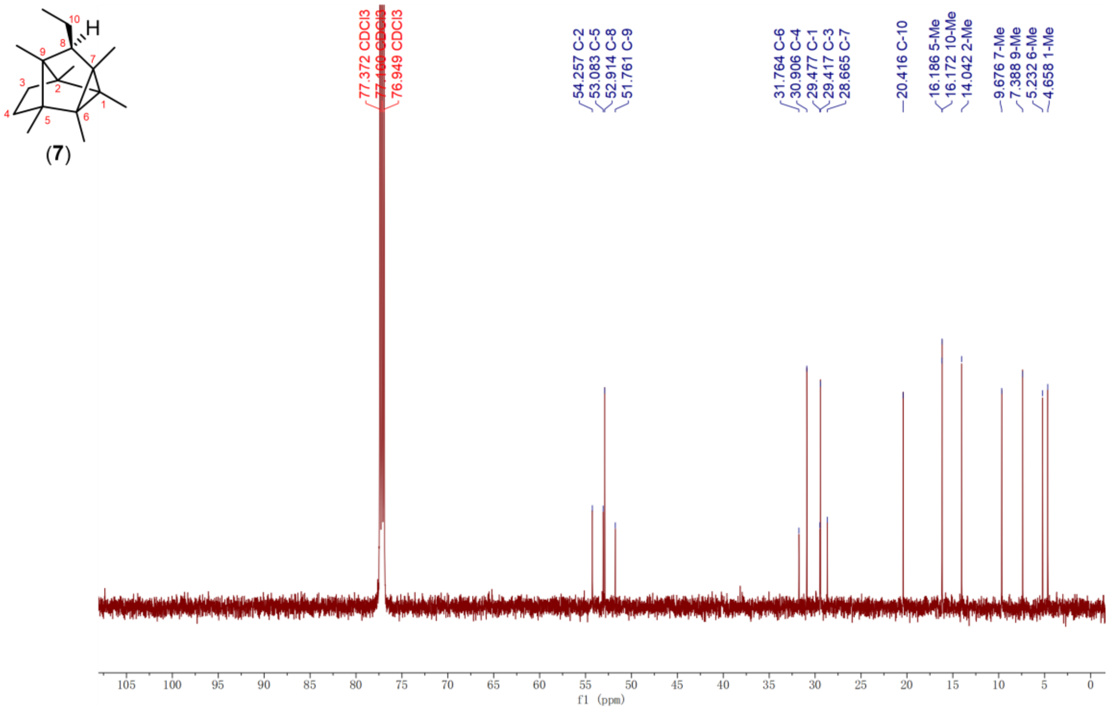

Figure S29. ^{13}C NMR spectrum of compound 7 in $CDCl_3$.

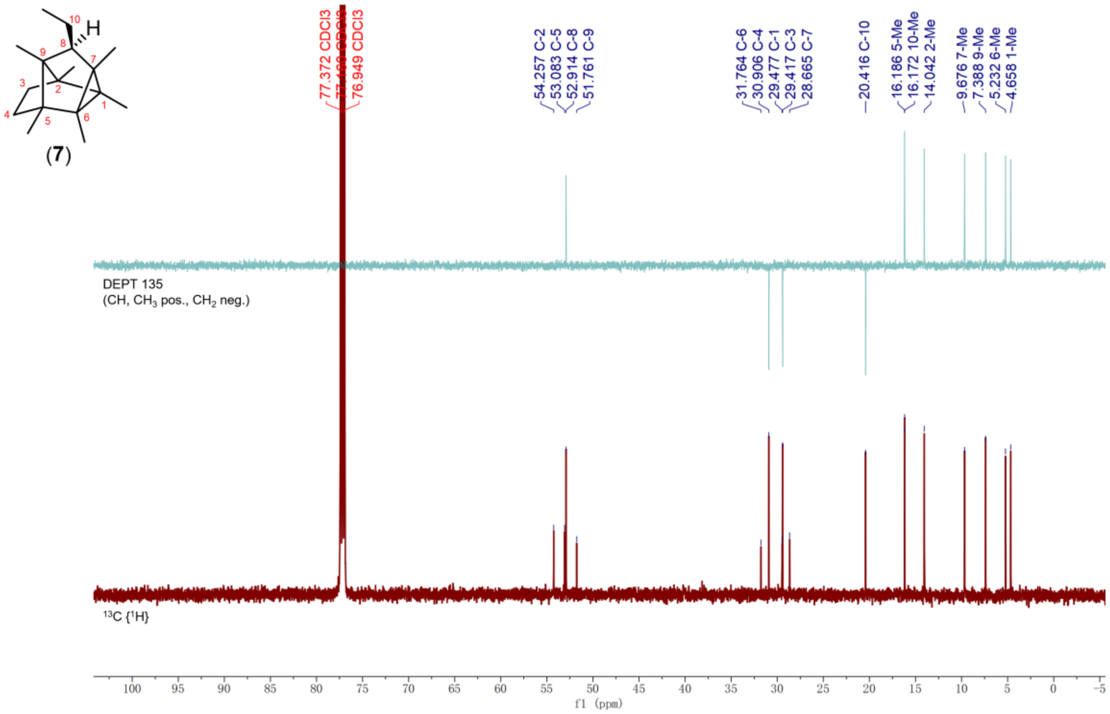

Figure S30. $^{13}C\{^1H\}$ and DEPT135 NMR spectrum of compound 7 in $CDCl_3$.

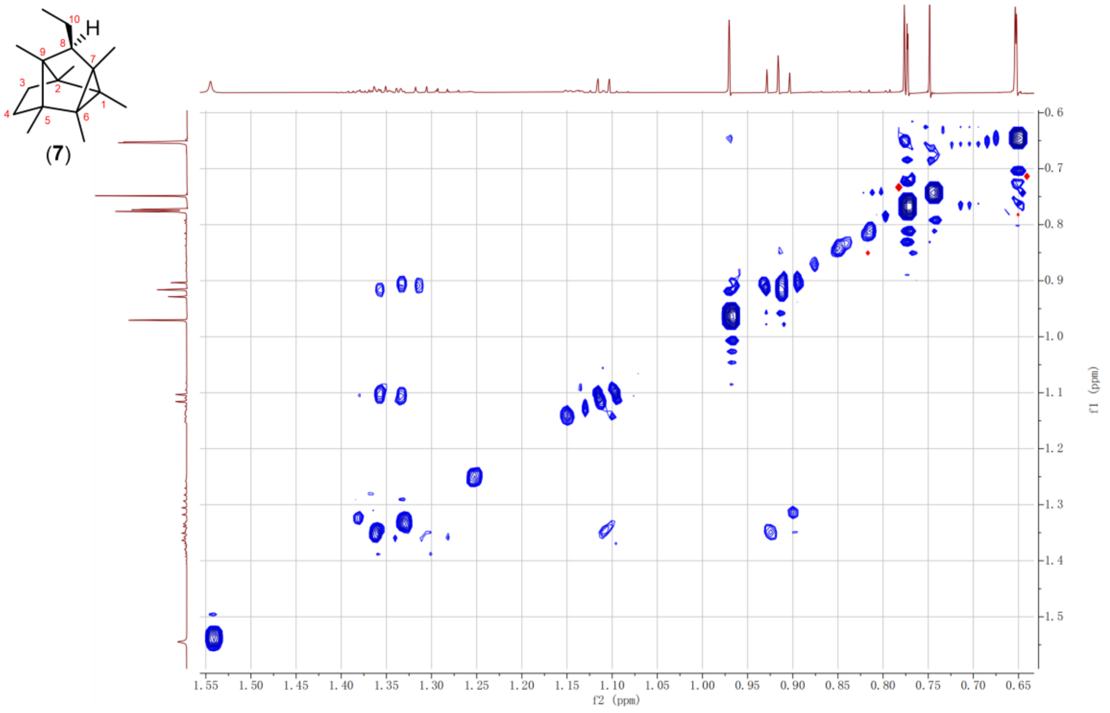

Figure S31. COSY spectrum of compound 7 in $CDCl_3$.

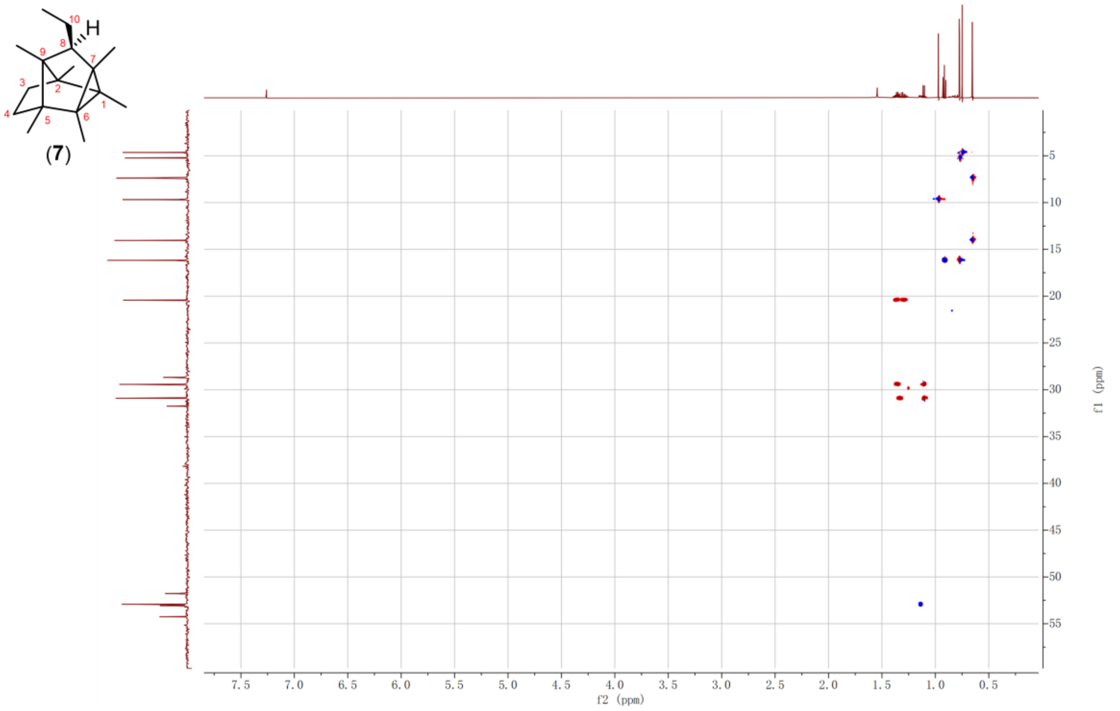

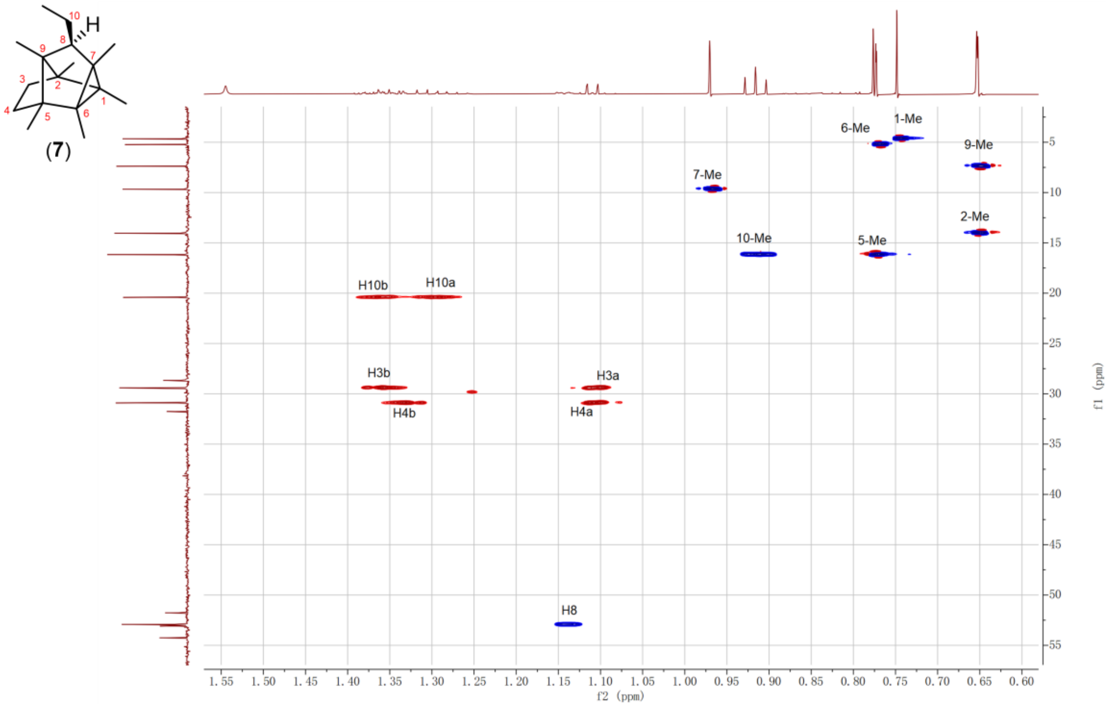

Figure S32. HSQC spectrum of compound 7 in $CDCl_3$.

Zoomed-in view of the decisive part of the HSQC spectrum (below) of compound **7** in $CDCl_3$.

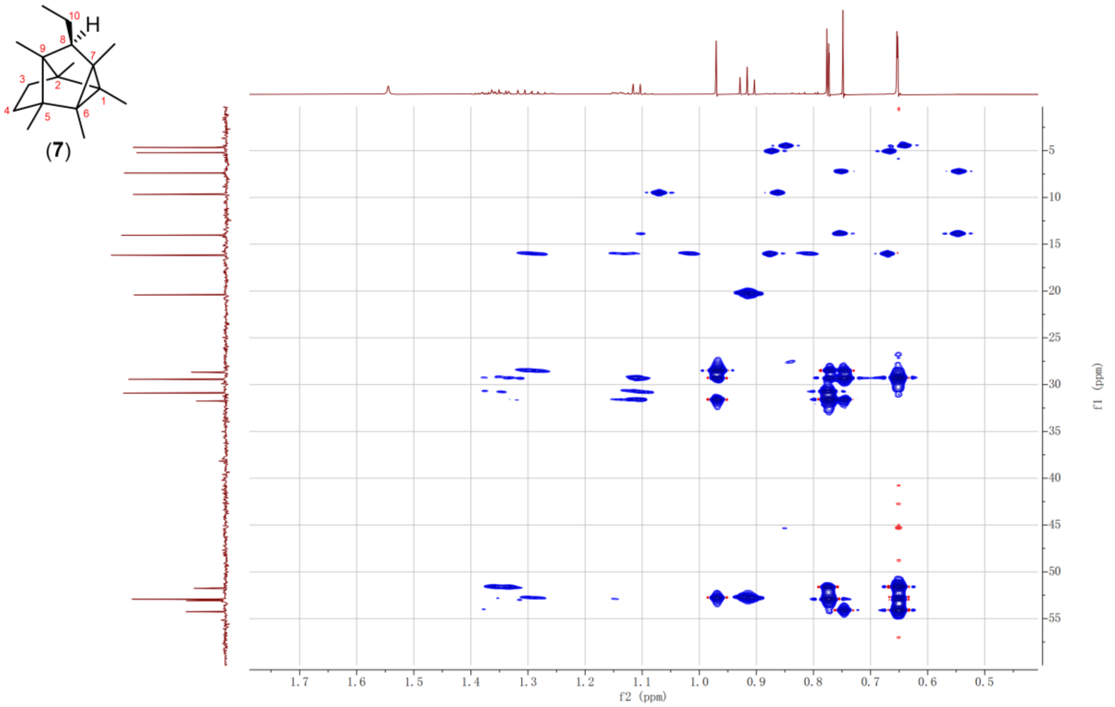

Figure S33. HMBC spectrum of compound 7 in $CDCl_3$.

The decisive part of the spectrum is zoomed in. The corresponding HMBC correlations (colored arrows) for the structural elucidation of compound **7** are shown in Figure S27.

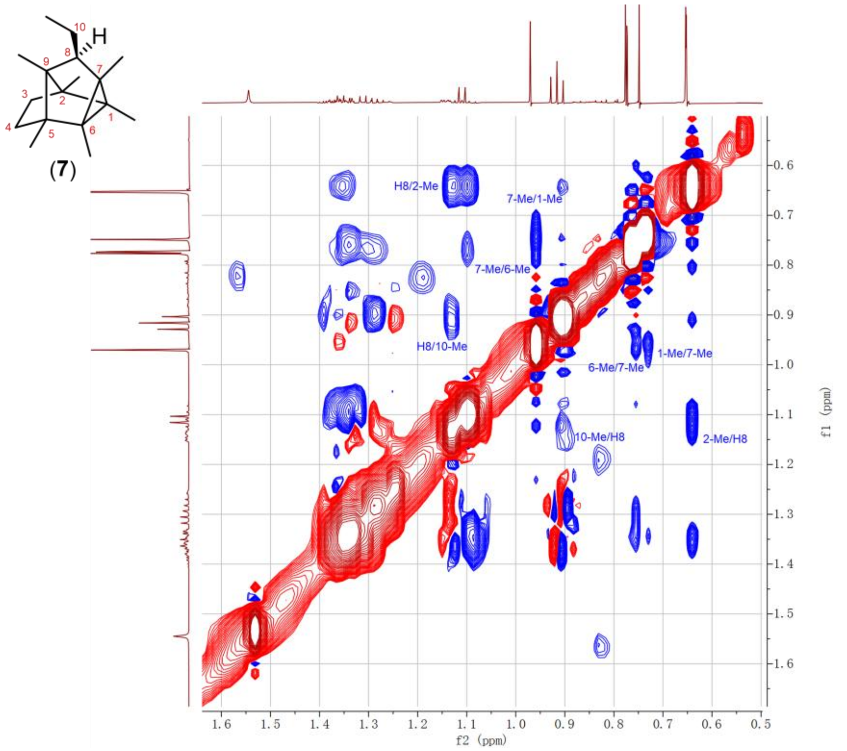

Figure S34. NOESY spectrum of compound 7 in $CDCl_3$.

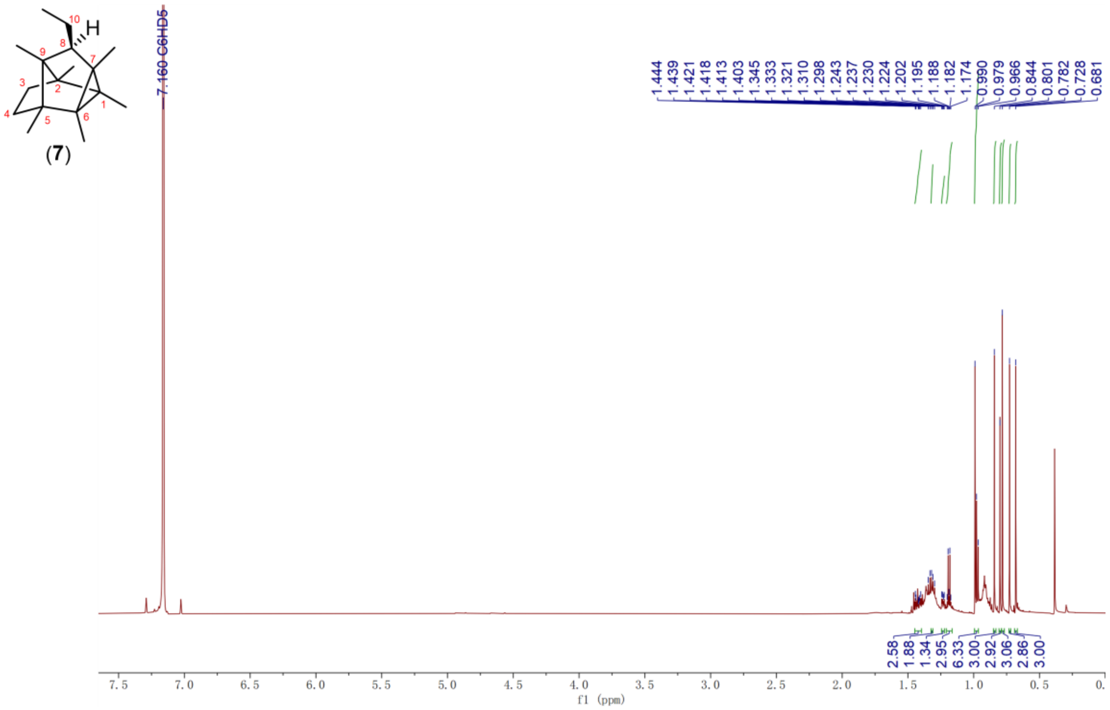

Figure S35. ^{1}H NMR spectrum of compound 7 in C_6D_6.

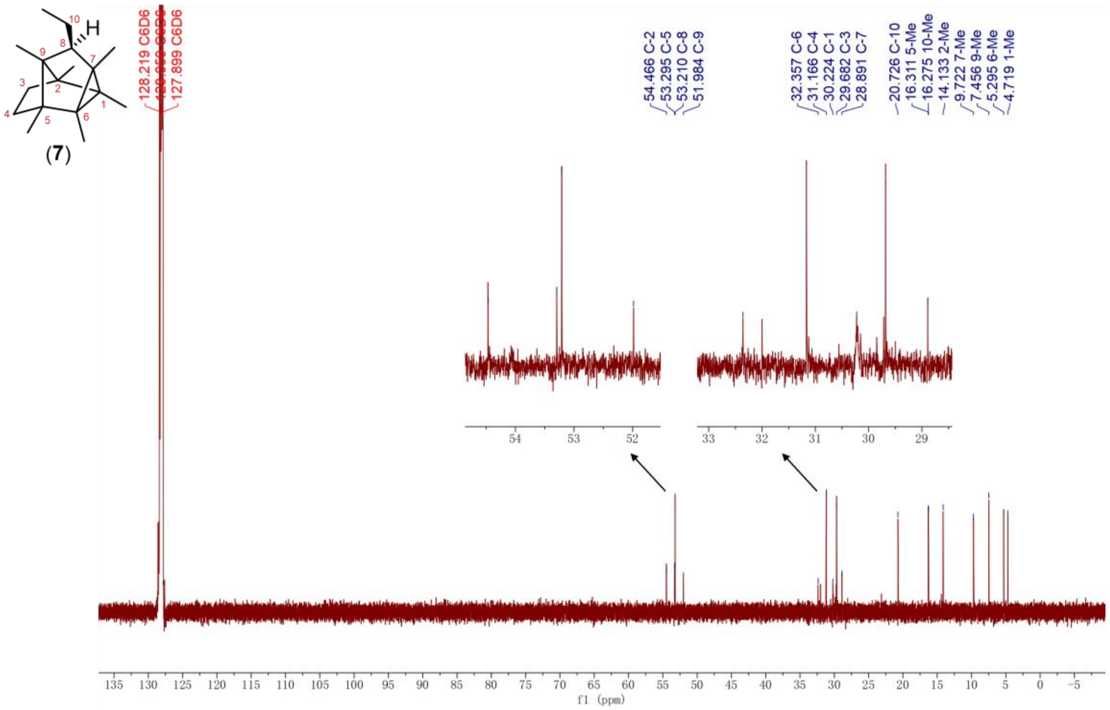

Figure S36. ^{13}C NMR spectrum of compound 7 in C_6D_6.

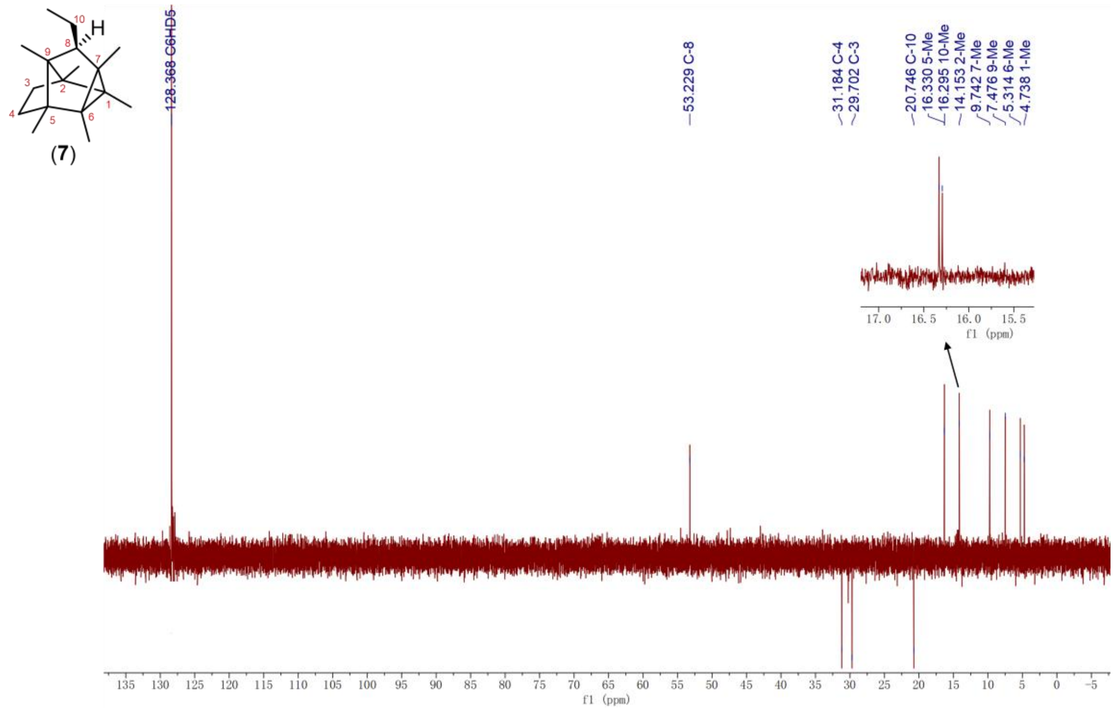

Figure S37. DEPT135 ^{13}C NMR spectrum of compound 7 in C_6D_6.

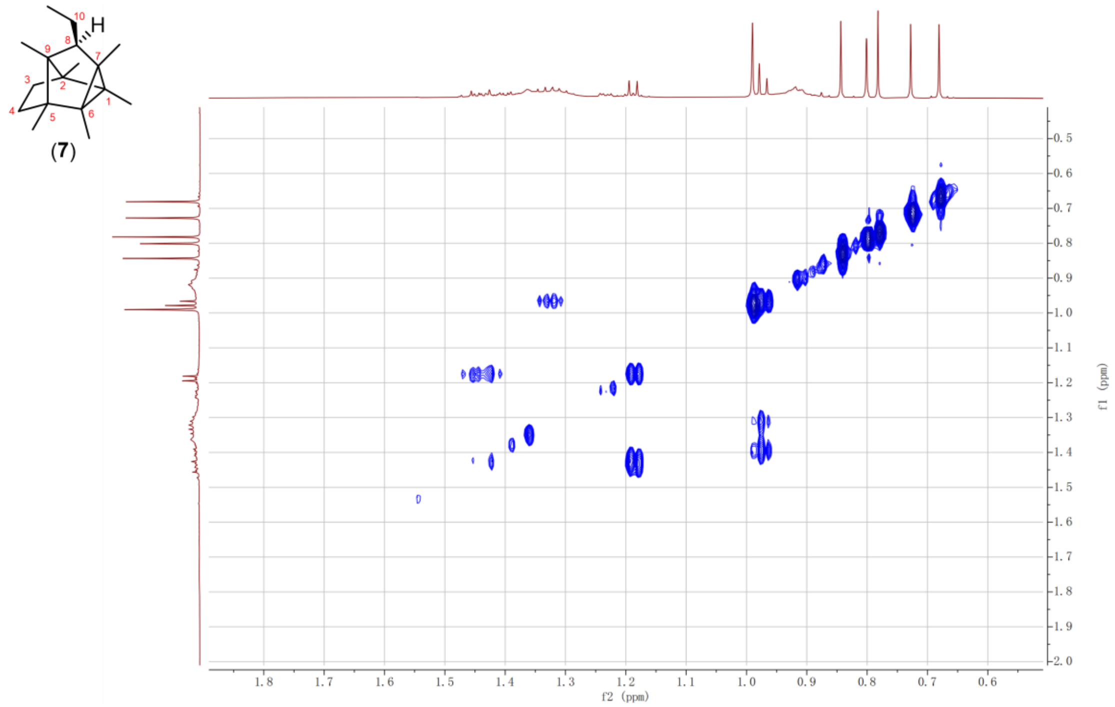

Figure S38. COSY spectrum of compound 7 in C_6D_6.

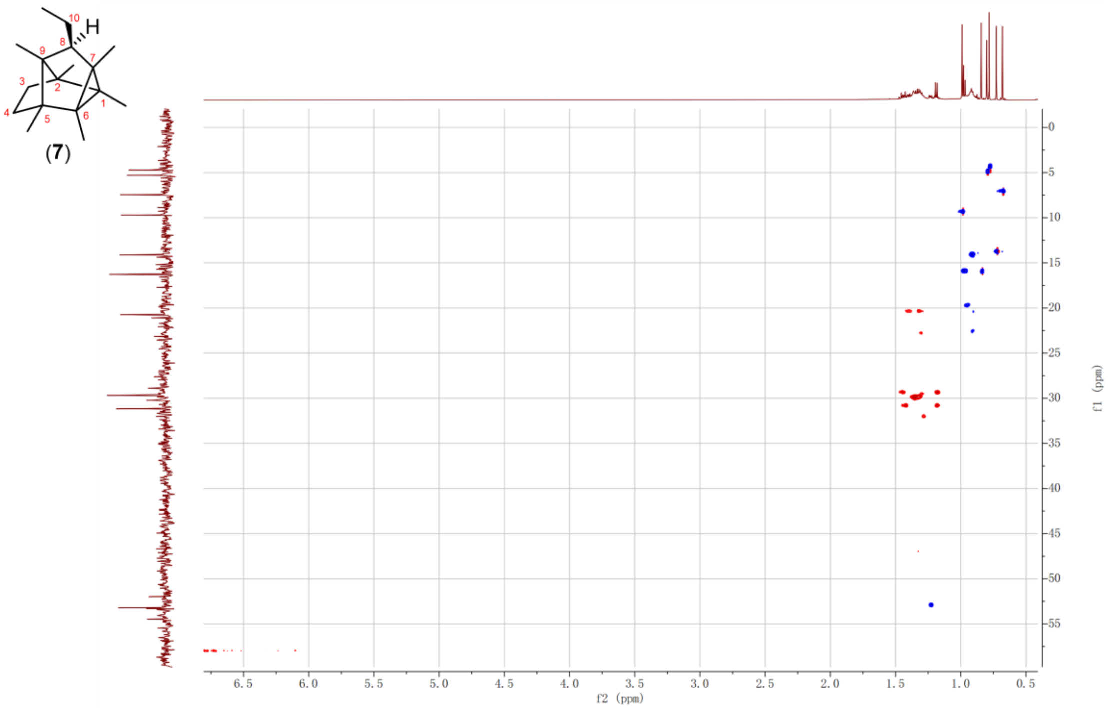

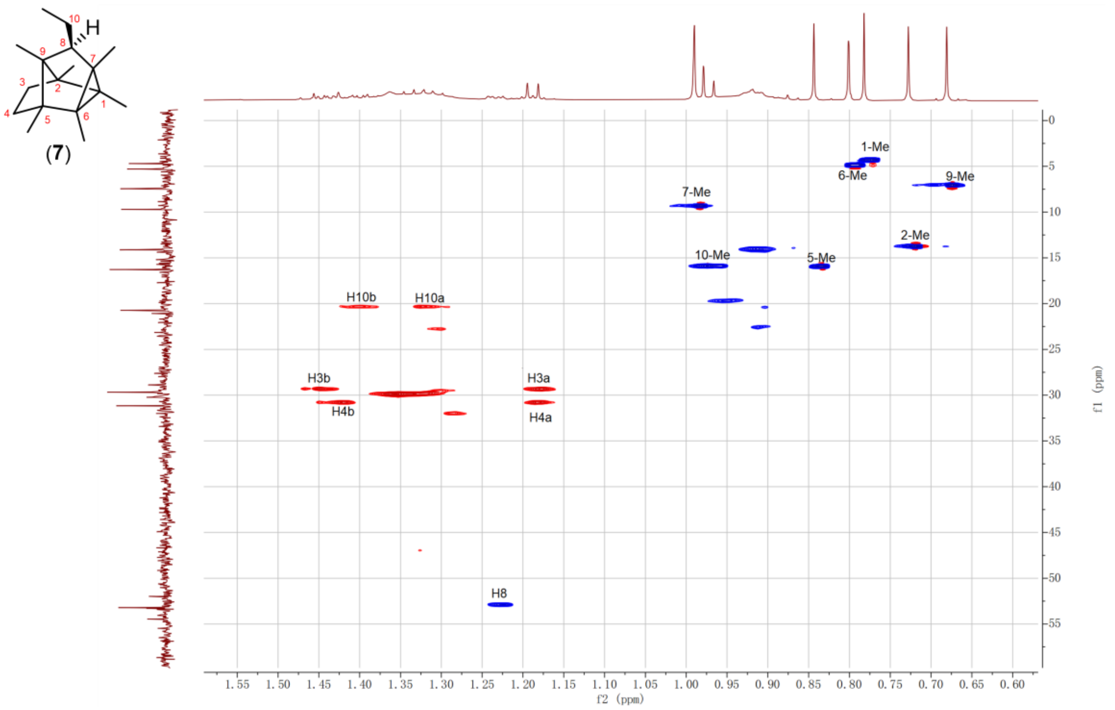

Figure S39. HSQC spectrum of compound 7 in C_6D_6.

Zoomed-in view of the decisive part of the HSQC spectrum (below) of compound **7** in C_6D_6.

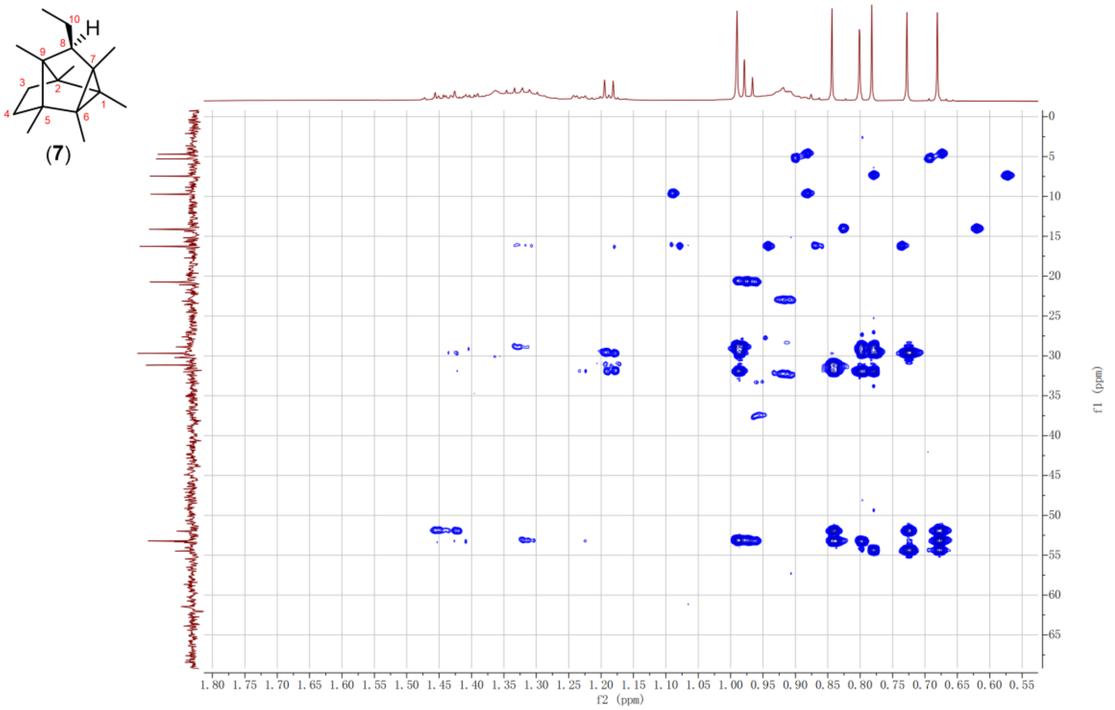

Figure S40. HMBC spectrum of compound 7 in C_6D_6.

The decisive part of the spectrum is zoomed in. The corresponding HMBC correlations (colored arrows) for the structural elucidation of compound **7** are shown in Figure S27.

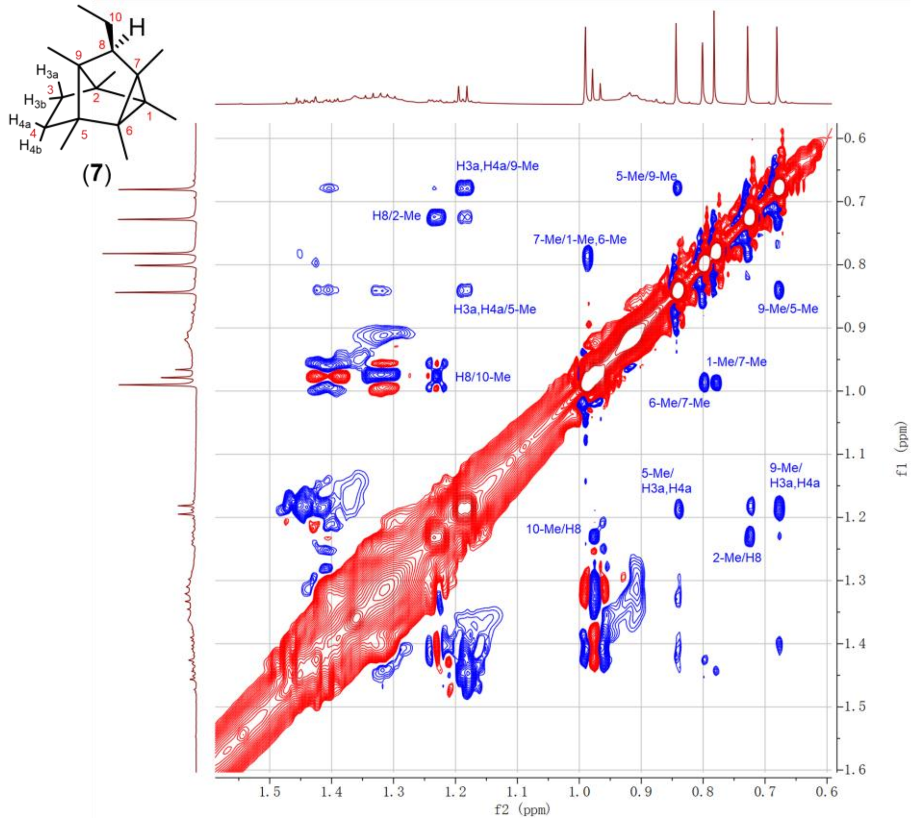

Figure S41. NOESY spectrum of compound 7 in C_6D_6.

Table S9. ^1H and ^{13}C NMR data (δ in ppm, J in Hz) of compound 8 in $CDCl_3$.

No.	Types	8	
		δ_C	δ_H (J in Hz)
1	$=CH_2$	110.6	4.94 *d* (10.7)<br>5.08 *d* (17.4)
2	CH	142.3	6.38 *dd* (17.4/10.4)
3	C	135.5	-
4	CH	129.7	5.53 *t* (8.0)
5	CH_2	35.6	2.13 *d* (8.0)
6	C	46.4	-
7	CH	45.9	2.22 *m*
8	C	161.9	-
9	CH	43.5	1.99 *dqdd* (10.6/6.8/2.7/2.2)
10	CH	47.0	1.26 *dq* (10.6/6.8)
3-CH_3	CH_3	12.0	1.75 *dt* (1.4/0.7)
6-CH_3	CH_3	15.0	0.57 *s*
7-CH_3	CH_3	11.9	0.93 *d* (6.9)
8=CH2	$=CH_2$	103.7	4.75 *td* (2.7/0.8)<br>4.80 *ddd* (3.1/2.2/0.9)
9-CH_3	CH_3	19.2	1.07 *d* (6.8)
10-CH_3	CH_3	12.9	0.88 *d* (6.8)

NMR data were recorded at ambient temperature operating at 600.1 MHz for ^1H and 150.9 MHz for ^{13}C. Chemical shifts δ are given in ppm relative to TMS. The solvent signals were used as reference (^1H: δ_H 7.260 ppm for residual $CHCl_3$, ^{13}C: δ_C 77.16 ppm for $CDCl_3$). Coupling constants are given in Hertz and determined assuming first-order spin-spin coupling unless otherwise stated.

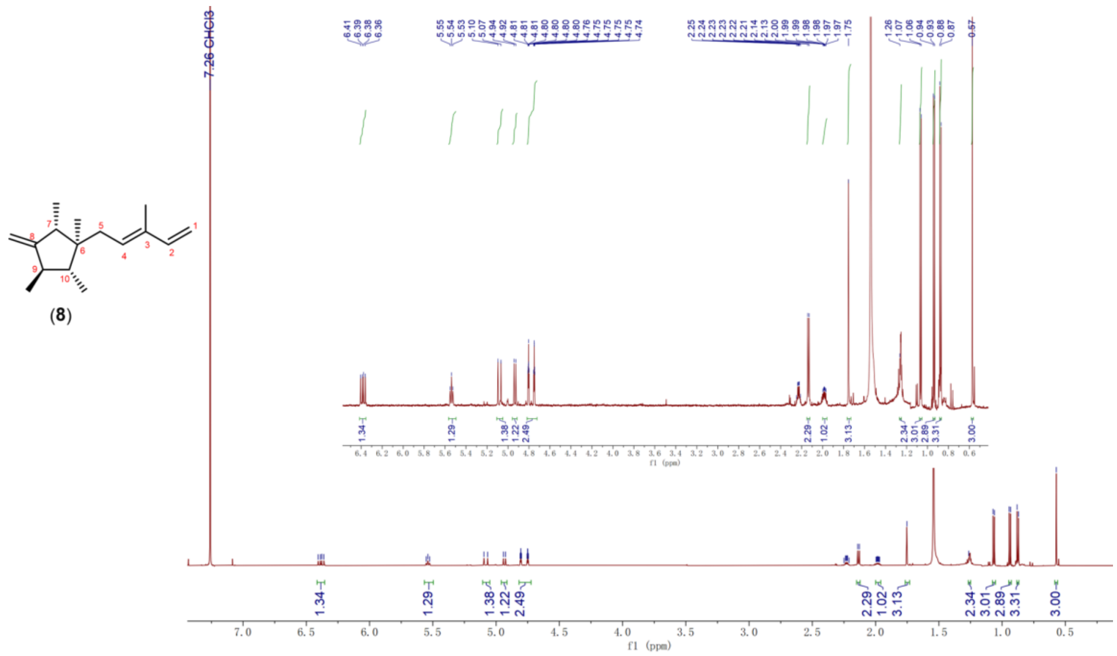

Figure S42. ^{1}H NMR spectrum of compound 8 in $CDCl_3$.

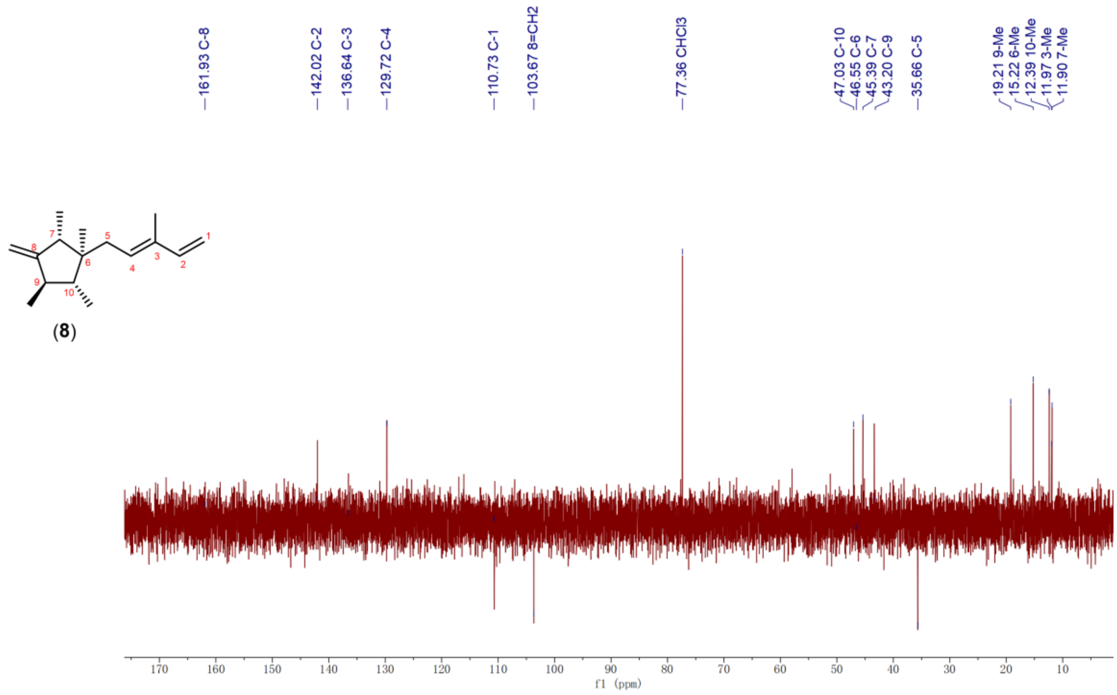

Figure S43. DEPT135 ^{13}C NMR spectrum of compound 8 in $CDCl_3$.

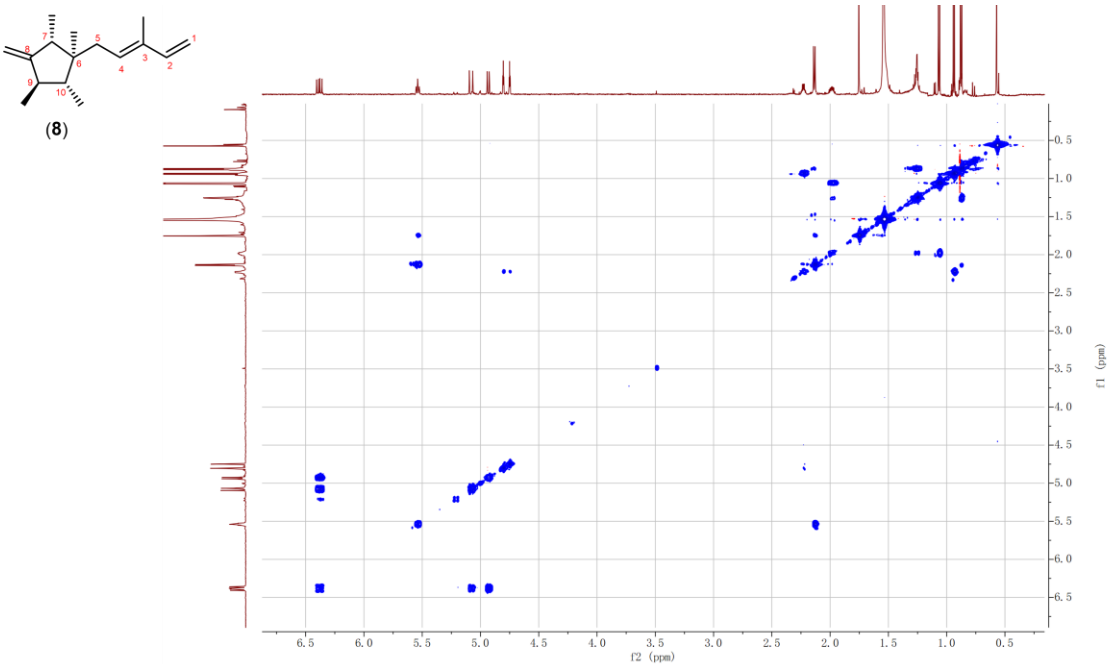

Figure S44. COSY spectrum of compound 8 in $CDCl_3$.

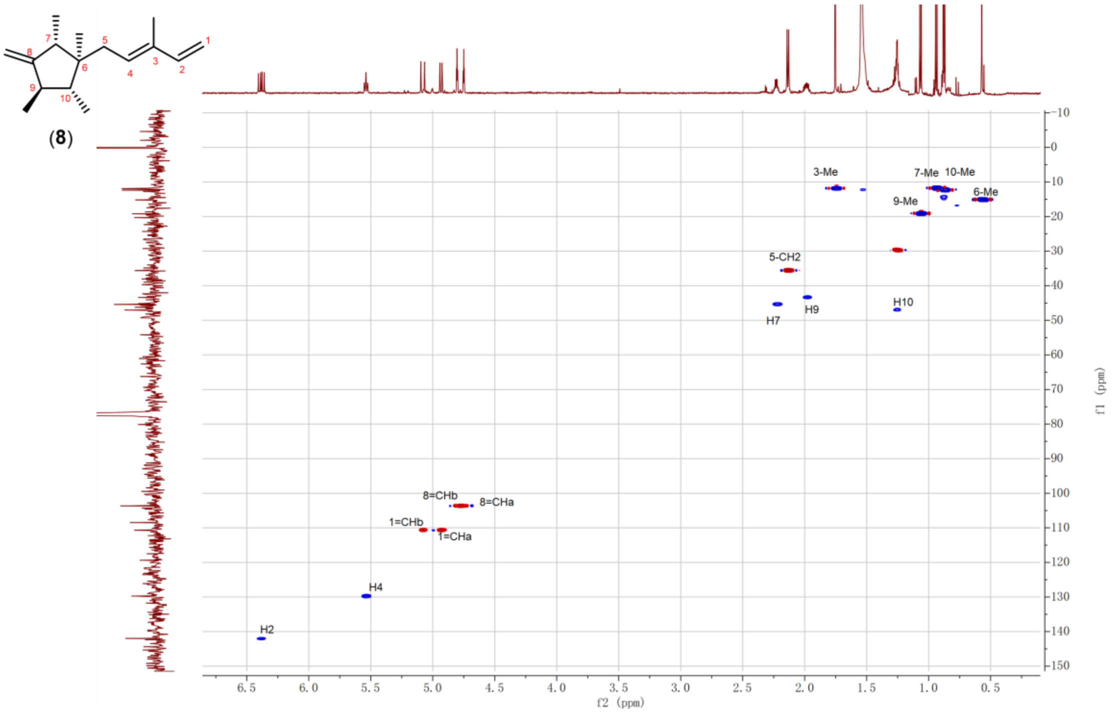

Figure S45. HSQC spectrum of compound 8 in $CDCl_3$.

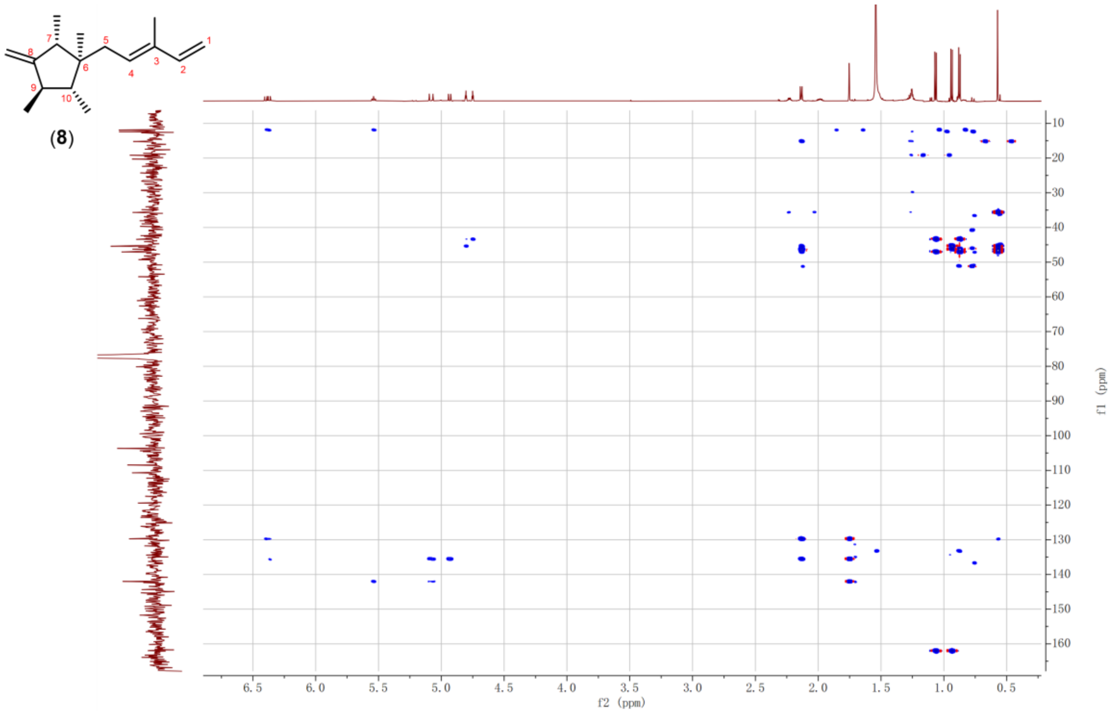

Figure S46. HMBC spectrum of compound 8 in $CDCl_3$.

The corresponding HMBC correlations (colored arrows) for the structural elucidation of compound **8** are shown in Figure S27.

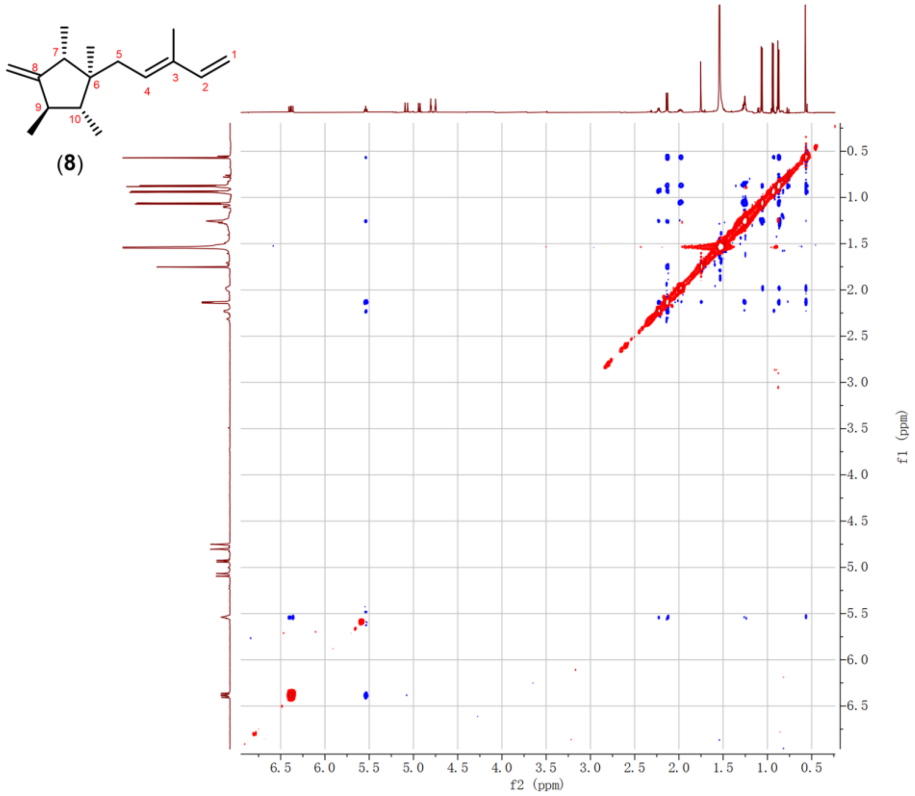

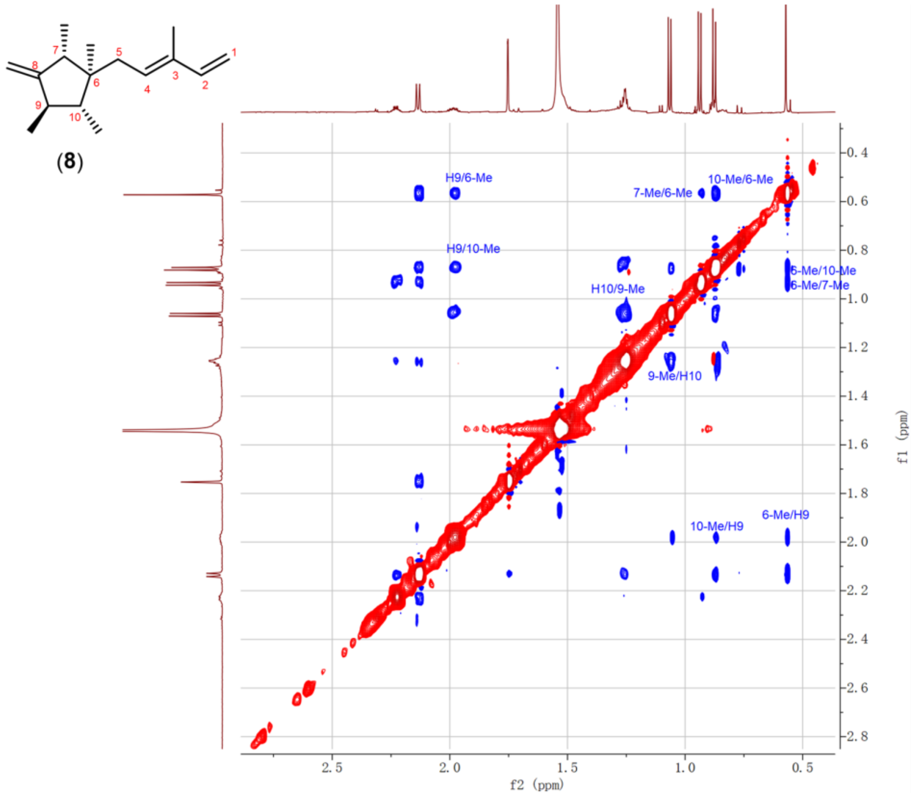

Figure S47. NOESY spectrum of compound 8 in $CDCl_3$.

The decisive part of the NOESY spectrum (below) are zoomed in.

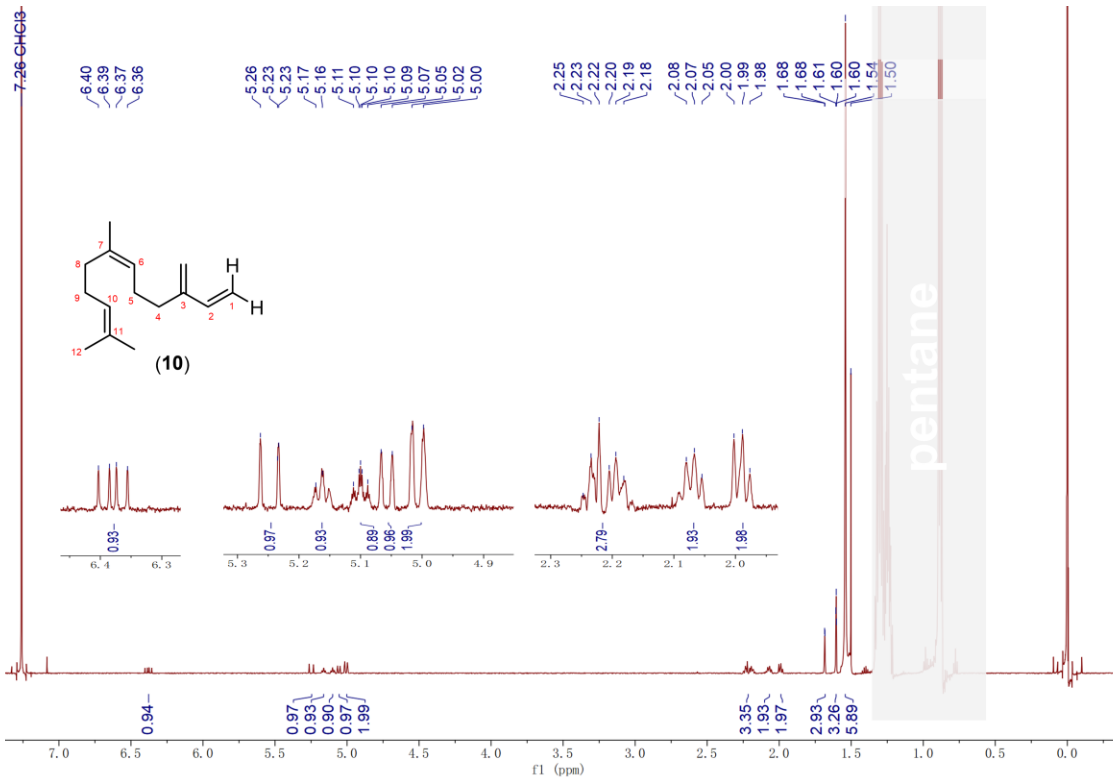

Figure S48. ^{1}H NMR spectrum of compound 10 in $CDCl_3$.

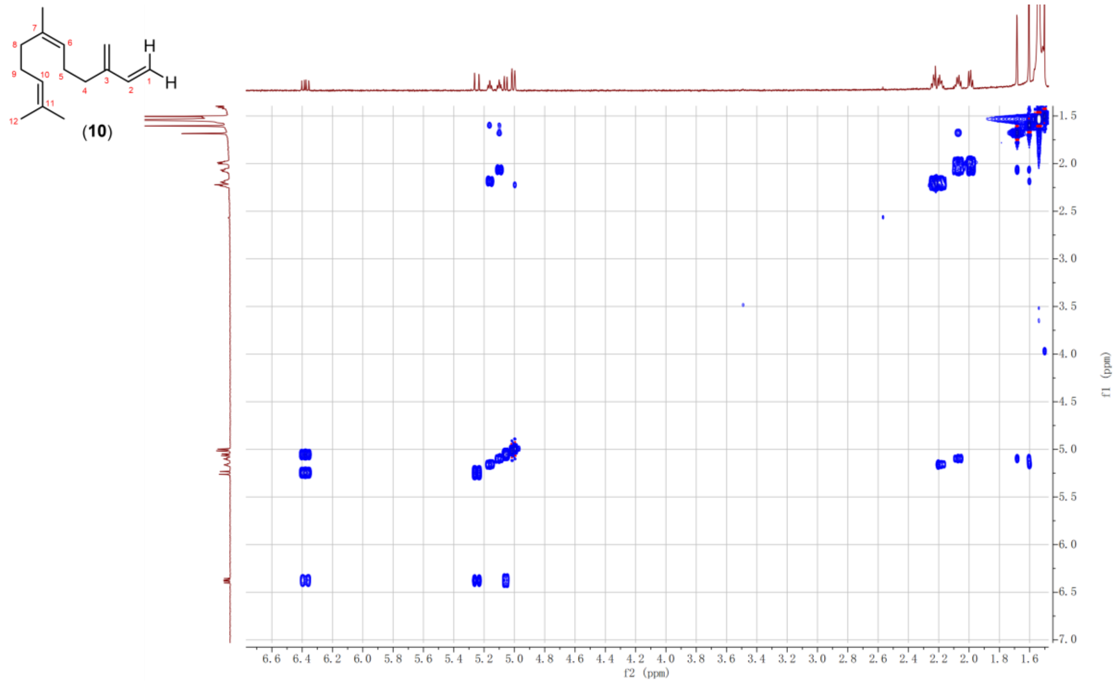

Figure S49. COSY spectrum of compound 10 in $CDCl_3$.

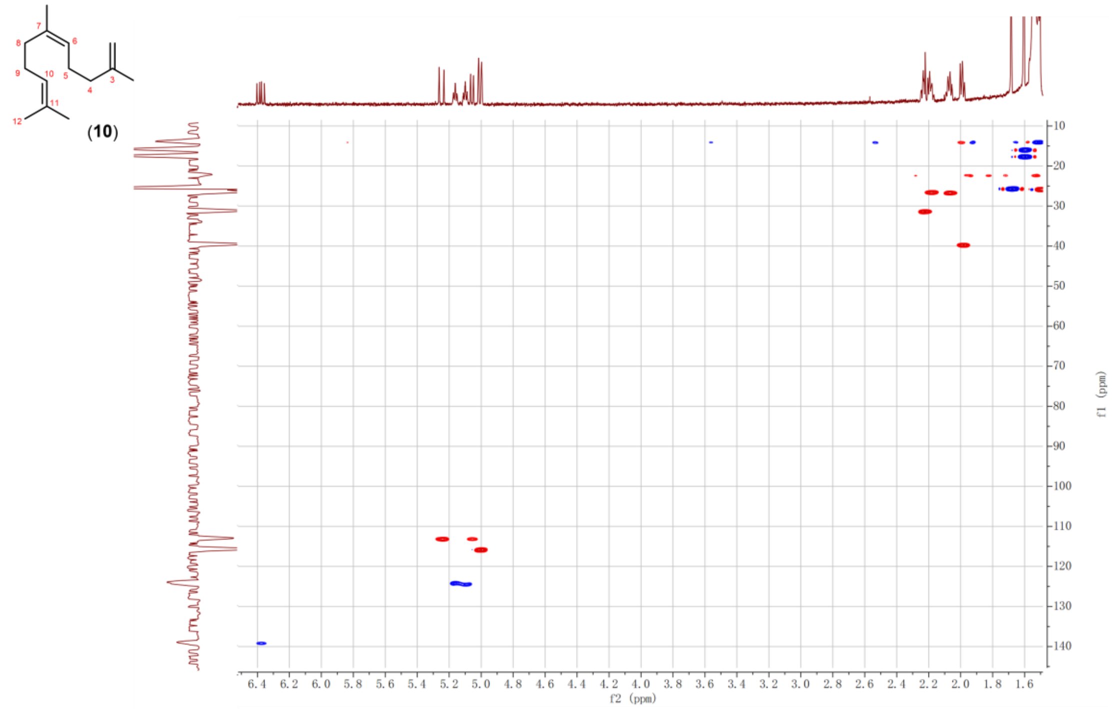

Figure S50. HSQC spectrum of compound 10 in $CDCl_3$.

Carbon chemical shifts were taken from HSQC spectra. Structure elucidation was done by analyzing COSY and HSQC spectra together with GC-MS data. The projections (vertical traces) were generated by extracting the maximum intensity at each chemical shift in the ^{13}C dimension.

Table S10. ^{1}H and ^{13}C NMR data (δ in ppm, *J* in Hz) of compound 11 in C_6D_6.

No.	Types	**11**	
		δ_C	δ_H (*J* in Hz)
1	C	137.43	-
2	C	59.81	-
3	CH_2	32.35	1.18 *ddd* (13.2/9.2/1.6)<br>1.91 *ddd* (13.2/11.4/8.3)
4	CH_2	39.07	1.62 *ddd* (12.5/11.3/9.2)<br>1.49 *ddd* (12.5/8.3/1.6)
5	C	51.14	-
6	CH	147.48	6.16 *ddd* (17.5/11.0/0.5)
7	C	131.39	-
8	CH	59.35	2.09 *m*
9	C	59.01	-
10	CH_2	18.95	1.66-1.72 *m*
1-CH_3	CH_3	9.85	1.46 *dq* (2.7/1.0)
2-CH_3	CH_3	22.05	0.88 *s*
5-CH_3	CH_3	22.74	1.15 *s*
6=CH2	=CH_2	107.68	4.80 *dd* (10.9/1.7)<br>4.89 *dd* (17.5/1.7)
7-CH_3	CH_3	12.42	1.52 *dq* (1.6/1.0)
9-CH_3	CH_3	20.41	1.01 *s*
10-CH_3	CH_3	16.76	1.01 dd (7.7/7.3)

NMR data were recorded at ambient temperature operating at 600.1 MHz for ^{1}H and 150.9 MHz for ^{13}C. Chemical shifts δ are given in ppm relative to TMS. The solvent signals were used as reference (^{1}H: δ_H 7.160 ppm for residual C_6HD_5, ^{13}C: δ_C 128.06 ppm for C_6D_6). Coupling constants are given in Hertz and determined assuming first-order spin-spin coupling unless otherwise stated.

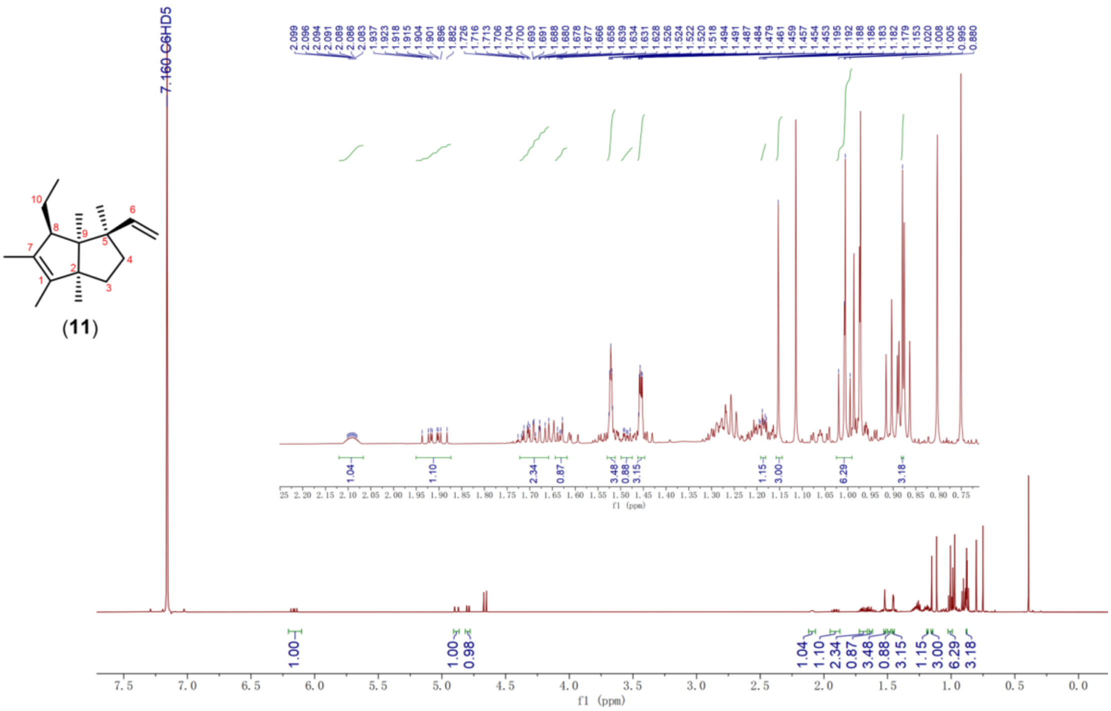

Figure S51. [1]**H NMR spectrum of compound 11 in** C_6D_6**.**

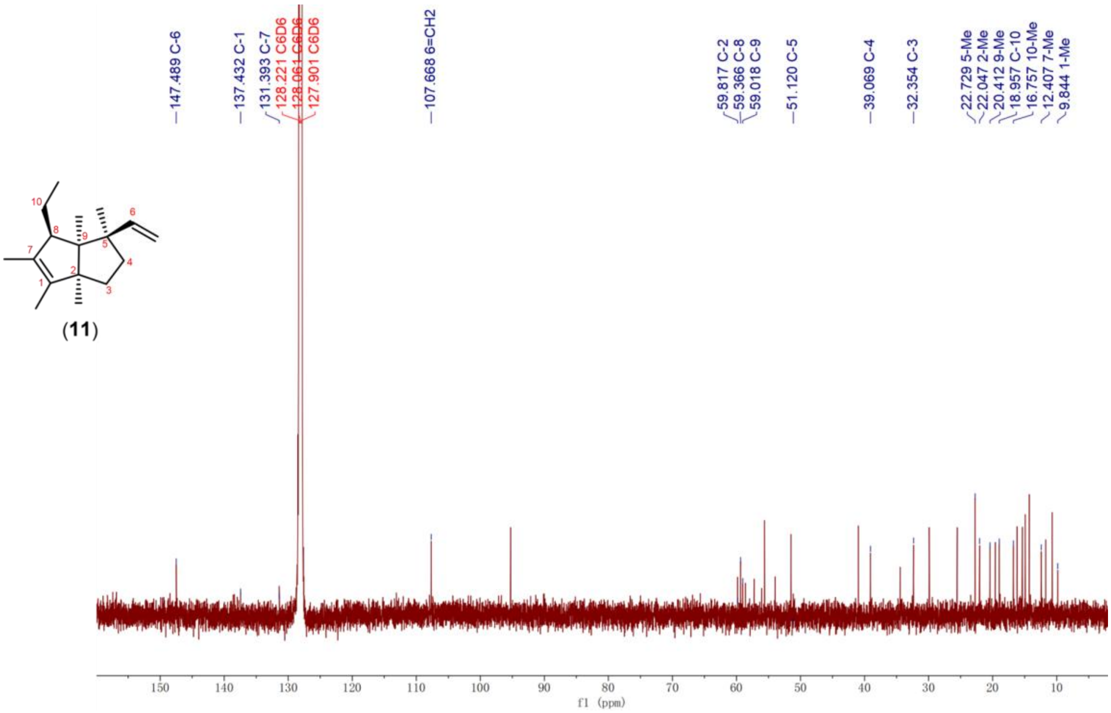

Figure S52. [13]**C NMR spectrum of compound 11 in** C_6D_6**.**

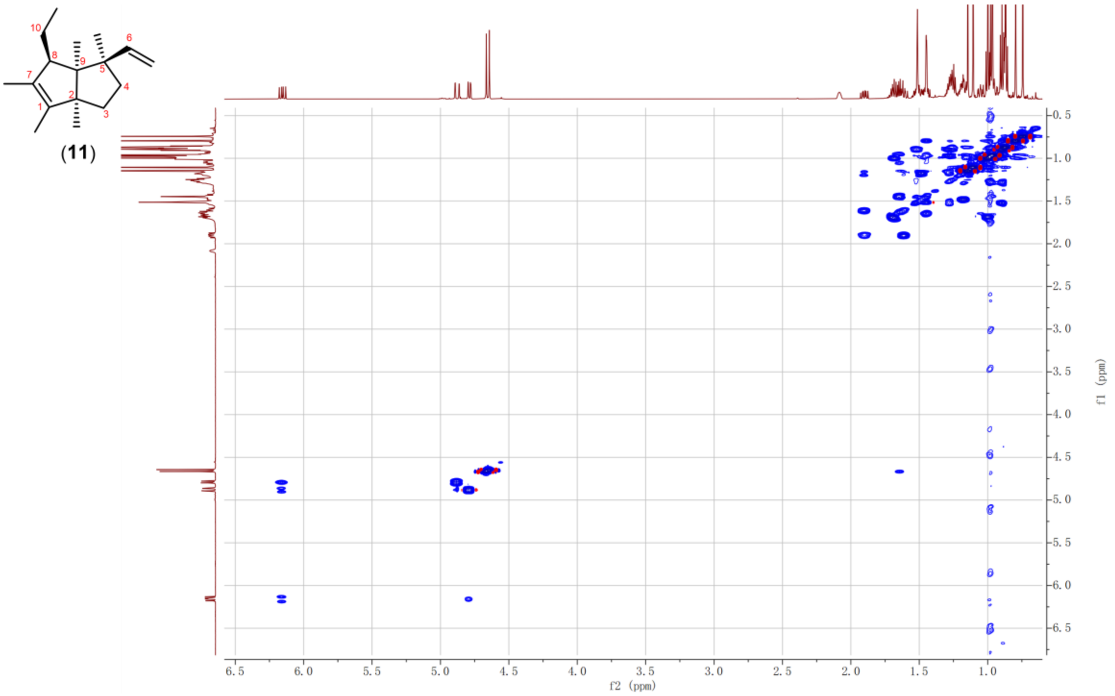

Figure S53. COSY spectrum of compound 11 and compound 12 in C_6D_6.

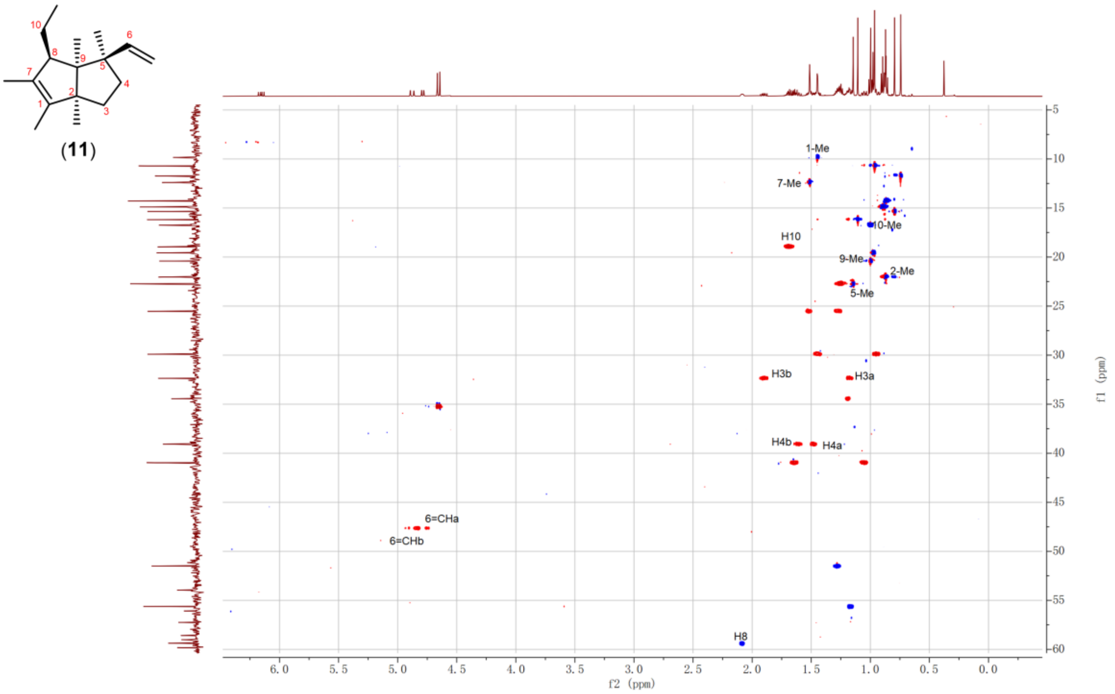

Figure S54. HSQC spectrum of compound 11 in C_6D_6.

The decisive part of the spectrum is zoomed in and the signal corresponding to compound **11** is assigned.

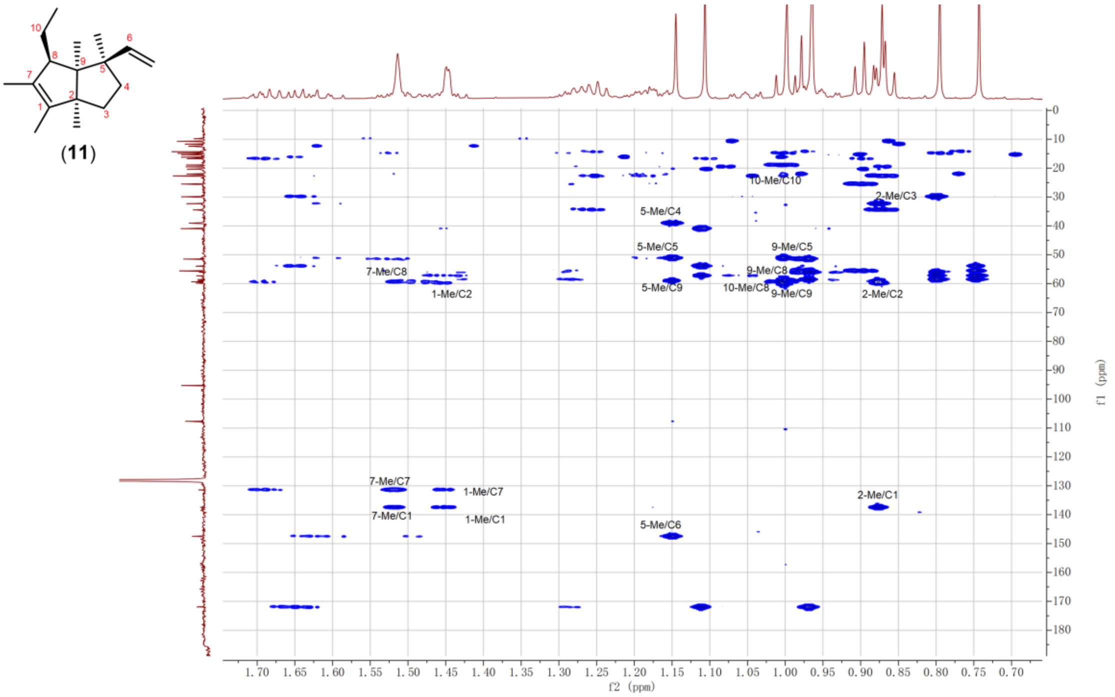

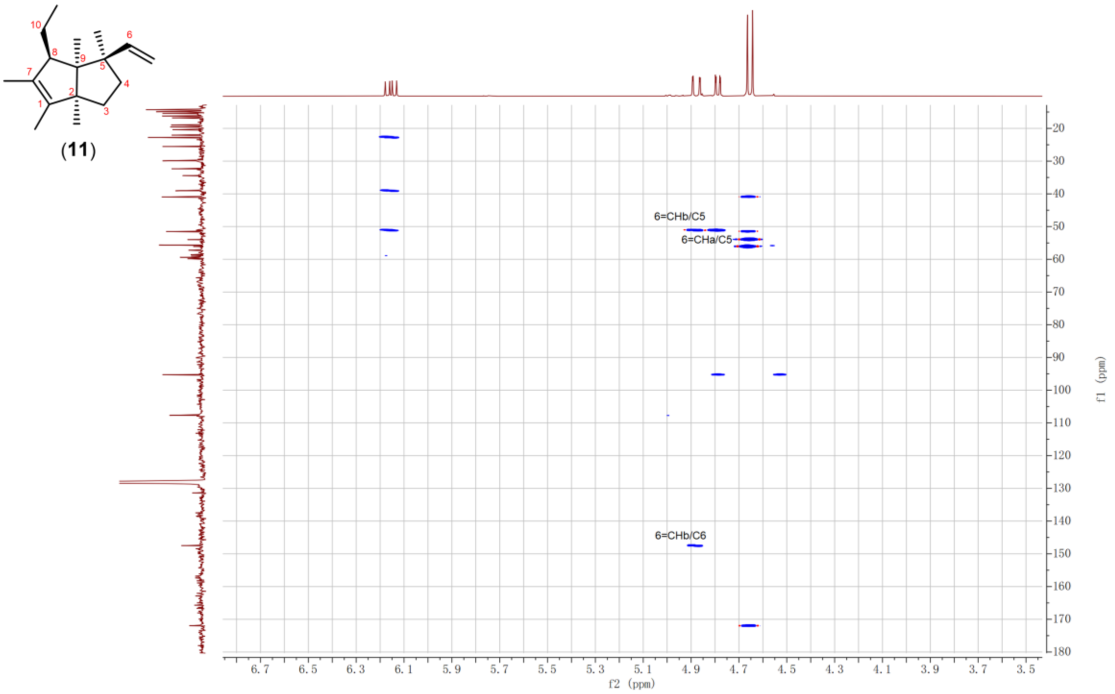

Figure S55. HMBC spectrum of compound 11 in C_6D_6.

The decisive part of the spectrum is zoomed in and the signals corresponding to compound **11** are assigned. The corresponding HMBC correlations for the structural elucidation of compound **11** are shown in Figure S27.

Table S11. ¹H and ¹³C NMR data (δ in ppm, J in Hz) of compound 12 in C_6D_6.

No.	Types	12	
		δ_C	δ_H (J in Hz)
1	C	56.07	-
2	C	58.57	-
3	CH_2	29.90	0.96 *ddd* (13.2/11.5/2.9)<br>1.46 *ddd* (13.2/11.5/2.9)
4	CH_2	40.96	1.06 *ddd* (11.6/9.2/2.8)<br>1.65 *td* (11.3/6.7)
5	C	53.95	-
6	C	171.95	-
7	C	51.49	1.29 *qd* (7.4/6.0)
8	C	55.59	1.17 *m*
9	C	57.24	-
10	CH_2	25.53	1.27 *m*<br>1.53 *m*
1-CH_3	CH_3	10.70	0.97 *d* (0.2)
2-CH_3	CH_3	15.37	0.80 *d* (0.2)
5-CH_3	CH_3	16.19	1.11 *s*
6=CH_2	=CH_2	95.26	4.65 *d* (0.5)<br>4.67 *t* (0.5)
7-CH_3	CH_3	19.58	0.98 *d* (7.4)
9-CH_3	CH_3	11.72	0.75 *s*
10-CH_3	CH_3	14.91	0.90 *dd* (7.6/7.2)

NMR data were recorded at ambient temperature operating at 600.1 MHz for ^1H and 150.9 MHz for ^{13}C. Chemical shifts δ are given in ppm relative to TMS. The solvent signals were used as reference (^1H: δ_H 7.160 ppm for residual C_6HD_5, ^{13}C: δ_C 128.06 ppm for C_6D_6). Coupling constants are given in Hertz and determined assuming first-order spin-spin coupling unless otherwise stated.

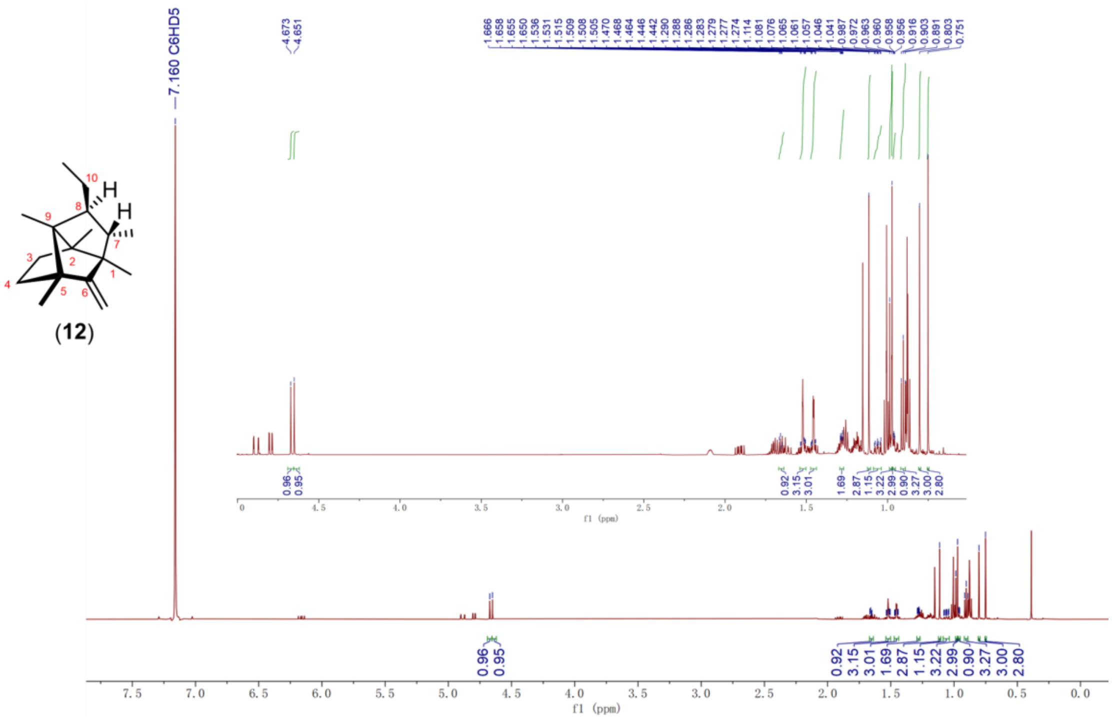

Figure S56. **^{1}H NMR spectrum of compound 12 in C_6D_6.**

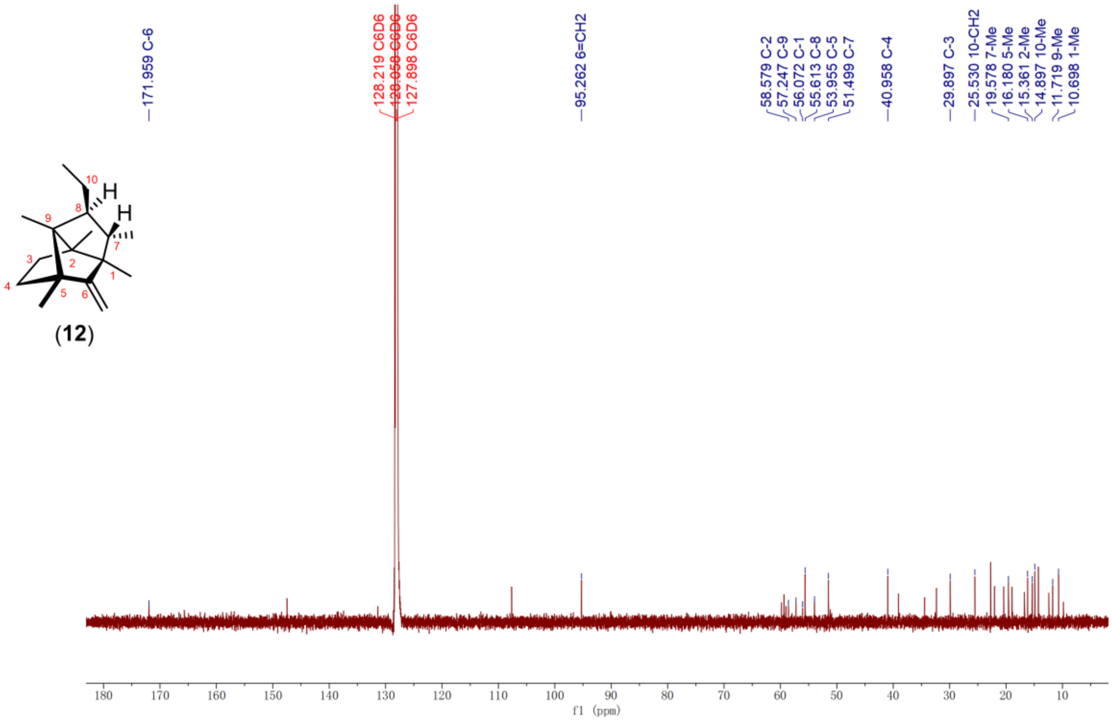

Figure S57. **^{13}C NMR spectrum of compound 12 in C_6D_6.**

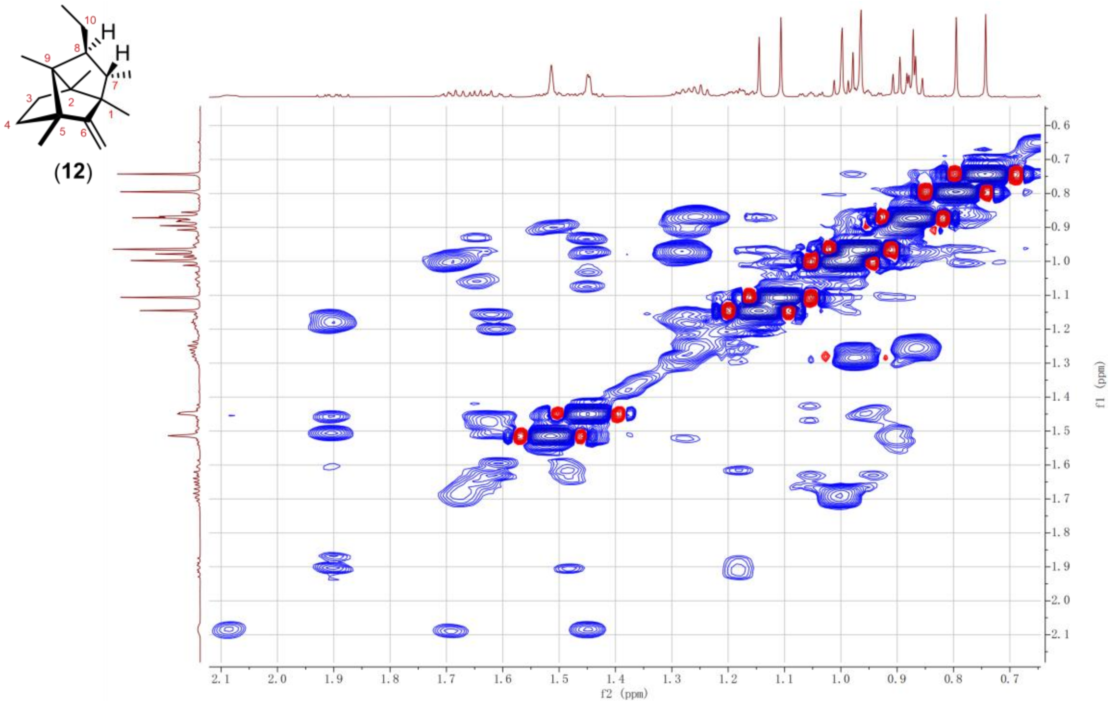

Figure S58. COSY spectrum of compound 12 in C_6D_6.

COSY spectrum of an incompletely separated fraction containing compound **11** and **12**, with an expansion of the decisive part for compound **12**.

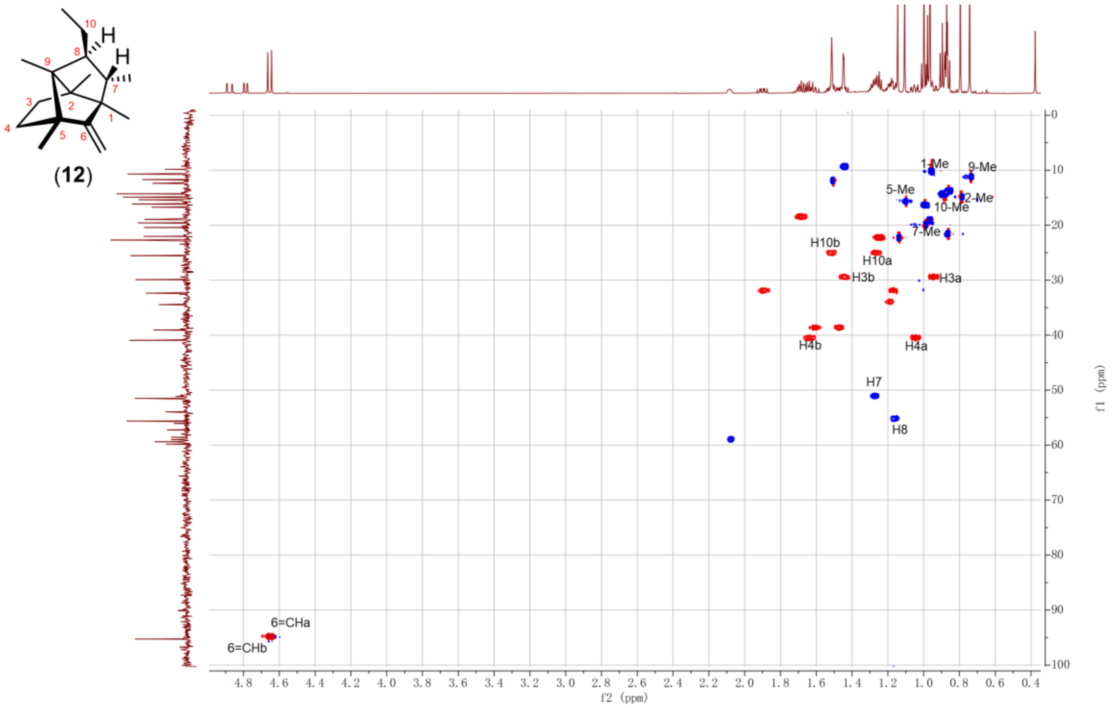

Figure S59. HSQC spectrum of compound 12 in C_6D_6.

The decisive part of the spectrum is zoomed in and the signals corresponding to compound **12** are assigned.

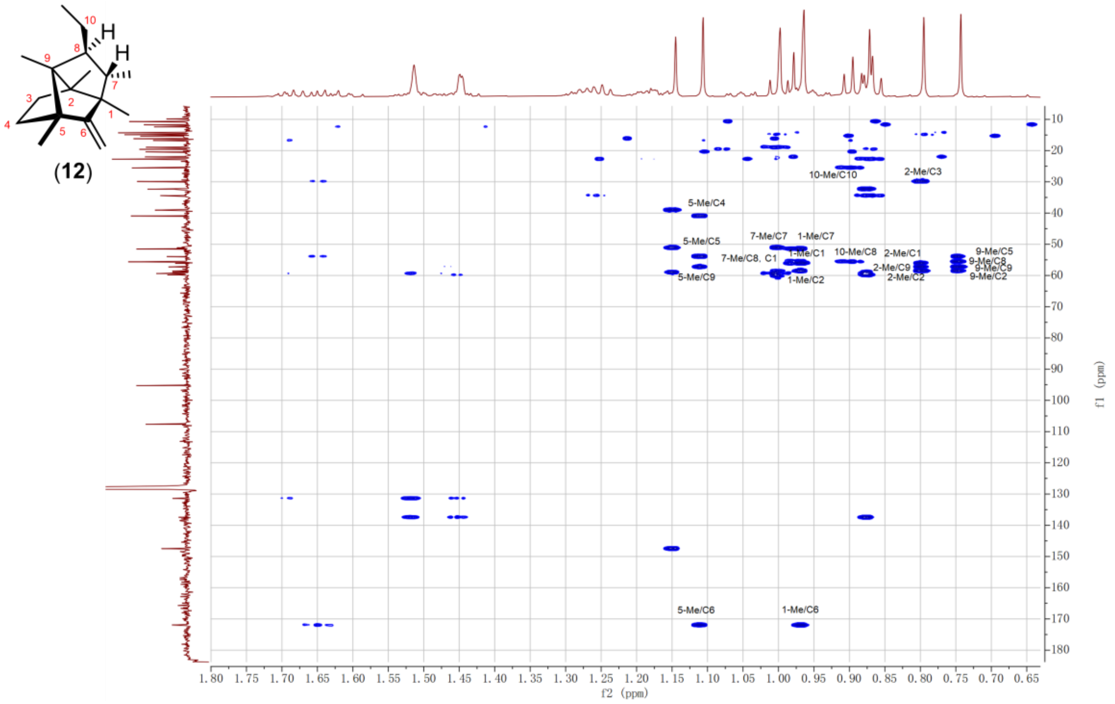

Figure S60. HMBC spectrum of compound 12 in C_6D_6.

The decisive part of the spectrum is zoomed in and the signals corresponding to compound **12** are assigned. The corresponding HMBC correlations for the structural elucidation of compound **12** are shown in Figure S27.

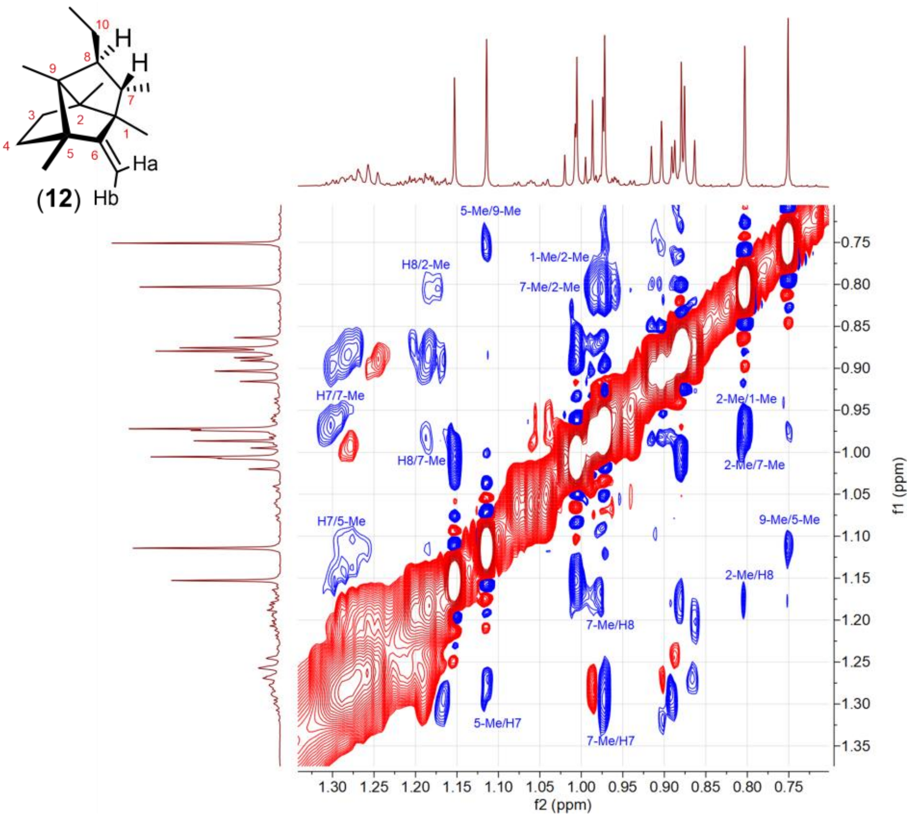

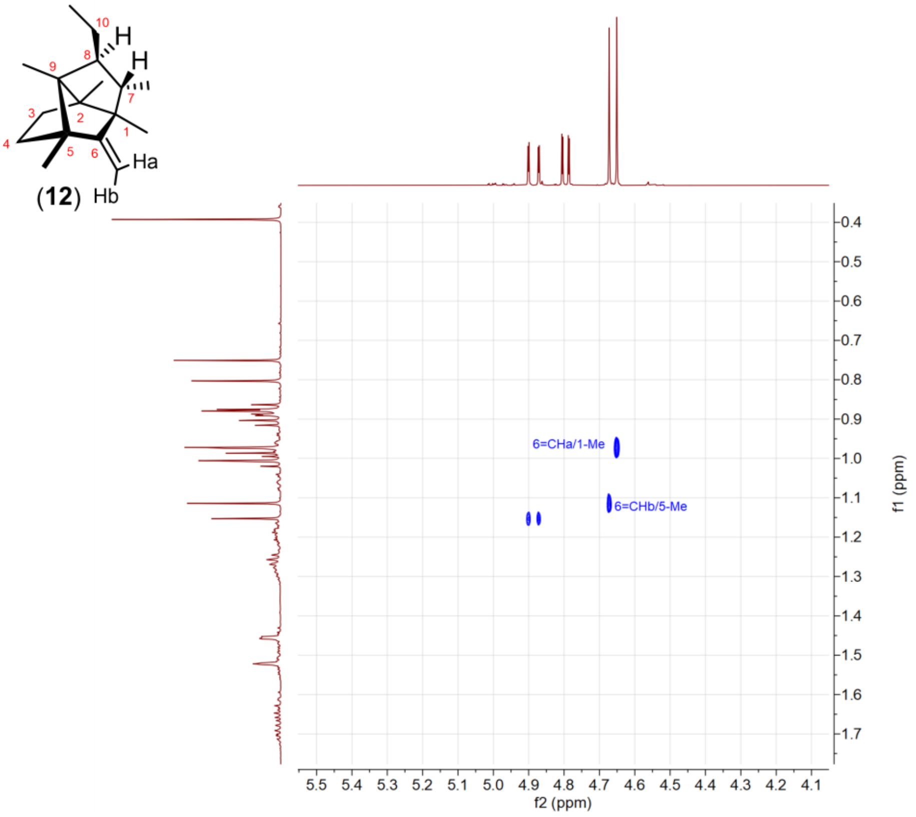

Figure S61. NOESY spectrum of compound 12 in C_6D_6.

The decisive parts of the spectrum are zoomed in and the signal corresponding to compound **12** is assigned.

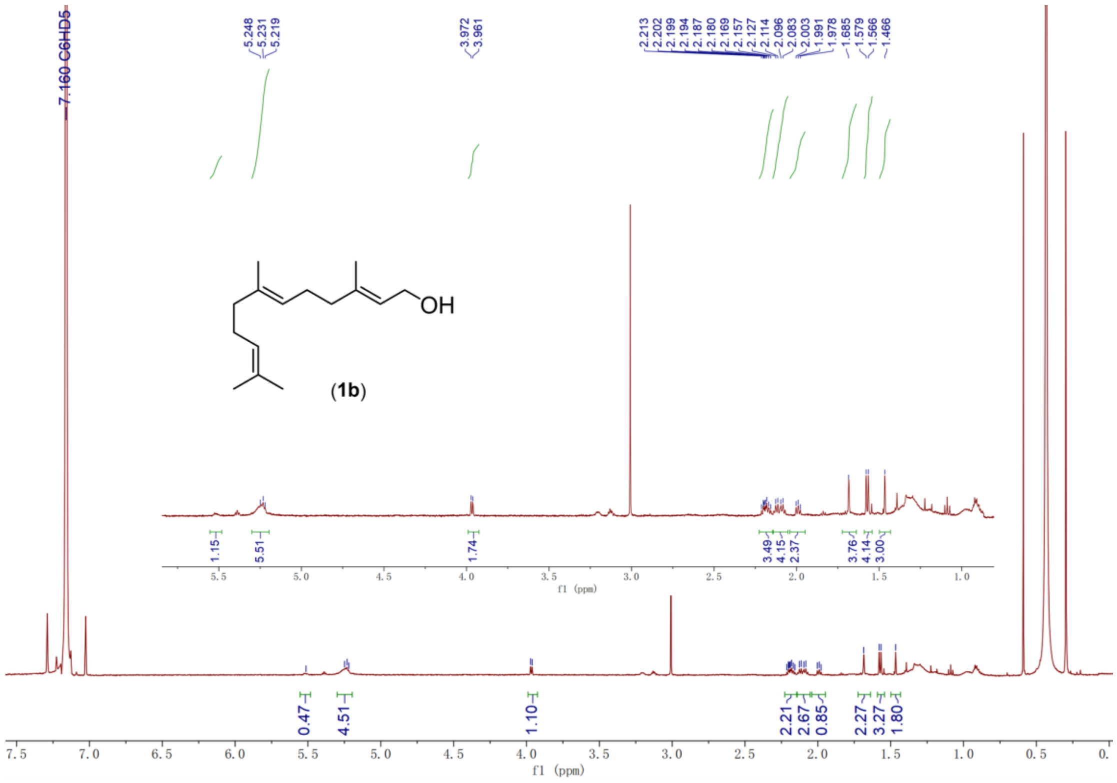

Figure S62. ^{1}H-NMR spectrum of compound 1b in C_6D_6.

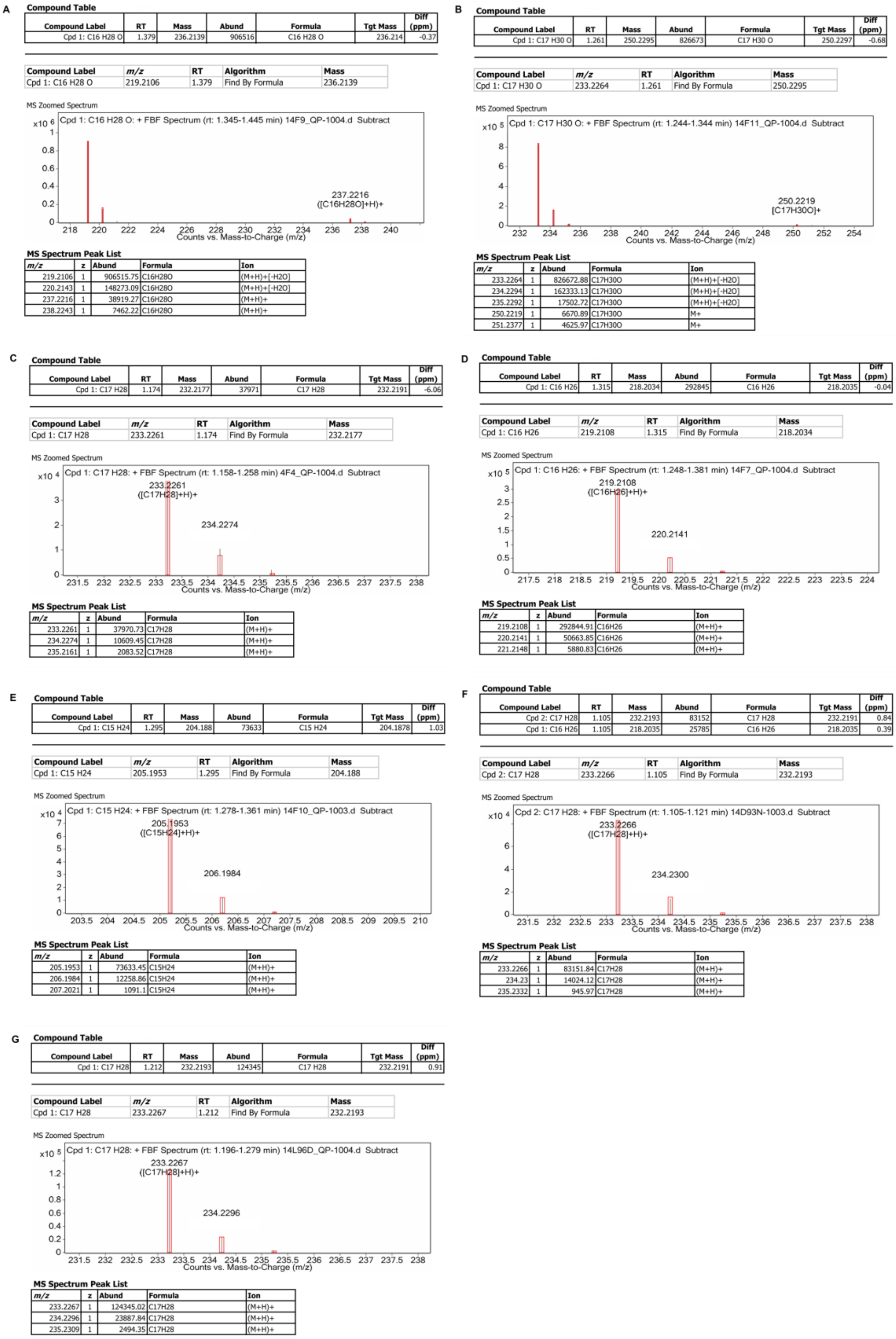

Figure S63. HR-MS spectra of representative compounds by APCI.

High-resolution mass spectra (APCI, positive mode) of compounds (A) **4b**, (B) **5b**, (C) **7**, (D) **8**, (E) **10**, (F) **11**, and (G) **12** showing observed and calculated *m/z* values for the $[M]^+$/$[M+H]^+$ ions. Formula assigned to the monoisotopic peak.

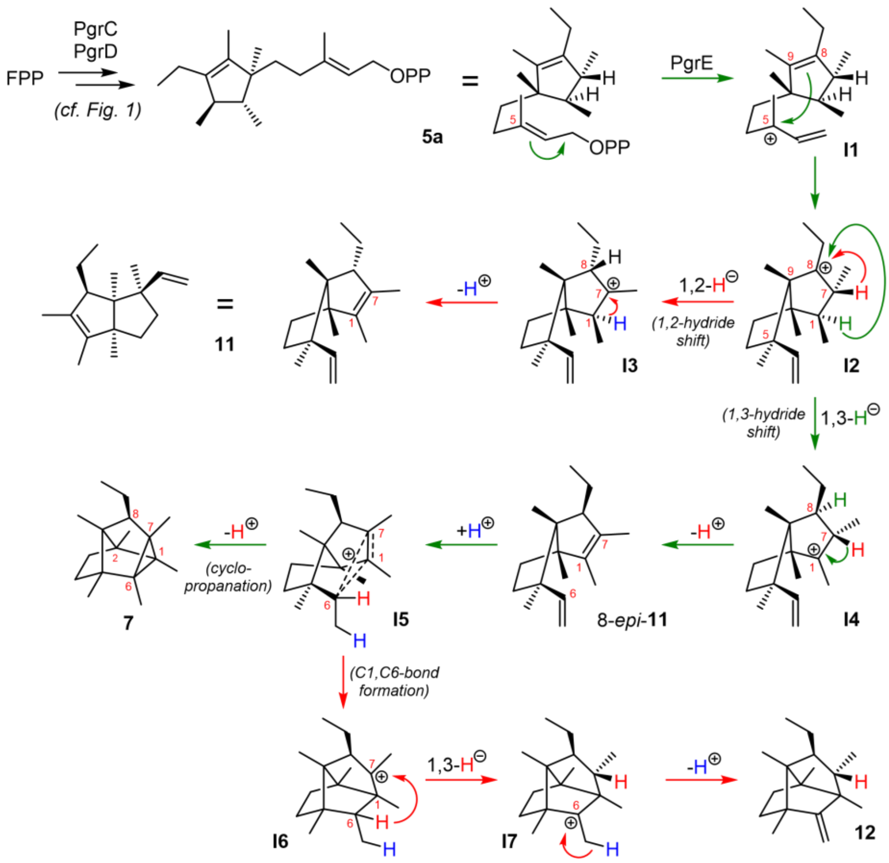

Scheme S64. Proposed biosynthetic pathway from farnesyl pyrophosphate (FPP) to grimophan (7), bicycloprechlororaphen (11), and a chlororaphen analogue (12).

Key intermediate α-PCPP (**5a**) is transformed into joint intermediate **I2**. From there, biosynthesis of **11** versus **7**/**12** diverges. While **11** results from a 1,2-hydride shift to give **I3**, which yields **11** after deprotonation, an alternative 1,3-hydride shift delivers **I4**, which upon deprotonation (8-*epi*-**11**) and protonation to **I5** gives rise to formation of **7** (cyclopropanation) or **I6** (C1,C6-bond formation), with the latter being further processes to **12**. The proposed mechanism is inspired by the comprehensive mechanistic analysis of chlororaphen biosynthesis reported by the Dickschat laboratory.[14]

References

(1) Huerta-Cepas, J.; Serra, F.; Bork, P. ETE 3: Reconstruction, Analysis, and Visualization of Phylogenomic Data. *Mol. Biol. Evol.* **2016**, *33* (6), 1635–1638. https://doi.org/10.1093/molbev/msw046.

(2) Mo, X.-H.; Pu, Q.-Y.; Lübken, T.; Yu, G.-H.; Malay, M.; D'Agostino, P. M.; Gulder, T. A. M. Discovery and Biosynthesis of Non-Canonical C16-Terpenoids from Pseudomonas. *Cell Chem. Biol.* **2024**, *31* (12), 2128-2137.e4. https://doi.org/10.1016/j.chembiol.2024.09.002.

(3) Tang, X.; Demiray, M.; Wirth, T.; Allemann, R. K. Concise Synthesis of Artemisinin from a Farnesyl Diphosphate Analogue. *Bioorg. Med. Chem.* **2018**, *26* (7), 1314–1319. https://doi.org/10.1016/j.bmc.2017.03.068.

(4) Davisson, V. J.; Woodside, A. B.; Neal, T. R.; Stremler, K. E.; Muehlbacher, M.; Poulter, C. D. Phosphorylation of Isoprenoid Alcohols. *J. Org. Chem.* **1986**, *51* (25), 4768–4779. https://doi.org/10.1021/jo00375a005.

(5) Mai, P.; Zocher, G.; Stehle, T.; Li, S.-M. Structure-Based Protein Engineering Enables Prenyl Donor Switching of a Fungal Aromatic Prenyltransferase. *Org. Biomol. Chem.* **2018**, *16* (40), 7461–7469. https://doi.org/10.1039/C8OB02037J.

(6) Harris, R. K.; Becker, E. D.; Menezes, S. M. C. de; Goodfellow, R.; Granger, P. NMR Nomenclature. Nuclear Spin Properties and Conventions for Chemical Shifts (IUPAC Recommendations 2001). *Pure Appl. Chem.* **2001**, *73* (11), 1795–1818. https://doi.org/10.1351/pac200173111795.

(7) Abramson, J.; Adler, J.; Dunger, J.; Evans, R.; Green, T.; Pritzel, A.; Ronneberger, O.; Willmore, L.; Ballard, A. J.; Bambrick, J.; Bodenstein, S. W.; Evans, D. A.; Hung, C.-C.; O'Neill, M.; Reiman, D.; Tunyasuvunakool, K.; Wu, Z.; Žemgulytė, A.; Arvaniti, E.; Beattie, C.; Bertolli, O.; Bridgland, A.; Cherepanov, A.; Congreve, M.; Cowen-Rivers, A. I.; Cowie, A.; Figurnov, M.; Fuchs, F. B.; Gladman, H.; Jain, R.; Khan, Y. A.; Low, C. M. R.; Perlin, K.; Potapenko, A.; Savy, P.; Singh, S.; Stecula, A.; Thillaisundaram, A.; Tong, C.; Yakneen, S.; Zhong, E. D.; Zielinski, M.; Žídek, A.; Bapst, V.; Kohli, P.; Jaderberg, M.; Hassabis, D.; Jumper, J. M. Accurate Structure Prediction of Biomolecular Interactions with AlphaFold 3. *Nature* **2024**, 1–3. https://doi.org/10.1038/s41586-024-07487-w.

(8) Laskowski, R. A. PDBsum1: A Standalone Program for Generating PDBsum Analyses. *Protein Sci. Publ. Protein Soc.* **2022**, *31* (12), e4473. https://doi.org/10.1002/pro.4473.

(9) Schrödinger, LLC. The PyMOL Molecular Graphics System, Version 1.8, 2015.

(10) Park, T.; Won, J.; Baek, M.; Seok, C. GalaxyHeteromer: Protein Heterodimer Structure Prediction by Template-Based and Ab Initio Docking. *Nucleic Acids Res.* **2021**, *49* (W1), W237–W241. https://doi.org/10.1093/nar/gkab422.

(11) Pfeifer, B. A.; Admiraal, S. J.; Gramajo, H.; Cane, D. E.; Khosla, C. Biosynthesis of Complex Polyketides in a Metabolically Engineered Strain of E. Coli. *Science* **2001**, *291* (5509), 1790–1792. https://doi.org/10.1126/science.1058092.

(12) Liao, C.; Seebeck, F. P. S-Adenosylhomocysteine as a Methyl Transfer Catalyst in Biocatalytic Methylation Reactions. *Nat. Catal.* **2019**, *2* (8), 696–701. https://doi.org/10.1038/s41929-019-0300-0.

(13) Duell, E. R.; D'Agostino, P. M.; Shapiro, N.; Woyke, T.; Fuchs, T. M.; Gulder, T. A. M. Direct Pathway Cloning of the Sodorifen Biosynthetic Gene Cluster and Recombinant Generation of Its Product in E. Coli. *Microb. Cell Factories* **2019**, *18* (1), 32. https://doi.org/10.1186/s12934-

019-1080-6.

(14) Xu, H.; Li, H.; Goldfuss, B.; Schnakenburg, G.; Dickschat, J. S. Biosynthesis of the Non-Canonical C17 Sesquiterpenoids Chlororaphen A and B from Pseudomonas Chlororaphis. *Angew. Chem. Int. Ed.* **2024**, *63* (43), e202412040. https://doi.org/10.1002/anie.202412040.
